# Tistrellabactins A and B Are Photoreactive *C*-Diazeniumdiolate Siderophores from the Marine-Derived Strain *Tistrella mobilis* KA081020-065

**DOI:** 10.1021/acs.jnatprod.3c00230

**Published:** 2023-06-21

**Authors:** Christina Makris, Jamie K. Leckrone, Alison Butler

**Affiliations:** Department of Chemistry & Biochemistry, University of California, Santa Barbara, California 93106-9510, United States

## Abstract

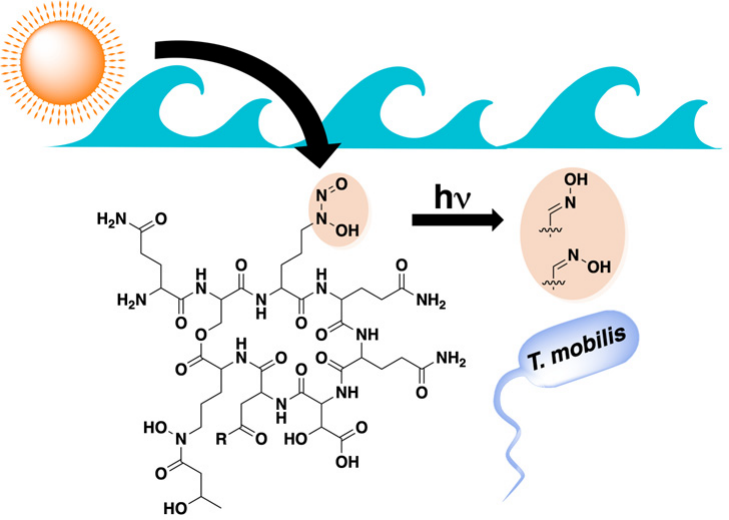

The *C*-diazeniumdiolate group in the amino acid graminine is emerging as a new microbially produced Fe(III) coordinating ligand in siderophores, which is photoreactive. While the few siderophores reported from this class have only been isolated from soil-associated microbes, here we report the first *C*-diazeniumdiolate siderophores tistrellabactins A and B, isolated from the bioactive marine-derived strain *Tistrella mobilis* KA081020-065. The structural characterization of the tistrellabactins reveals unique biosynthetic features including an NRPS module iteratively loading glutamine residues and a promiscuous adenylation domain yielding either tistrellabactin A with an asparagine residue or tistrellabactin B with an aspartic acid residue at analogous positions. Beyond the function of scavenging Fe(III) for growth, these siderophores are photoreactive upon irradiation with UV light, releasing the equivalent of nitric oxide (NO) and an H atom from the *C*-diazeniumdiolate group. Fe(III)-tistrellabactin is also photoreactive, with both the *C*-diazeniumdiolate and the β-hydroxyaspartate residues undergoing photoreactions, resulting in a photoproduct without the ability to chelate Fe(III).

The synthesis of N–N bonds are chemically challenging, yet microbes have mastered this transformation, producing hundreds of structurally complex natural products with an N–N bond.^1^ Reports of natural products with N–N linkages and their biosyntheses have garnered significant recent attention,^2−12^ including the *C*-type diazeniumdiolate amino acid, graminine (Gra, Figure 1).^13,14^l-Gra was recently shown to originate from l-Arg,^14^ although the mechanism of this oxidative rearrangement has not yet been elucidated. The *C*-diazeniumdiolate group has emerged as a new class of Fe(III) binding ligands in microbial siderophores,^13,15^ adding to the well-established catecholate, hydroxamate, and α-hydroxycarboxylate groups. Siderophores are small-molecule microbial natural products that have evolved to facilitate iron sequestration and microbial iron uptake. Biological competition for available iron has driven the evolution of a large selection of siderophores, with hundreds identified thus far, yet siderophores with a *C*-diazeniumdiolate ligand have only just begun to be uncovered.^13,15−17^ Up to this point, graminine-containing siderophores have only been isolated from soil-associated microbes within the related Burkholderiaceae family, including gramibactin (Gbt, Figure 1) from *Paraburkholderia graminis* DSM 17151.^13,15,18^

**Figure 1:**
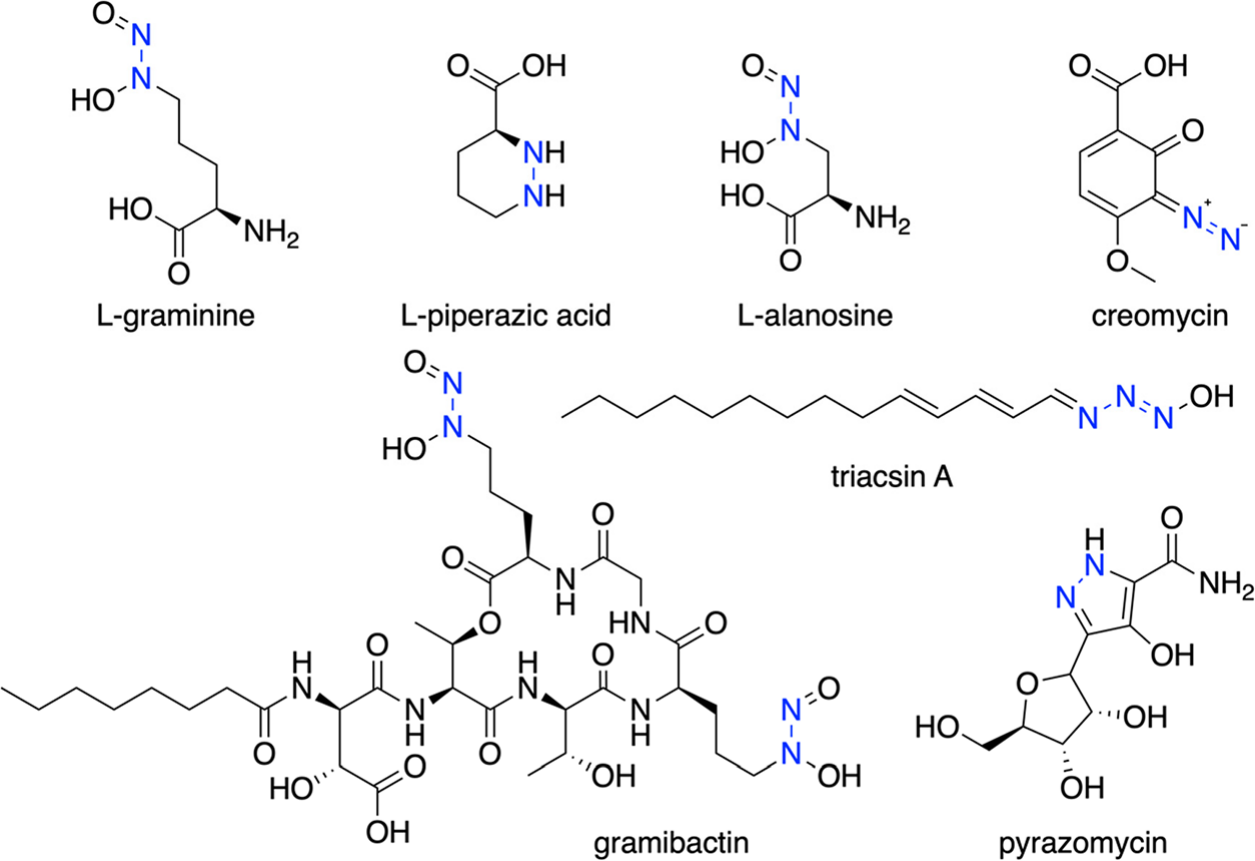
Select natural products with N–N bond linkages, highlighted in blue.

The N–N bond in the diazeniumdiolate moiety is particularly intriguing in the context of nitric oxide (NO) release.^14^*N*-Type diazeniumdiolates (NONOates) lose two equivalents of NO under physiological conditions,^19^ while *C*-diazeniumdiolates are more thermally stable. We recently demonstrated that *C*-diazeniumdiolate siderophore Gbt releases the equivalent of NO and an H atom for each Gra residue when irradiated with UV light, producing a mixture of *E* and *Z* oxime isomers as the photoproduct.^14^ Additionally, peroxidase-mediated NO release from Gbt has been reported.^15^ Given the relevance of NO and oximes in physiological signaling pathways^19,20^ and defensive responses,^21,22^ this ligand system may serve multiple functions within the microbe beyond Fe(III) scavenging.

The biosynthetic genes encoding the enzymes that synthesize l-Gra have been identified in the biosynthetic gene cluster (BGC) for gramibactin as *grbD* and *grbE* through gene knockout studies with the strain *P. graminis* DSM 17151.^15^ GrbE shares sequence homology to several Arg hydroxylases (DcsA,^23^ Mhr24,^24^ AglA^25^), while GrbD shares sequence homology to the cupin domain of the N–N bond, forming enzyme SznF^7^/StzF^4^, which carries out the oxidative rearrangement of *N*^δ^-hydroxy-*N*^ω^-hydroxy-*N*^ω^-methyl-l-Arg. Targeted discovery of new *C*-diazeniumdiolate siderophores is achievable by searching microbial genomes containing a nonribosomal peptide synthetase (NRPS) adenylation (A) domain with the specificity code for Gra (DVHRTGLVAK) and FASTA sequences of *grbD* and *grbE* as queries. With this strategy, genome mining efforts reveal a widespread prevalence of l-Gra in natural products from a variety of environments, including clinical isolates, plant pathogens, and, as described in this study, a marine-derived microbe.

We report herein *Tistrella mobilis* KA081020-065, isolated from the Red Sea, produces *C*-diazeniumdiolate siderophores tistrellabactins A and B. The tistrellabactins are photoreactive, losing NO in UV light, including in actively growing cultures of *T. mobilis*. Surprisingly, the tistrellabactin NRPS enzymes load glutamine residues iteratively via an unknown mechanism, which is also observed in the biosynthesis of the anticancer didemnin natural products produced by *T. mobilis* KA081020-065.^26^ The characterization of tistrellabactins A and B reveals other unusual biosynthetic features, including a promiscuous NRPS A domain, a 22-membered macrolactone ring, and a 3-hydroxybutyric acid group appended to *N*-hydroxy-l-ornithine.

## Results and Discussion

### Genome Mining Reveals a Biosynthetic Gene Cluster of a *C*-Type Diazeniumdiolate Siderophore in a Marine-Derived Strain

**Figure 2:**
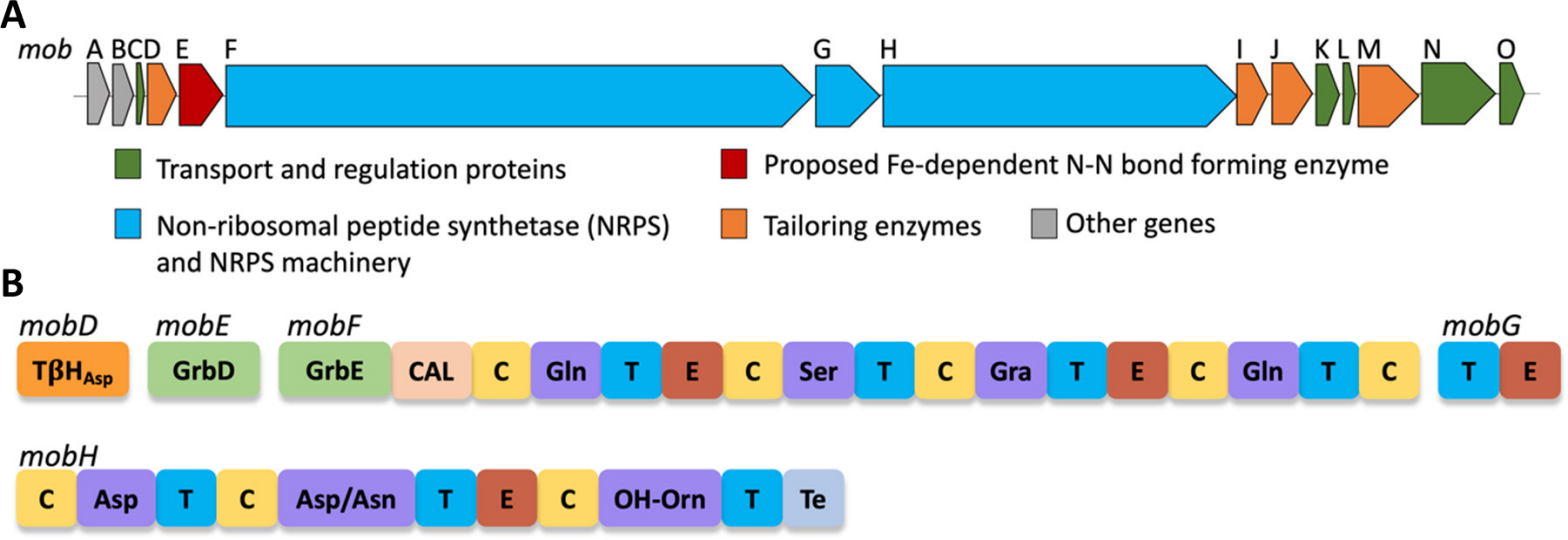
Tistrellabactins A and B are mixed-ligand siderophores isolated from the marine-derived strain *T. mobilis* KA081020-065. (A) BGC of tistrellabactins A and B encodes sequence homologues of *grbD* (*mobE*) and *grb*E (fused to the start of *mobF*), which are implicated in the biosynthesis of the graminine residues N-monooxygenase (MobI) and *N*-acetyltransferase (MobJ), which install the hydroxy and acetyl groups on l-Orn, and a TβH_Asp_ hydroxylase (MobD), which hydroxylates aspartic acid. (B) NRPS module breakdown of MobF, MobG, and MobH. MobG iteratively loads a second Gln residue. MobH has a promiscuous A domain, which can activate l-Asn or l-Asp, yielding tistrellabactins A and B, respectively. Abbreviations: coenzyme ligase A domain (CAL), condensation domain (C), thiolation domain (T), epimerization domain (E), thioesterase (Te).

A putative siderophore BGC with gene analogs of *grbD* and *grbE*, consistent with incorporation of l-Gra, was identified on plasmid 2 (accession: NC_017966.1, Figure 2A, Table S1).^26^ For targeted discovery of the metabolite, the FASTA sequence of the A domain specific for Gra was used as the query in NCBI BLASTP (basic local alignment search tool-protein). Hits were then narrowed to microbes that included biosynthetic gene clusters with siderophore-related genes, an A domain with the specificity code for l-Gra, and sequence homologues of the proposed Gra biosynthesis genes, *grbD* and *grbE*, from the BGC of gramibactin.^13^

*C*-Diazeniumdiolate siderophores currently reported in the literature are all produced by soil-associated microbes, which raises the question if they are also produced by microbes in other environments.^13,15^ To investigate the extent of this siderophore type beyond the terrestrial soil environment, the family Burkholderiaceae was excluded from the genome search. The presence of GrbD and GrbE homologues, alongside an NRPS protein containing an A domain with a specificity code for Gra, indicated marine-derived *T. mobilis* KA081020-065 should incorporate Gra with a *C*-diazeniumdiolate for Fe(III) coordination (Figure 2).

### Discovery and Structural Elucidation of Tistrellabactins A and B

**Figure 3:**
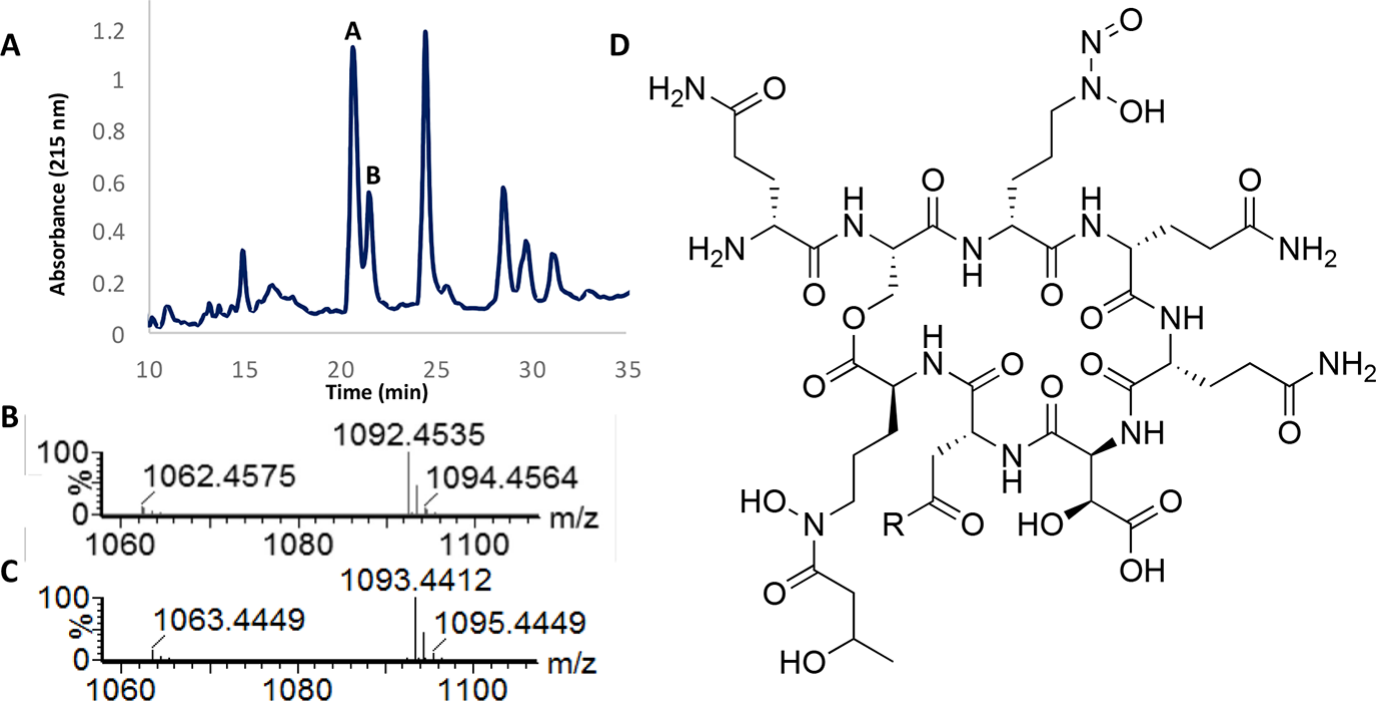
(A) HPLC chromatogram trace (215 nm) showing tistrellabactin A and tistrellabactin B elute side by side in 20% aqueous MeOH + 0.05% TFA, with favored production of tistrellabactin A to tistrellabactin B. (B) Tistrellabactin A (*m*/*z* 1092.4 [M + H]^+^) and (C) tistrellabactin B (*m*/*z* 1093.4 [M + H]^+^) differ by one mass unit, and each displays an ionization mass loss of 30 Da, characteristic of *C*-diazeniumdiolates. (D) The structures of isolated and purified tistrellabactin A (R = NH_2_) and tistrellabactin B (R = OH) were elucidated by NMR spectroscopy and MS.

*T. mobilis* KA081020-065 was cultured in an artificial seawater medium under Fe-limited conditions to induce siderophore production. Analysis of purified compounds from the culture extract with a positive colorimetric Fe-CAS assay response by UPLC-ESI-MS revealed two putative *C*-diazeniumdiolate siderophore masses, *m*/*z* 1092.4 and 1093.4 [M + H]^+^, differing by a single mass unit and both with a characteristic mass loss of 30 Da consistent with ionization of the N–N bond in graminine (Figures 2, S1). Given the mass similarity of the metabolites and the presence of only one siderophore gene cluster in the genome of *T. mobilis* KA081020-065, the compounds appear to be congeners. The first-eluting peak corresponds with the protonated molecule *m*/*z* 1092.4 [M + H]^+^ and the latter to *m*/*z* 1093.4 [M + H]^+^, which will herein be referred to as tistrellabactin A and tistrellabactin B, respectively (Figure 3).

The empirical formulas of tistrellabactins A and B were assigned by high-resolution mass spectrometry (HR-MS) to be C_40_H_65_N_15_O_21_ and C_40_H_64_N_14_O_22_, respectively (Figures S2 and S3). The structures of tistrellabactins A and B were elucidated by complete assignment of all ^1^H, ^13^C, and ^15^N resonances (Figures 4A, S4–S20, Tables S2, S3, S4). Overlapping methylene ^1^H resonances and several sets of diastereotopic ^1^H resonances were differentiated using 2D NMR experiments, primarily multiplicity edited HSQC and TOCSY (Figures S6, S9, S15, S18).

**Figure 4:**
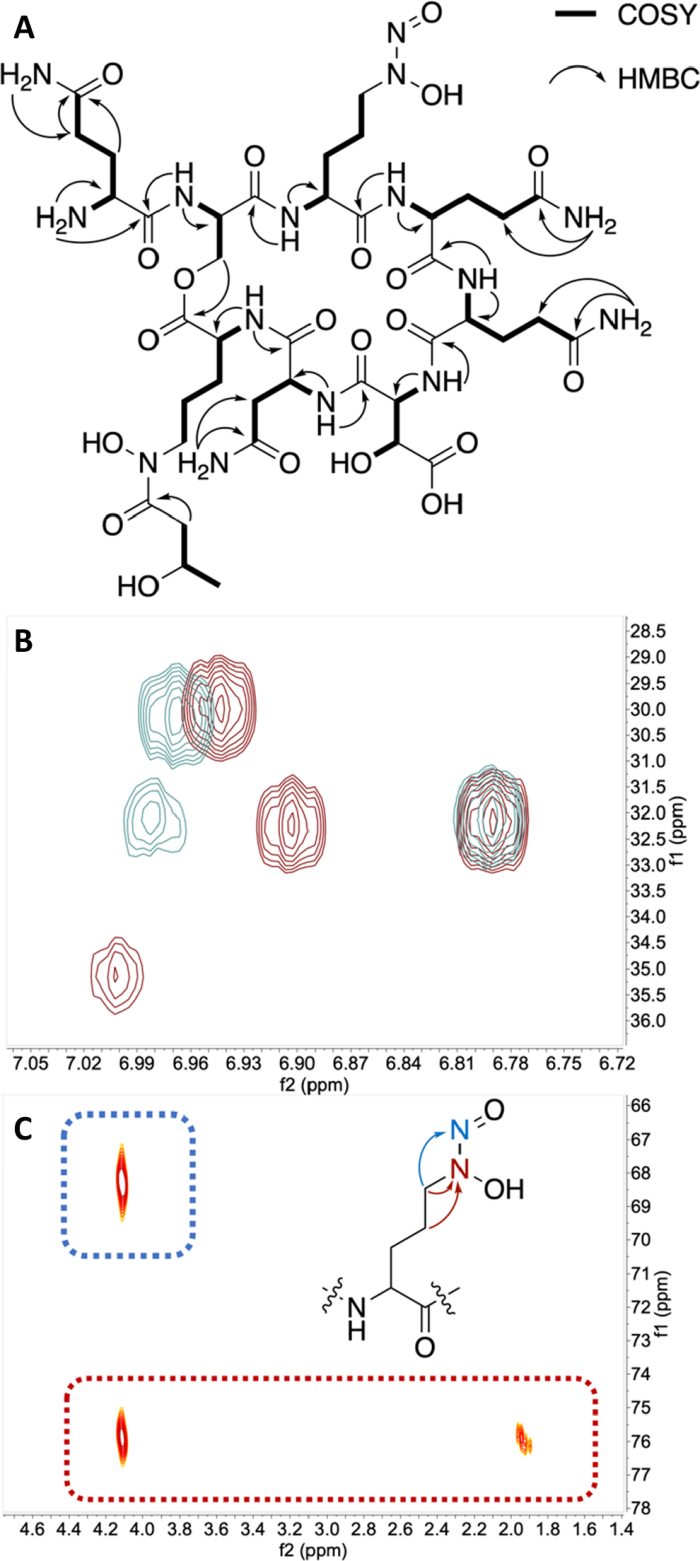
NMR-guided structure elucidation of tistrellabactins A and B. (A) Key ^1^H–^13^C HMBC and ^1^H–^1^H COSY correlations in tistrellabactin A. (B) Superimposed tistrellabactin A (maroon) and tistrellabactin B (blue) ^1^H–^13^C HMBC correlations show three Gln *N*H_2_ side chain ^1^H pairs correlate to respective Gln *C*γ. An HMBC correlation between Asn side chain NH_2_^1^H at 6.99 ppm and Cβ ^13^C at 34.85 ppm is not present in tistrellabactin B, which has an Asp residue in the analogous position. (C) ^1^H–^15^N HMBC shows the presence of *C*-diazeniumdiolate amino acid Gra.

Three discrete ^1^H spin systems were attributed to the Gln side chain amine protons in tistrellabactins A and B.^27^ An additional discrete ^1^H spin system present in tistrellabactin A only was attributed to the Asn side chain.^27^ Through-bond ^1^H–^13^C HMBC correlations connect the side chain amine ^1^H resonances to methylene protons of three Gln and one Asn in tistrellabactin A only (Figures 4B, S7, S16). Observed in the TOCSY spectrum was a ^1^H spin system assigned to a 3-hydroxybutyric acid group (Hbu). Correlations between the hydroxylated ^15^N on the Orn side chain and the Hbu group established the 3-hydroxybutyric acid is appended to the l-Orn residue, forming a hydroxamate ligand. This rare acyl group has been seen in the siderophore structures of cupriachelin and imaqobactin.^28,29^ The presence of the Gra residue, which has a distinct ^1^H–^15^N HMBC fingerprint, was confirmed with the presence of correlations between the Gra Cδ methylene protons to both nitrogens in the diazeniumdiolate group and Cγ methylene protons to the hydroxylated nitrogen only (Figure 4C).

The Asn in tistrellabactin A is replaced by an Asp in tistrellabactin B, consistent with the mass difference of 1 Da between them. Connectivity of the peptidic backbone was determined based on ^1^H–^13^C HMBC correlations across amide bonds between adjacent amino acids, with cyclization between the C-terminus carboxylic acid of Orn to the Ser residue, forming an ester linkage (Figures S7, S16). The diastereotopic Cβ ^1^H’s on the Ser residue are deshielded in comparison to the typical ppm range observed, consistent with the cyclization due to the proximity to the ester group.

Due to the cyclized and charged structures of tistrellabactins A and B, optimized mass fragmentation was obtained by linearization of tistrellabactins A and B via hydrolysis of the Ser ester linkage (Figures S21–S24). Linearization was achieved with mild conditions by incubation in pH 8 Na_2_HPO_4_ buffer for 48 h, at which point the linear form of tistrellabactin A was observable by UPLC-MS analysis at *m*/*z* 1110.4 [M + H]^+^ and tistrellabactin B at *m*/*z* 1111.4 [M + H]^+^ (Figure S23). Mass fragmentation of both linear and cyclic tistrellabactins A and B is consistent with the NMR-assigned structure. Mass losses of NH_3_ and H_2_O from b and y fragments were observed, as would be expected with highly ionizable side chain amines and hydroxyl groups on Asn, Asp, and Gln residues (Figures 4A, S24).

Tistrellabactins A and B coordinate Fe(III) with the β-OH-Asp, hydroxamate, and *C*-diazeniumdiolate ligands. The UV–vis spectrum of Fe(III)-tistrellabactin A shows an absorption band at 340 nm and a broad shoulder centered at 420 nm. The peak at 340 nm is consistent with a ligand to metal charge transfer band (LMCT) for the α-hydroxycarboxylate ligand, β-OH-Asp, coordinated to Fe(III), while the broad shoulder centered at 420 nm corresponds to the LMCT band for the Fe(III)-hydroxamate group^30,31^ (Figure S25).

### Glutamine Residues in the Tistrellabactins Are Iteratively Loaded via an Unknown NRPS Mechanism

**Figure 5:**
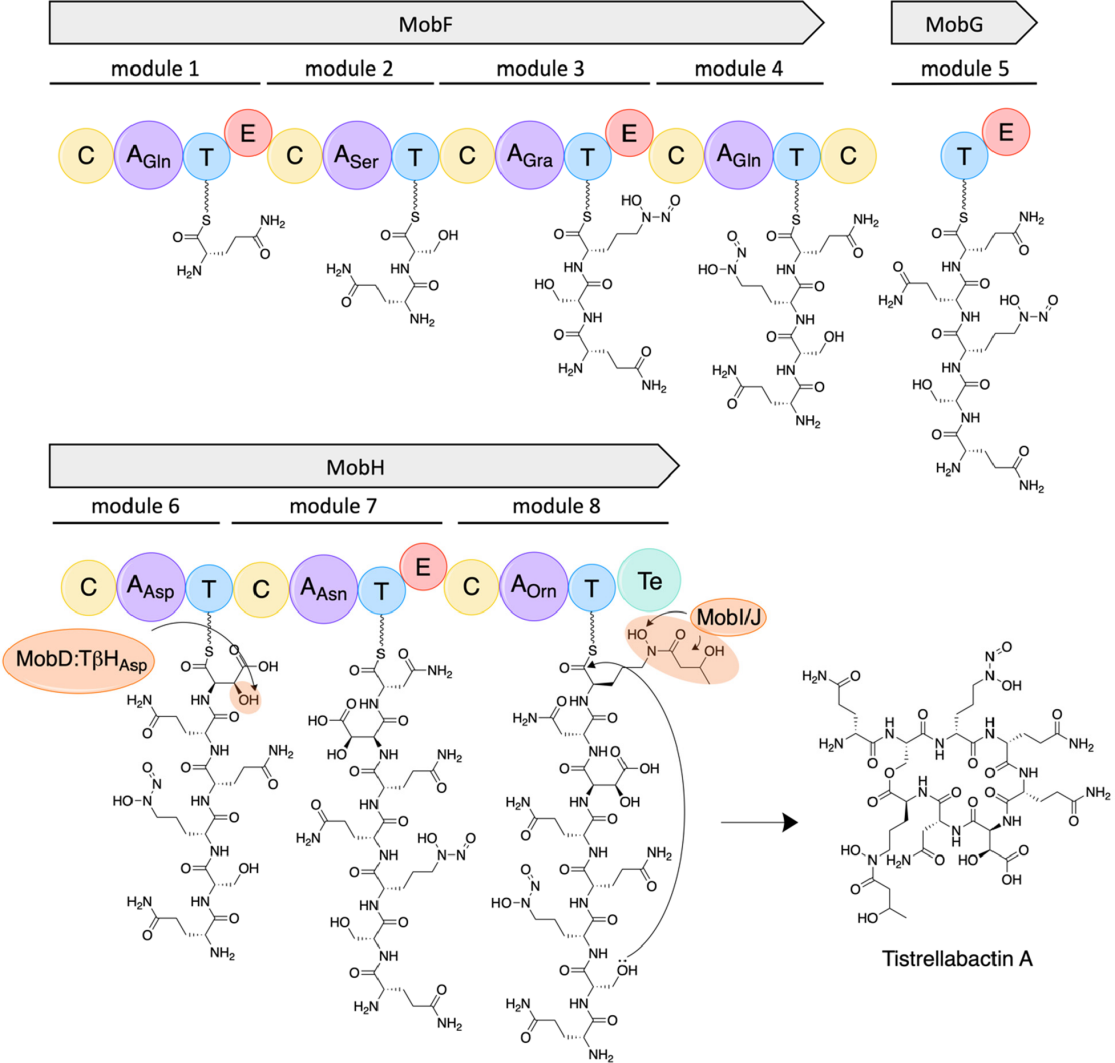
NRPS domain organization and proposed biosynthesis of tistrellabactin A. NRPS modules 4 and 5 iteratively load Gln residues via an unknown mechanism.

The amino acid constituents in the tistrellabactins follow the NRPS collinearity principle in accordance with the identified BGC, except eight amino acids are incorporated in tistrellabactins A and B rather than the predicted seven based on the genomic analysis (Figure 2, Table 1). An extra Gln is observed in NRPS module 4, which is loaded at the end of MobF. This NRPS module may iteratively load an extra Gln, with the possible help of MobG which is missing an A domain. The absolute stereochemistry of the amino acid constituents in the elucidated structures of tistrellabactins A and B was assigned by Marfey’s amino acid analysis. Stereochemical assignments of the amino acid constituents of tistrellabactins A and B agree with the genomic predictions based on the presence or absence of an NRPS epimerization domain, with the exception of the Gln residues, which all are d-configured (Figures 2, 5, S26–S28). The second A domain selective for Gln in MobF is not followed by an E domain; however only d-Gln was detected (Figures S26, S28). Therefore, we propose that MobG may be working with the last module of MobF to epimerize the two Gln residues loaded iteratively at this position (Figure 5). Interestingly, DidA in the didemnin biosynthetic pathway, also identified within the genome of *T. mobilis* KA081020-065, iteratively loads three of four Gln residues in didemnins X and Y.^26^ Didemnins X and Y are formed prior to hydrolysis of the ester bond adjacent to the string of Gln residues to yield the biologically active didemnin B.^26^

**Table 1:** Stachelhaus Specificity Codes of NRPS A Domains in the Tistrellabactin BGC

Stachelhaus code	NRPS Predictor2^36^	characterized residue
DAEYVGSITK	Leu	d-Gln
DVWNVAMVHK	Ser	l-Ser
DVHRTGLVAK	unknown	d-Gra
DAEYAGTITK	unknown	d-Gln
DLTKVGHVGK	Asp	l-*erythro* OH-Asp
DATKIGEVGK	Asn	d-Asn/Asp
DAEDTGRISK	Glu	l-OH-Orn

### ^15^N-Enrichment of Tistrellabactins A and B Shows l-Arg Is the Origin of Gra

^15^N-Enriched tistrellabactins A and B were prepared for ^1^H–^15^N HMBC and HSQC experiments to aid their structural determination (Figures S11, S12, S19, S20). The ^15^N-enriched-tistrellabactins were prepared by culturing *T. mobilis* KA081020-065 in an artificial seawater (ASW) medium using ^15^NH_4_Cl in place of NH_4_Cl as the sole nitrogen source. UPLC-MS analysis of purified ^15^N-enriched tistrellabactins A and B showed both had an isotopic mass of *m*/*z* 1107.4 [M + H]^+^, indicating 15 nitrogens in the structure of tistrellabactin A and 14 nitrogens in tistrellabactin B (Figure S29). Corresponding ionization-induced mass losses of 31 Da are observed for both tistrellabactins A and B, consistent with ionization of ^15^NO instead of ^14^NO.

To determine if the Gra residue in the tistrellabactins originates from l-Arg, given the presence of protein homologues of GrbD and GrbE in the tistrellabactin’s BGC, ^14^N-l-Arg was supplemented into the ^15^N-ASW *T. mobilis* KA081020-065 culture. Purified tistrellabactins A and B grown under these conditions both have an isotopic mass of *m*/*z* 1102.4 [M + H]^+^, indicating five nitrogens in each structure are unenriched. Also notable is the mass ion fragment showing a loss of 30 Da (*m*/*z* 1072 [M + H]^+^). A mass loss of 30 rather than 31 indicates the distal nitrogen on the diazeniumdiolate in Gra is unenriched. As part of the urea cycle,^32^ Arg is converted to Orn, which then may get incorporated before the microbe initiates *de novo* biosynthesis of Orn from ^15^NH_3_. Therefore, the most plausible nitrogens to be unenriched would be the three in the Gra and two in the Orn residue, which matches the MS analysis (Figure S30). Moreover, ^1^H–^15^N HMBC correlations confirm the unlabeled nitrogens are in both Gra and Orn residues and therefore originate from Arg, in agreement with the isotopic enrichment of gramibactin in *P. graminis* DSM 17151 (Figures S30, S31).^14^

### Bioinformatic Analysis of *T. mobilis* KA081020-065 Siderophore Gene Cluster

The putative siderophore BGC identified in the genome of *T. mobilis* KA081020-065 encodes accessory genes consistent with the biosynthesis of three different Fe(III) ligands within the structure. Along with the *C*-diazeniumdiolate biosynthesis genes, tailoring enzymes MobD, MobI, and MobJ would be responsible for the modification of l-Asp and l-Orn to yield β-hydroxyaspartate and the Orn-hydroxamate ligand groups, respectively (Figure 2). A standalone β-hydroxylase enzyme (MobD, TβH_asp_) and an A domain specific for activation of l-Asp in MobH followed by a T_E_ domain (GGDSI motif) directs the stereospecific 3R hydroxylation of the Asp producing the l-*erythro* diastereomer.^33^ Modification of l-Orn by an *N*-acetyltransferase (MobI) and a *N*-monooxygenase (MobJ) yields the hydroxamate group as the third bidentate Fe(III) ligand. The predicted NTN hydrolase, MobM, suggests an N-terminal fatty acyl group may be present during the biosynthesis; however it was not detected in the culture workup.^34^

The core peptidic structure is assembled by the concerted action of three NRPS modules: MobF, MobG, and MobH. The third NRPS module in MobF contains an A domain with specificity code DVHRTGLVAK, matching that of the A domains of Gra in the reported Gra-containing metabolites.^13,15^ NRPS modules minimally consist of a condensation (C), adenylation (A), and thiolation (T) domain; however *mobG* is missing a C and A domain.^35^ At the level of genome mining alone, it is unclear if the C domain at the end of MobF followed by an unannotated gap may be a sequencing error or biosynthetically relevant.

### Tistrellabactins A and B Arise from a Promiscuous Adenylation Domain

NMR assignments, MSMS fragmentation, and ^15^N-isotopic labeling demonstrate that tistrellabactins A and B differ by an Asn and Asp at the analogous position. These results indicate that the A domain in the first NRPS module of MobH can accept either residue. The ability of an A domain to accept both Asn and Asp as a substrate is not entirely surprising given the similarity of the two amino acids in size and polarity. The ratio of the peaks in the HPLC chromatogram of the extracted *T. mobilis* supernatant (215 nm) shows incorporation of Asn is favored over Asp (Figure 3A). Additionally, when *T. mobilis* KA081020-065 cultures are supplemented with l-Asn, the ratio of congeners shifts almost entirely toward tistrellabactin A (Figure S32). Promiscuous NRPS A domains have been observed previously, for example in pacifibactin (Ser/Gly),^29^ xenemetides (Trp/Phe),^37^ moanachelins (Ala/Gly),^38^ and pseudoalterobactin (Asn/Asp) among others.^39,40^

The production of two products from the tistrellabactin BGC may be indicative of NRPS evolution, as functional divergence and enzyme promiscuity are often related.^41^ The promiscuity of the A domain substrate allows for exploration of the fitness landscape without the loss of the siderophore’s activity.^41,42^ In tistrellabactin B, the second Asp is not hydroxylated. If tistrellabactin B was hydroxylated, this conformation may be unfavorable and would be unnecessary for Fe(III) coordination given the presence of three bidentate ligands to provide hexadentate coordination to Fe(III). Assignment of the absolute configuration of the amino acid constituents of the tistrellabactins shows that the Asn/Asp is d-configured, which is in agreement with the NRPS module including an E domain following the A domain that activates the Asn/Asp. The presence of a T_E_ domain with a standalone β-hydroxylase correlates with a hydroxylation event; however the T_E_ also functions to position the amino acid for epimerization.^43^

### The Tistrellabactins Are Photoreactive

**Figure 6:**
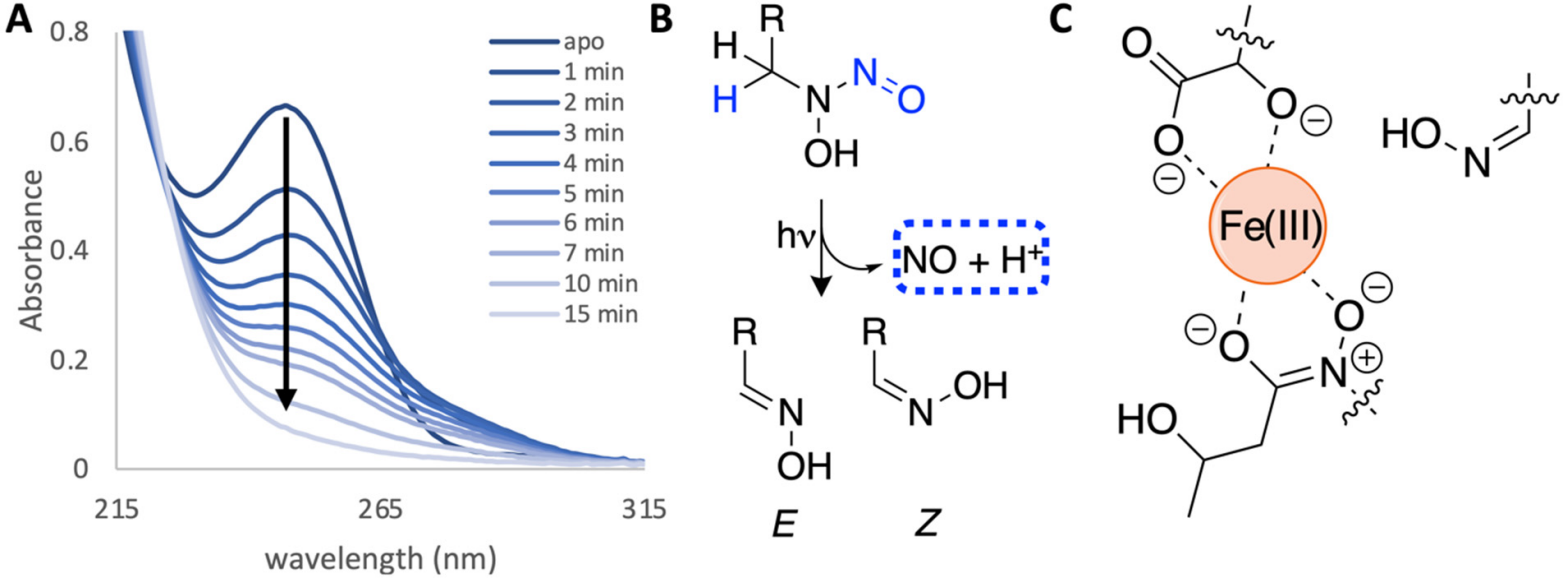
Photoreactivity of apo-tistrellabactin A. (A) The diazeniumdiolate absorbance band at 246 nm disappears with irradiation of UV light. (B) The *C*-diazeniumdiolate is converted to *E*/*Z* oxime isomers. (C) Proposed coordination of the apo-tistrellabactin A photoproduct to Fe(III).

Irradiation of apo-tistrellabactin A with UV light diminishes the intensity of the absorption band at 246 nm as the *C*-diazeniumdiolate undergoes transformation to *E* and *Z* oxime isomers in the same manner as occurs for apo-gramibactin (Figures 6, S33–S35).^14^ UPLC-ESIMS analysis of the apo-tistrellabactin A photoproduct shows a new mass of *m*/*z* 1061.4 [M + H]^+^ and complete loss of the apo-tistrellabactin A *m*/*z* 1092.4 [M + H]^+^ species (Figure S33). Also present is a mass showing Fe(III) coordinated to the photoproduct with mass *m*/*z* 1114.3 [M – 2H + Fe]^+^, demonstrating tistrellabactin A retains its ability to coordinate Fe(III) with the remaining hydroxamate and β-OH-Asp ligands (Figures 6C, S33).

Actively growing cells from a culture of *T. mobilis* with a positive chrome-azurol-S assay (CAS) response, indicating siderophore production, were also photolyzed in UV light. After 1 h of irradiation, the cells were pelleted and the resulting supernatant was analyzed by UPLC-MS (Figure S35). The resulting sample shows a mass loss of 31 from the peaks corresponding to tistrellabactins A and B, consistent with the photoproduct observed for the purified and concentrated sample. In comparison, a nonirradiated aliquot from the same culture shows no change to the apo-tistrellabactins and no trace of their respective photoproducts (Figure S35). These results demonstrate the biological relevance of the photoreactivity of these siderophores.

Less is understood about the photoreactivity of the Fe(III)-bound diazeniumdiolate complex. It is well established that the Fe(III)-β-OH-Asp will undergo a radical decarboxylation reaction when irradiated, potentially playing an important role of iron cycling in the marine ecosystem.^44^ When Fe(III)-tistrellabactin A (*m*/*z* 1145.4 [M – 2H + Fe]^+^) is photolyzed selectively with 254 nm UV light (Hg(Ar) Oriel No. 6035), the intensity of the LMCT band for the α-hydroxycarboxylate and the hydroxamate to Fe(III) both decrease, while a band at 270 nm evolves and increases steadily with a bathochromic shift to 277 nm over 13.5 h of irradiation (Figure S36). Aliquots taken along the photolysis time course were analyzed by MS and show the decrease of the Fe(III)-tistrellabactin protonated molecule and the appearance of several new protonated molecules. The β-OH-Asp and the graminine residues undergo photoreactions, as evinced by the presence of doubly charged protonated molecules at *m*/*z* 550.1 and 557.6 [M + 2H]^2+^, consistent with the decarboxylation of the β-OH-Asp (46 Da) or transformation to *E*/*Z* oxime isomers from the *C*-diazeniumdiolate group (31 Da), respectively, while retaining coordination to Fe(III). At later time points in the photolysis the ion count of the protonated molecule at *m*/*z* 508.2 [M + 2H]^2+^ increases further, consistent with both the graminine and β-OH-Asp ligands having undergone photoreactions with the loss of the ability to coordinate Fe(III) (Figure S37).

## Conclusions

The marine-derived microbe *Tistrella mobilis*KA081020-065 produces not only the bioactive didemnins,^26^ but also, as presented here, the two depsipeptides tistrellabactins A and B, with rare biosynthetic features. The BGC of the tistrellabactins was identified in a previous study,^26^ although the expression of these metabolites was not observed when cultured in iron-rich media, highlighting the importance of mimicking certain environmental cues for laboratory culturing. To induce siderophore production, *T. mobilis* KA081020-065 was cultured under Fe-poor conditions, yielding tistrellabactins A and B.

The tistrellabactins are mixed ligand siderophores composed of three Fe(III) ligand types: a *C*-diazeniumdiolate, an α-hydroxycarboxylate, and a hydroxamate. This report is the fourth account of a *C*-diazeniumdiolate siderophore with Gra, but the first to be produced by a marine-derived microbe. In the process of characterizing the structure, several unexpected features were uncovered, which could not have been predicted *a priori* by genome mining, including an iterative addition of Gln, a promiscuous A domain, the incorporation of a 3-hydroxybutyric acid group, and a 22-membered macrolactone ring. As a result of the promiscuous NRPS adenylation domain, two siderophores arise from a single BGC, differing only by a d-Asn in tistrellabactin A and a d-Asp in tistrellabactin B. The identification of promiscuous enzymes, including A domains, is of interest, as they have been leveraged for bioengineering efforts.^45,46^ A hydroxylated ornithine appended with a 3-hydroxybutyric acid group was elucidated, providing a hydroxamate-type ligand for bidentate Fe(III) coordination. The third Fe(III) ligand was identified as the *C*-diazeniumdiolate, and isotopic feeding study results further substantiate the origin of Gra is indeed Arg.^14^ The mixed-ligand tistrellabactins are atypical siderophores, with three different Fe(III)-binding ligand types.

The most intriguing feature of the tistrellabactins is their photoreactivity, given the environment from which *T. mobilis* was isolated. The tistrellabactins lose an equivalent of NO and H^+^ from the Gra residue to give an *E*/*Z* oxime isomer photoproduct following the same photoreactivity as the siderophore gramibactin.^14^ In contrast to the photoproduct of apo-gramibactin, the apo-tistrellabactins are still able to maintain their ability to coordinate Fe(III) with the presence of two bidentate ligands remaining postphotolysis as opposed to one in the photoproduct of apo-gramibactin.

Discovery of the tistrellabactins adds to the emerging family of *C*-diazeniumdiolate graminine-containing siderophores.^13,15^ Unlike the previously reported siderophores in this family, which are all isolated from rhizospheric microbes,^13,15^*T. mobilis* KA081020-065 originates from the Red Sea, establishing a much wider distribution of this recently identified siderophore type. The BGC encoding the tistrellabactins was identified on a plasmid; consequently the genes may have been acquired via horizontal gene transfer. This gene cluster’s presence on a plasmid rather than within a chromosome is also appealing for potential bioengineering of this BGC.^47^ Natural products containing N–N bonds are often found to have biological activity;^1^ therefore the understanding of how those bonds are formed can open up doors within synthetic biology to take advantage of this functionality. We propose l-Arg is oxidatively rearranged to yield Gra, utilizing at least the two protein homologues of GrbE and GrbD, which are putatively assigned as an Arg hydroxylase and a nonheme iron enzyme capable of oxidative rearrangement to form the N–N bond. Further investigations will deduce whether the putative arginine hydroxylase fused to an NRPS domain in the tistrellabactins BGC (MobF, Figure 2) in contrast to the standalone enzyme GrbE in the BGC of gramibactin is of biosynthetic relevance. Furthering our knowledge of these enzymes will also strengthen genome mining tools, which in turn may help to identify the potential incorporation of other nonproteinogenic amino acids with a diazeniumdiolate group, such as l-alanosine,^6,9^ into peptidic structures and continue uncovering the expanding diversity of siderophores.

## Experimental Section

### General Experimental Procedures

UV–visible spectra were obtained on an Agilent Technologies Cary 300 UV–vis spectrometer. NMR spectroscopy was carried out at 25 °C on a Bruker 500 MHz spectrometer equipped with a Prodigy cold probe (^1^H, ^13^C, ^1^H–^13^C multiplicity edited HSQC, ^1^H–^1^H COSY, ^1^H–^13^C HMBC, ^1^H–^15^N HMBC, ^1^H–^15^N HSQC, TOCSY, NOESY). NMR spectra for characterization were collected in DMSO-*d*_6_, and spectra of the photoproduct were collected in 50 mM Na_2_HPO_4_-buffered D_2_O (pD 8.0). Spectra were indirectly referenced by the residual solvent peak or ^2^H lock. MS analysis was carried out on a Waters Xevo G2-XS QTOF with positive mode electrospray ionization coupled to an AQUITY UPLC-H-Class system with a Waters BEH C18 column. Samples were run with a linear gradient of 0% to 100% acetonitrile (0.1% formic acid) in ddH_2_O (0.1% formic acid) over 10 min. IR data were collected on a Bruker Alpha FTIR spectrophotometer. CD data were collected on a JASCO J-1500 CD spectrometer on tistrellabactin A (71 μM) and tistrellabactin B (52 μM) in Na_2_HPO_4_ (pH 8). CD data was referenced to the buffer and collected with a scan rate of 20 nm/min, DIT 8 s bandwidth of 1 nm, and data pitch of 0.5 nm. Deionized water was dispensed from a Milli-Q IQ 7000 water purification system (Resistivity 18.2 MΩ). All glassware was acid-washed with 4 M HCl and subsequently rinsed with Milli-Q water.

### Culturing *T. mobilis* KA081020-065 and Siderophore Isolation

The strain *Tistrella mobilis* KA081020-065 was obtained from Scripps Institution of Oceanography, University of California, San Diego. For optimal production of tistrellabactins A and B, *T. mobilis* KA081020-065 was cultured in a modified ASW medium composed of 62 mM sodium succinate, 19 mM NH_4_Cl, 50 mM MgSO_4_·7H_2_O, 10 mM KCl, 10 mM CaCl_2_·2H_2_O, 6 mM glycerol phosphate, 0.28 M NaCl, 41 mM glycerol, and 45.4 mM sodium pyruvate in 1 L of Milli-Q water with the pH adjusted to 7. The medium was optimized for the growth of the tistrellabactins, which increased yields from 0.1 mg/L to 5–8 mg/L. Seed cultures were inoculated in Difco 2216 marine broth with single colonies of *T. mobilis* KA081020-065 grown on 2216 agar and grown for at least 24 h at 28 °C. Two dense 5 mL seed cultures were used for inoculation, as it was found this strain required higher amounts of inoculum than normal. Following sterilization, the medium was amended with 2 mM Steri-filtered NaHCO_3_. Cultures were monitored by OD_600_, and the culture supernatant was harvested at late log/early stationary phase with a positive Fe(III)-CAS response.^48^

Culture supernatants were obtained by centrifugation at 6000 rpm for 30 min at 4 °C (SLA-3000 rotor, Thermo Scientific). To extract the tistrellabactins, the culture supernatant was decanted and shaken with 100 g of Amberlite XAD-4 resin. The XAD-4 resin was prepared by washing with methanol and then equilibrating with Milli-Q water. The resin and supernatant were allowed to equilibrate for 4 h at 120 rpm. The resin was filtered from the supernatant and washed with 0.5 L of Milli-Q water. The adsorbed organics were eluted with 40% aqueous methanol. The eluent was concentrated under vacuum and stored at 4 °C. Tistrellabactins A and B were purified from the eluent by semipreparative HPLC on a YMC 20 × 250 mm C18-AQ column, with a linear gradient of 10–55% methanol (0.05% trifluoroacetic acid) over 45 min, yielding pure tistrellabactins A (8 mg) and B (5 mg) from 1 L of culture.

### Isotopic Labeling of Tistrellabactins A and B

The ASW medium used for culture growth only contains one nitrogen source. To isotopically label the full structures of tistrellabactins A and B, ^15^NH_4_Cl was used in place of NH_4_Cl. An additional experiment was completed with 10 mM ^14^N-Arg added to the ^15^N-enriched culture medium to follow potential utilization of l-Arg in the biosynthesis.

### Marfey’s Amino Acid Analysis

Aliquots (1 mg in Milli-Q water) of tistrellabactin A and tistrellabactin B were combined with 12 M HCl or with 57% HI for final concentrations of 6 M HCl or 45% HI. The acidified solution was transferred to a glass ampoule, blanketed with Ar(g), and sealed under flame. The ampoule was heated at 108 °C for 21 h. After heating, the ampoule was broken, and the crude solution was evaporated and redissolved in 0.7 mL of Milli-Q water five times to ensure acid was removed from the hydrolysate. A 100 mL aliquot of the hydrolysate was reacted with Marfey’s reagent following standard conditions.^49^ Amino acid standards (dl-Glu, l-Glu, l-Orn, dl-Asp, l-Ser) were derivatized under the same conditions.

The FDAA-hydrolysate was analyzed by UPLC-MS and RP-HPLC (250 × 4.6 mm YMC C18-AQ column). HPLC samples were monitored at 340 nm using a linear gradient of 15% to 50% acetonitrile in 50 mM triethylamine phosphate (pH 3.0) over 50 min. The hydrolysate and standards were also analyzed by UPLC-MS (15% to 50% acetonitrile with 0.1% formic acid in Milli-Q water) to identify the graminine and l-*erythro* hydroxyaspartic acid, in positive ion mode.

### Apo-tistrellabactin Photolysis Conditions

Photolysis of apo-tistrellabactin A was carried out in a quartz NMR tube. An Oriel Instruments Hg(Ar) (No. 6035) pen lamp was used as the UV source. Samples were dissolved in aqueous buffer (50 mM Na_2_HPO_4_ prepared in D_2_O (99.9% purity)) with the pD adjusted to 8.0. For the Fe(III)-tistrellabactin A photolysis, an Edmund Optics filter (253.7 nm, 25.00 mm diameter, 40.00 nm full width at half-maximum) was used to selectively irradiate at 253.7 nm.

### Tistrellabactin Iron(III) Titration

Apo-tistrellabactin A (85 μM, citrate phosphate, pH 8) was titrated with a standardized Fe(III) stock solution (FeCl_3_ in 25 mM HCl, 2.58 ± 0.04 mM) followed by UV–vis spectroscopy. The solution was equilibrated for 30 min after each addition of Fe(III). The Fe(III) stock was standardized spectrophotometrically with 1–10 phenanthroline (510 nm, ε 11,100 M^–1^ cm^–1^) and hydroxylamine as the reducing agent. Titration of apo-tistrellabactin A with Fe(III) yields extinction coefficients calculated at the 1:1 Fe(III)-siderophore breakpoint: 1479 ± 173 M^–1^ cm^–1^ at 420 nm, 2959 ± 300 M^–1^ cm^–1^ at 340 nm, and 9013 ± 774 M^–1^ cm^–1^ at 246 nm over three trials (Figure S25).

### Fe(III)-tistrellabactin A Photolysis

Photolysis of Fe(III)-tistrellabactin A was carried out in a quartz cuvette with a 75 mm stir bar. An Oriel Instrument Hg(Ar) (No. 6035) pen lamp was used as the UV source equipped with a bandpass filter (253.7 nm, Edmund Optics).

#### Tistrellabactin A:

white solid; UV–vis, λ_max_ (log ε) 247 nm (3.95) (Na_2_HPO_4_, pH 8.0); ECD (71 μM, 5 mM Na_2_HPO_4_, pH 8) λ_max_ (Δε) 204 nm (+16.53), Figure S38; IR, 1655 cm^–1^ (s, C=O), 1423 cm^–1^ (w, N–O), 1265 cm^–1^ (w, N–O), Figure S39; ^1^H, ^13^C, and 2D NMR data, Table S2; HRESIMS *m*/*z* 1114.4377 [M + Na]^+^ (calcd for C_40_H_65_N_15_O_21_Na, 1114.4391).

#### Tistrellabactin B

white solid (Na_2_HPO_4_, pH 8.0); ECD (52 μM, 5 mM Na_2_HPO_4_, pH 8) λ_max_ (Δε) 204 nm (+17.65), Figure S38; IR, 1654 cm^–1^ (s, C=O), 1447 cm^–1^ (w, N–O), 1262 cm^–1^ (w, N–O), Figure S39; ^1^H, ^13^C, and 2D NMR data, Table S5; HRESIMS *m*/*z* 1093.4380 [M + H]^+^ (calcd for C_40_H_64_N_14_O_22_, 1093.4398).

## Supporting Information Available

ESIMS/ESIMS/MS, NMR, UV–vis from titration, HPLC chromatograms from amino acid analysis, CD, and IR (PDF)
