# Supplementary material for: Tistrellabactins A and B Are Photoreactive C-Diazeniumdiolate Siderophores from the Marine-Derived Strain Tistrella mobilis KA081020-065
Source: J Nat Prod. 2023 Jun 21;86(7):1770–8. doi: 10.1021/acs.jnatprod.3c00230 (PMC10391617; doi:10.1021/acs.jnatprod.3c00230)
Supplement: Supplementary file 1 — np3c00230_si_001.pdf [file np3c00230_si_001.pdf]

Tistrellabactins A and B are photoreactive *C*-diazeniumdiolate siderophores from marine-derived strain *Tistrella mobilis* KA081020-065

Christina Makris, Jamie K. Leckrone, Alison Butler*

Department of Chemistry & Biochemistry, University of California, Santa Barbara, CA 93106-9510

* To whom correspondence may be addressed. butler@chem.ucsb.edu

Figure S1. Mass spectra of tistrellabactins A (top) and B (bottom) 3
Figure S2. High resolution MS spectrum of tistrellabactin A 4
Figure S3. High resolution MS spectrum of tistrellabactin B 4
Figure S4. Annotated ^{1}H NMR spectrum of tistrellabactin A 5
Figure S5. ^{13}C NMR spectrum of tistrellabactin A 6
Figure S6. Multiplicity edited ^{1}H-^{13}C HSQC NMR spectrum of tistrellabactin A 7
Figure S7. ^{1}H-^{13}C HMBC NMR spectrum of tistrellabactin A 8
Figure S8. ^{1}H-^{1}H COSY NMR spectrum of tistrellabactin A 9
Figure S9. TOCSY NMR spectrum of tistrellabactin A 10
Figure S10. NOESY NMR spectrum of tistrellabactin A 11
Figure S11. ^{1}H-^{15}N HMBC NMR spectrum of tistrellabactin A 12
Figure S12. ^{1}H-^{15}N HSQC NMR spectrum of tistrellabactin A 13
Figure S13. ^{1}H NMR spectrum of tistrellabactin B 14
Figure S14. ^{13}C NMR spectrum of tistrellabactin B 15
Figure S15. Multiplicity edited ^{1}H-^{13}C HSQC NMR spectrum of tistrellabactin B 16
Figure S16. ^{1}H-^{13}C HMBC NMR spectrum of tistrellabactin B 17
Figure S17. ^{1}H-^{1}H COSY NMR spectrum of tistrellabactin B 18
Figure S18. TOCSY NMR spectrum of tistrellabactin B 19
Figure S19. ^{1}H-^{15}N HMBC NMR spectrum of tistrellabactin B 20
Figure S20. ^{1}H-^{15}N HSQC NMR spectrum of tistrellabactin B 21
Figure S21. MSMS fragmentation of parent ion *m/z* 1062 22
Figure S22. MSMS fragmentation of parent ion *m/z* 1063 23
Figure S23. Tistrellabactin A incubated in Na_2HPO_4 buffer (pH 8) cleaves the ester bond 24
Figure S24. MSMS fragmentation of linear tistrellabactin A 25
Figure S25. Fe(III) coordination to tistrellabactin A 26
Figure S26. Marfey's amino acid analysis of tistrellabactin A by HPLC 27
Figure S27. Marfey's amino acid analysis of tistrellabactin A FDAA-hydrolysate shows β-OH-Asp has L-*erythro* configuration 28
Figure S28. Marfey's amino acid analysis of tistrellabactin B by HPLC 29
Figure S29. UPLC-MS analysis of *Tistrella mobilis* KA081020-065 grown with $^{15}NH_4Cl$ as the sole nitrogen source 30
Figure S30. UPLC-MS analysis of *Tistrella mobilis* KA081020-065 grown with $^{15}NH_4Cl$ and ^{14}N-L-Arg as the nitrogen sources 31

Figure S31. ^{1}H-^{15}N HMBC NMR spectrum of ^{15}N-enriched tistrellabactin A isolated from *T. mobilis* grown with $^{15}NHCl_4$ and ^{14}N-L-Arg as the only two nitrogen sources 32
Figure S32. *T. mobilis* supplemented with 20 mM L-Asn 33
Figure S33. Mass spectrum of tistrellabactin A photoproduct (*m/z* 1061.4) 33
Figure S34. NMR analysis of apo-tistrellabactin A photoproduct 34
Figure S35. Mass spectra of aliquots removed from an actively growing culture of *T. mobilis* 35
Figure S36. Photolysis of Fe(III)-tistrellabactin A 36
Figure S37. Mass spectra of aliquots from Fe(III)-tistrellabactin A (TistA), photolyzed selectively at 254 nm 37
Figure S38. Circular dichroism spectrum of tistrellabactins A (TistA) and tistrellabactin B (TistB) 38
Figure S39. IR spectra of tistrellabactin A (top) and tistrellabactin B (bottom). 39

Table S1. Gene cluster annotation for the tistrellabactins isolated from *T. mobilis* 40
Table S2. Tistrellabactin A NMR chemical resonances and correlations 41
Table S3. Tistrellabactin A TOCSY 2D NMR correlations 42
Table S4. Tistrellabactin A ^{1}H-^{15}N HMBC and HSQC data 42
Table S5. Tistrellabactin B NMR ^{1}H, ^{13}C, ^{15}N chemical resonances 44

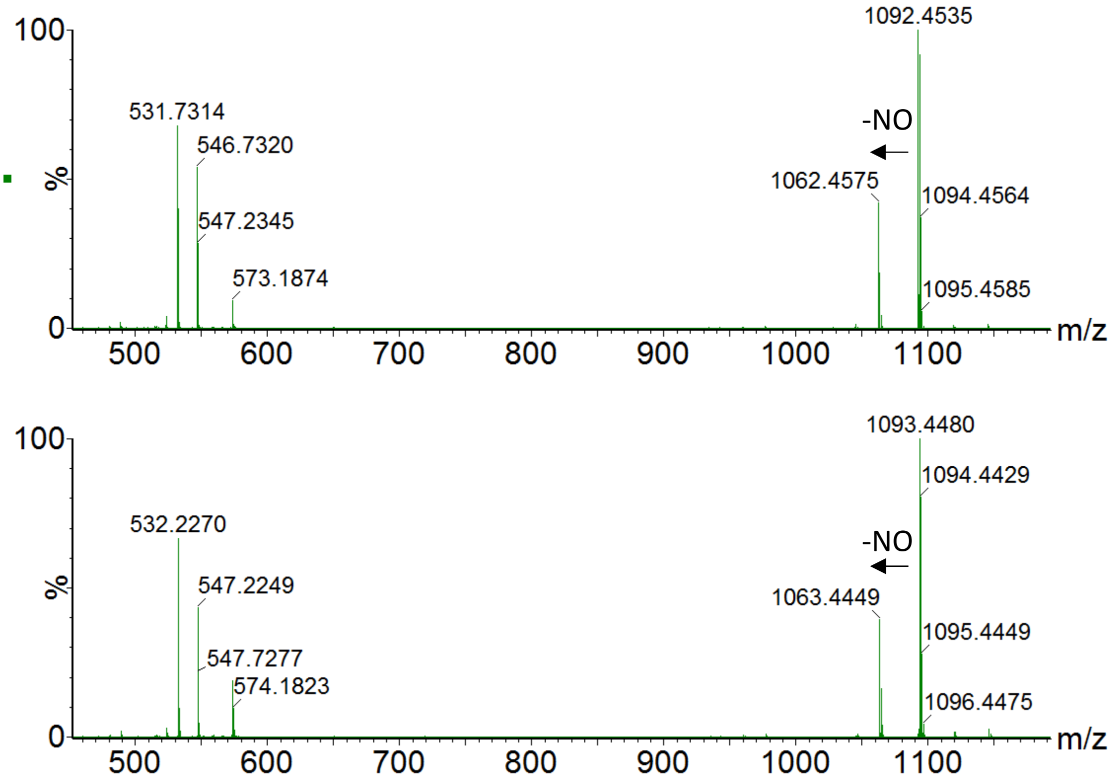

Figure S1. Mass spectra of tistrellabactins A (top) and B (bottom) showing -30 (-NO) ion fragments and doubly charged masses of apo-tistrellabactins A and B -30, apo-tistrellabactins A and B, and Fe(III)-tistrellabactin A and B species.

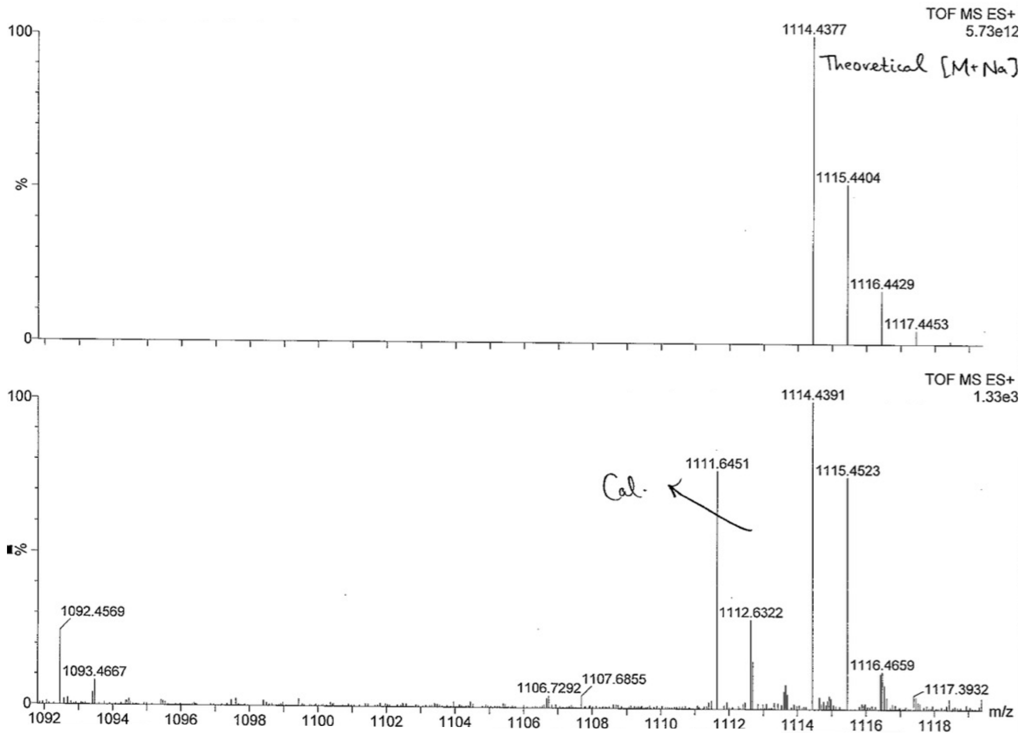

Figure S2. High resolution MS spectrum of tistrellabactin A, *m/z* 1114.4391 $[M+Na]^{+}$.

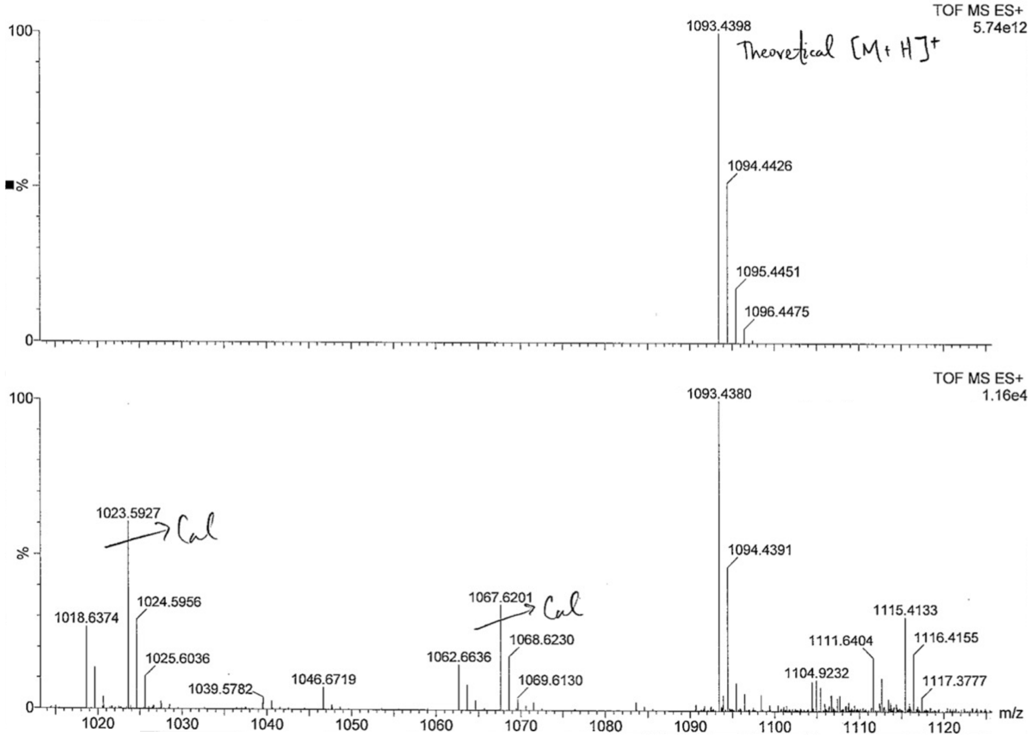

Figure S3. High resolution MS spectrum of tistrellabactin B, *m/z* 1093.4380 $[M+H]^{+}$.

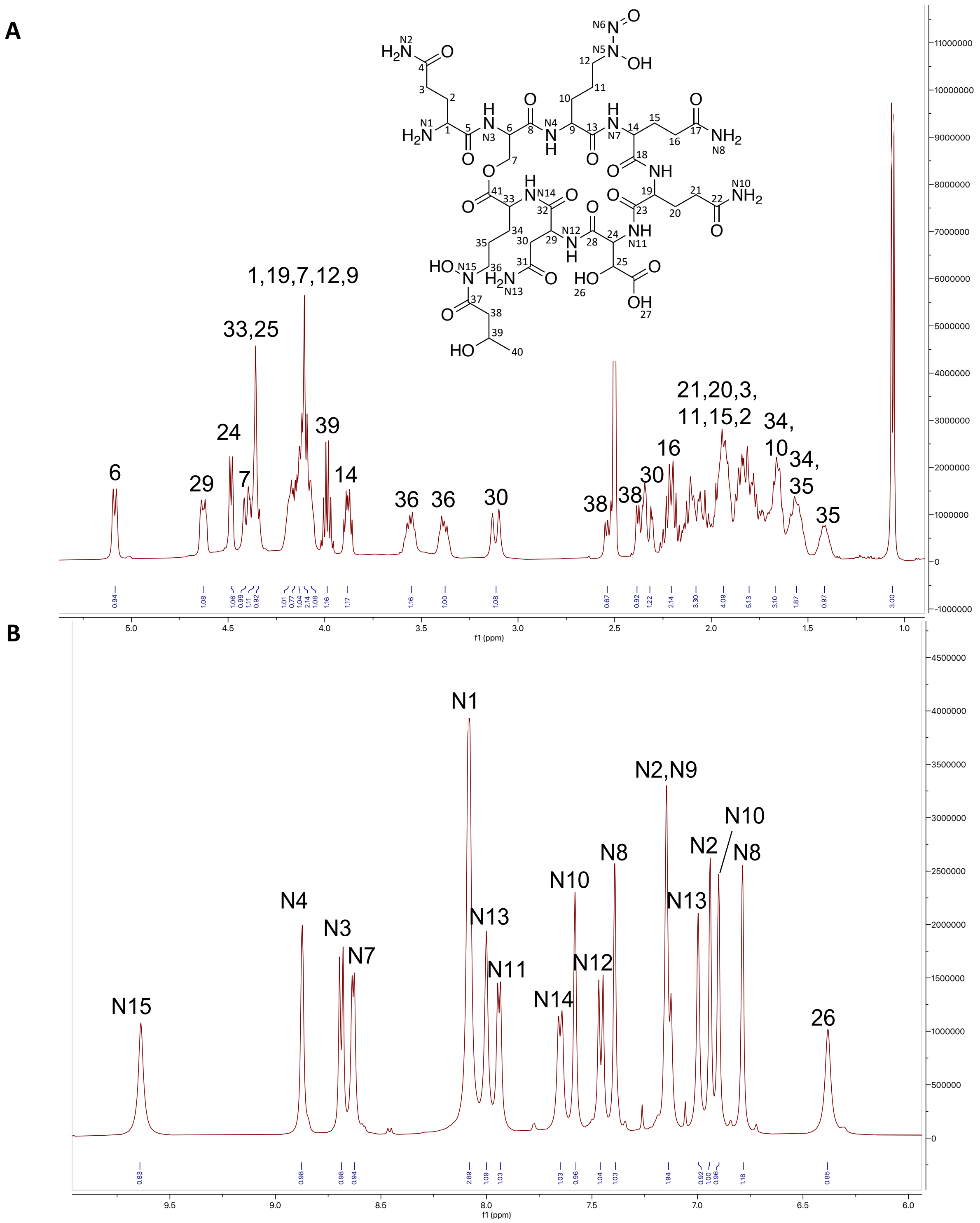

Figure S4. Annotated ^{1}H NMR spectrum of tistrellabactin A, in DMSO-d_6, collected on Bruker 500 MHz spectrometer. (A) 0-5 ppm and (B) 6-10 ppm.

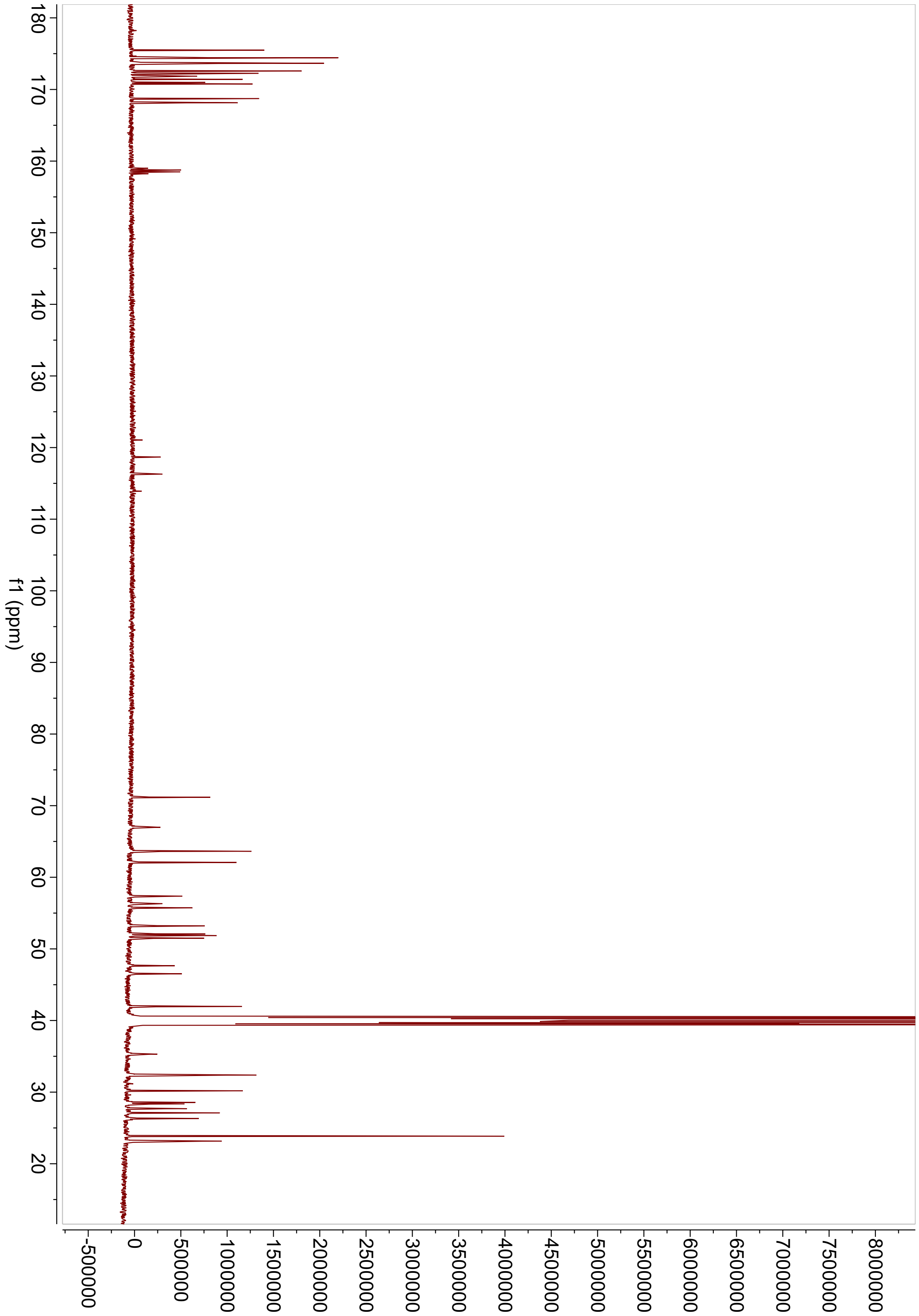

Figure S5. ^{13}C NMR spectrum of tistrellabactin A in DMSO-d_6, collected on Bruker 500 MHz spectrometer. Residual trifluoroacetic acid (used in HPLC purification) ^{13}C shifts are observed due to coordination to the positively charged siderophore.

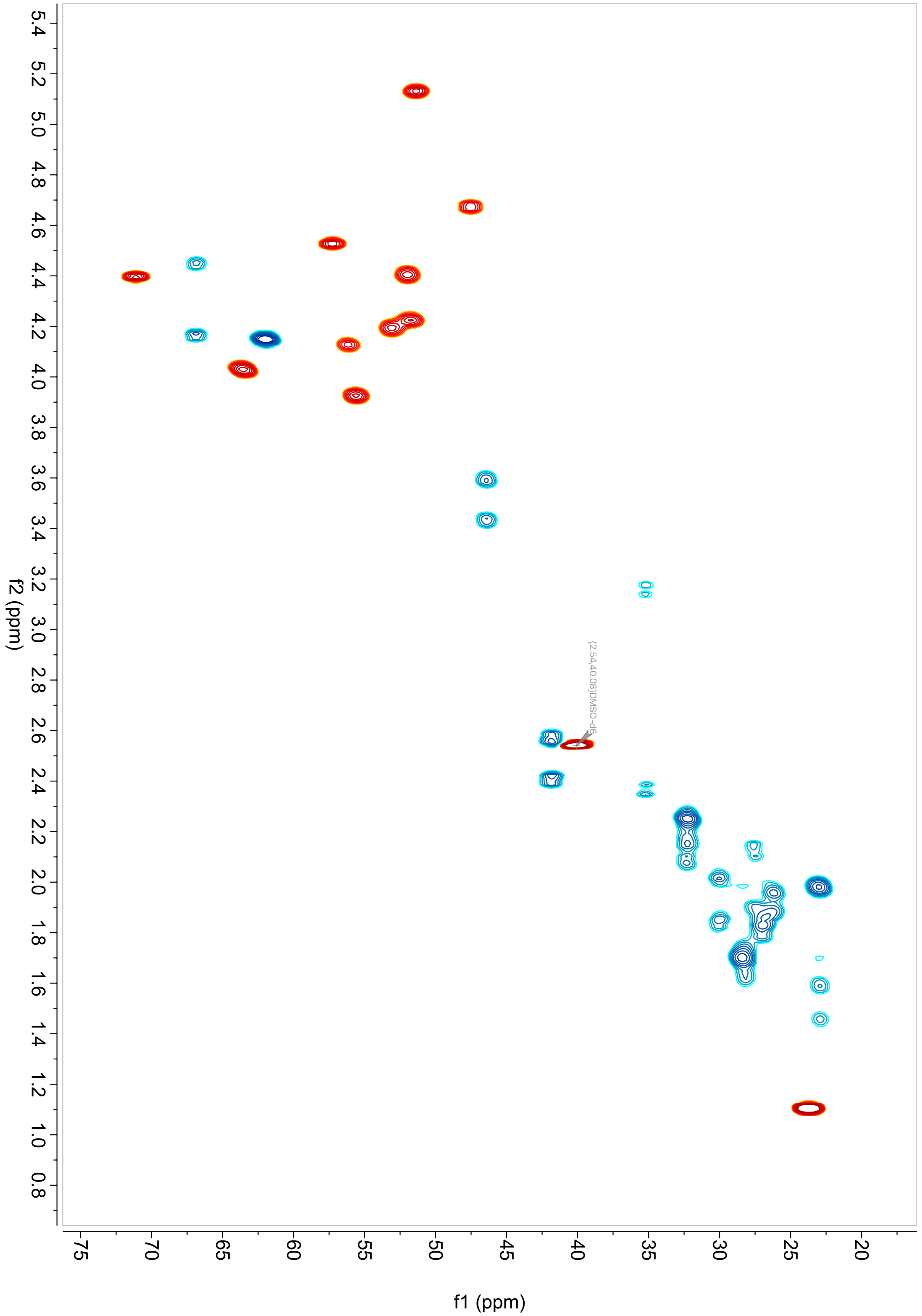

Figure S6. Multiplicity edited ^{1}H-^{13}C HSQC NMR spectrum of tistrellabactin A in DMSO-*d*$_6$, collected on Bruker 500 MHz spectrometer.

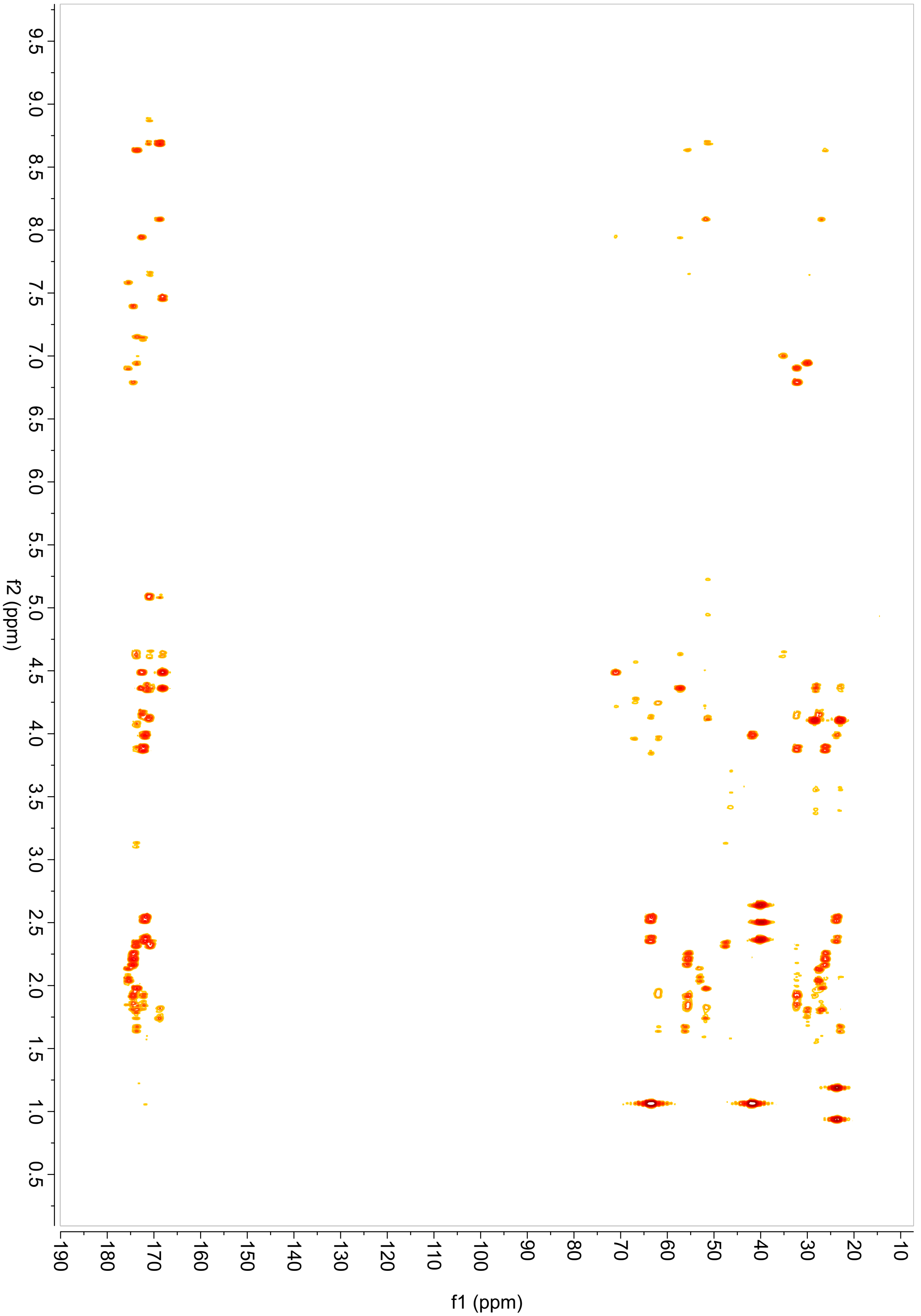

Figure S7. ^{1}H-^{13}C HMBC NMR spectrum of tistrellabactin A in DMSO-d_6, collected on Bruker 500 MHz spectrometer.

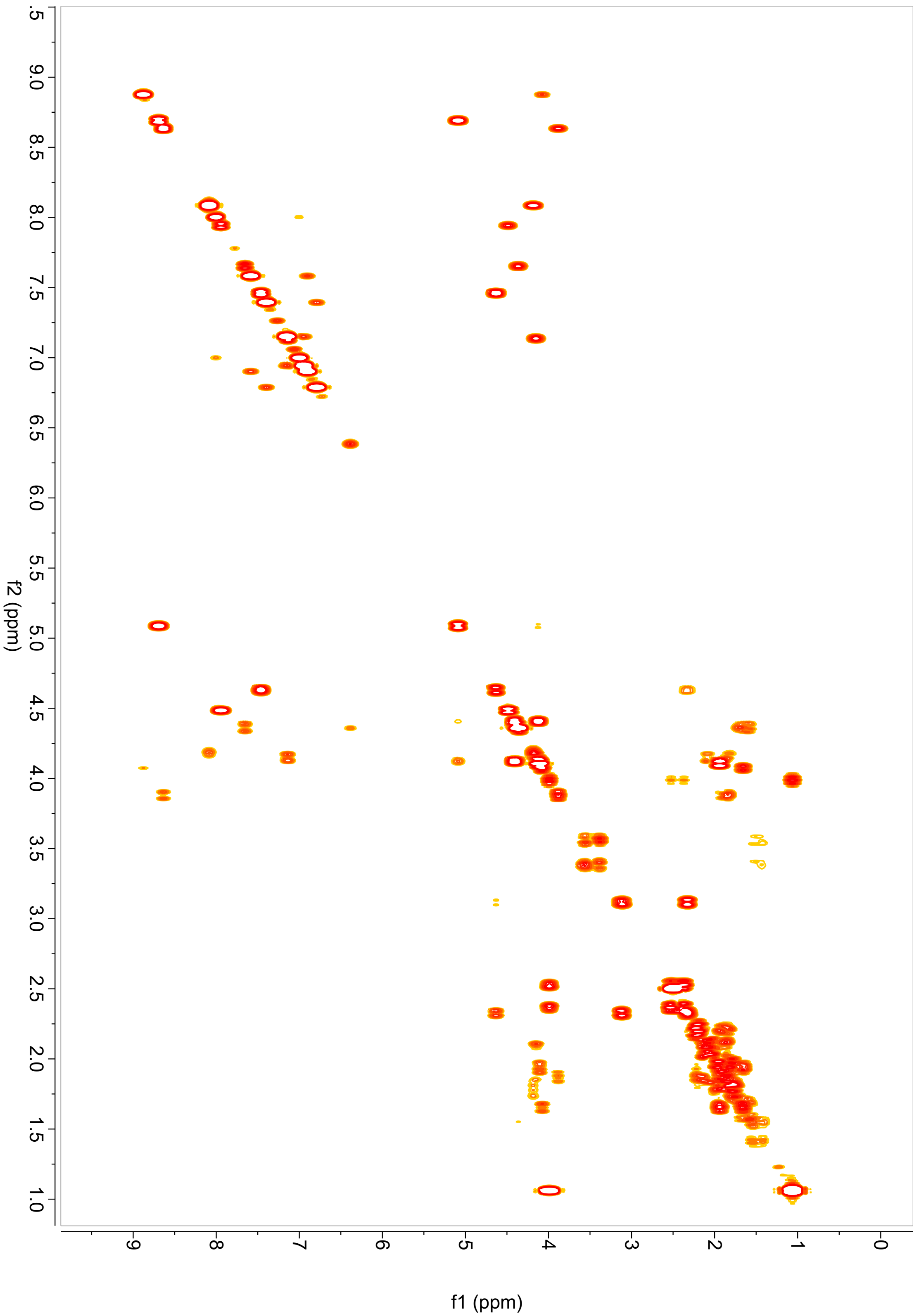

Figure S8. ^{1}H-^{1}H COSY NMR spectrum of tistrellabactin A in DMSO-d_6, collected on Bruker 500 MHz spectrometer.

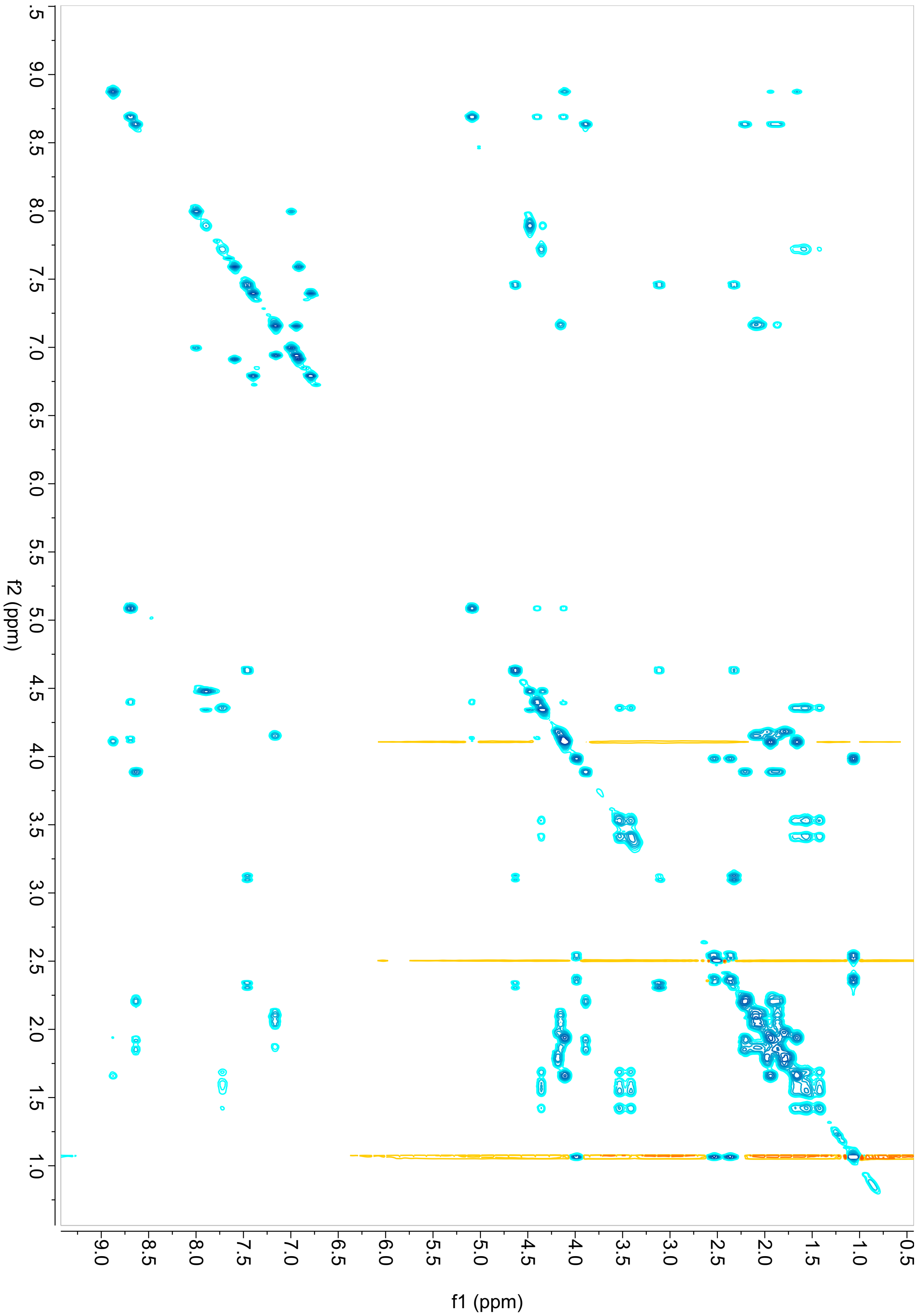

Figure S9. TOCSY NMR spectrum of tistrellabactin A in DMSO-d_6, collected on Bruker 500 MHz spectrometer.

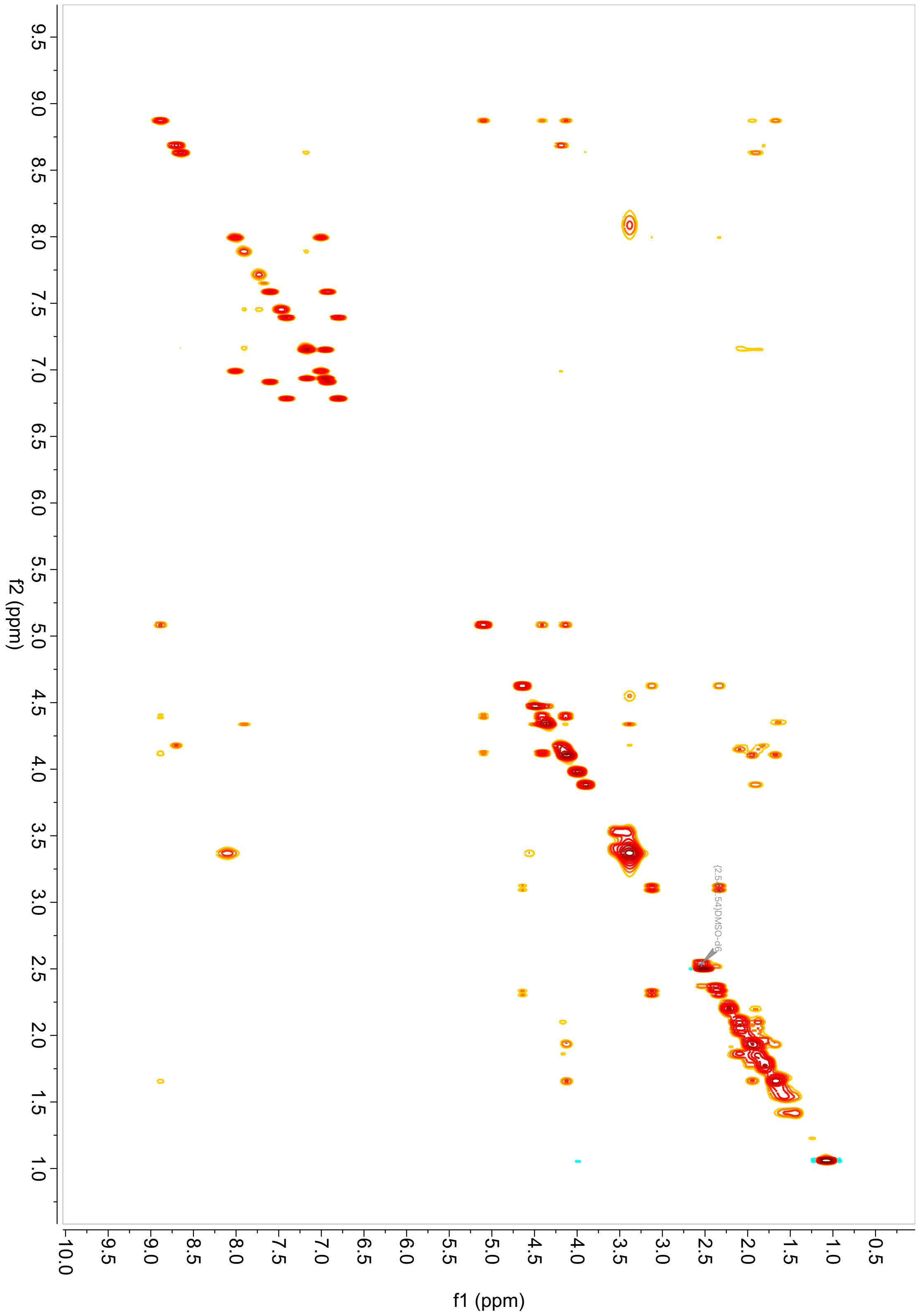

Figure S10. NOESY NMR spectrum of tistrellabactin A in DMSO-d_6, collected on Bruker 500 MHz spectrometer.

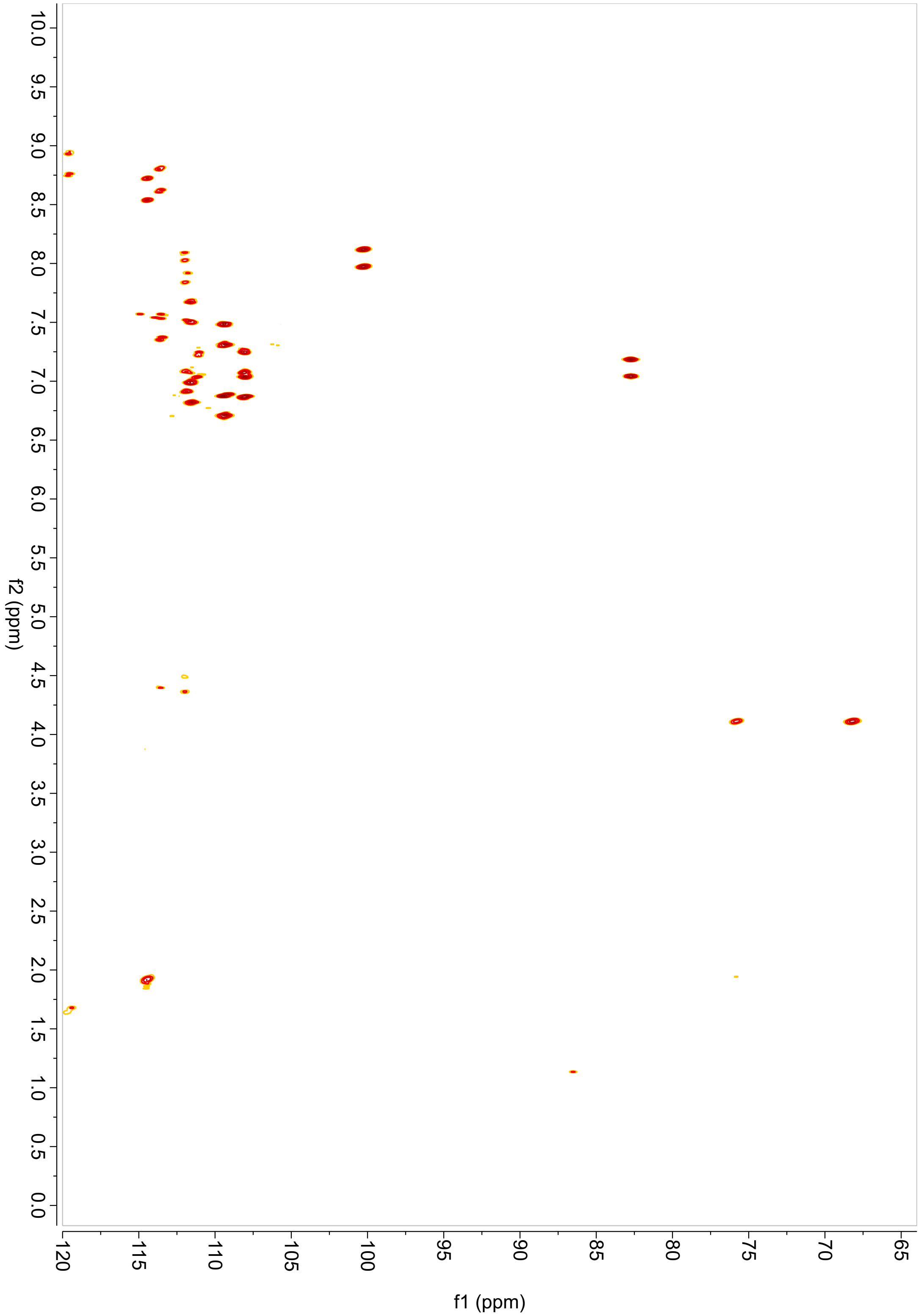

Figure S11. ^1H-^{15}N HMBC NMR spectrum of tistrellabactin A in DMSO-d_6, collected on Bruker 500 MHz spectrometer with ^{15}N spectral window set from 60-120 ppm.

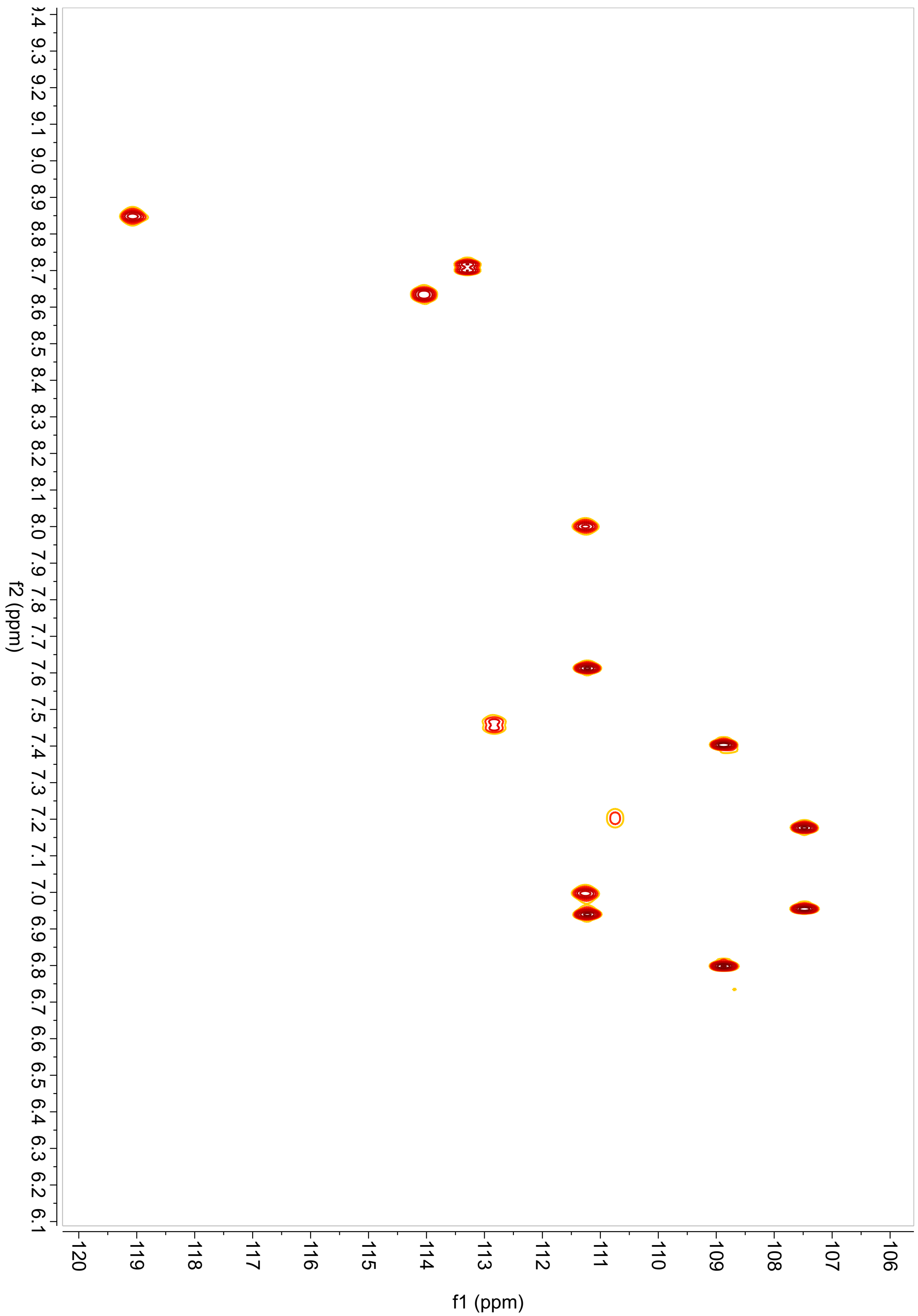

Figure S12. ^{1}H-^{15}N HSQC NMR spectrum of tistrellabactin A in DMSO-d_6, collected on Bruker 500 MHz spectrometer.

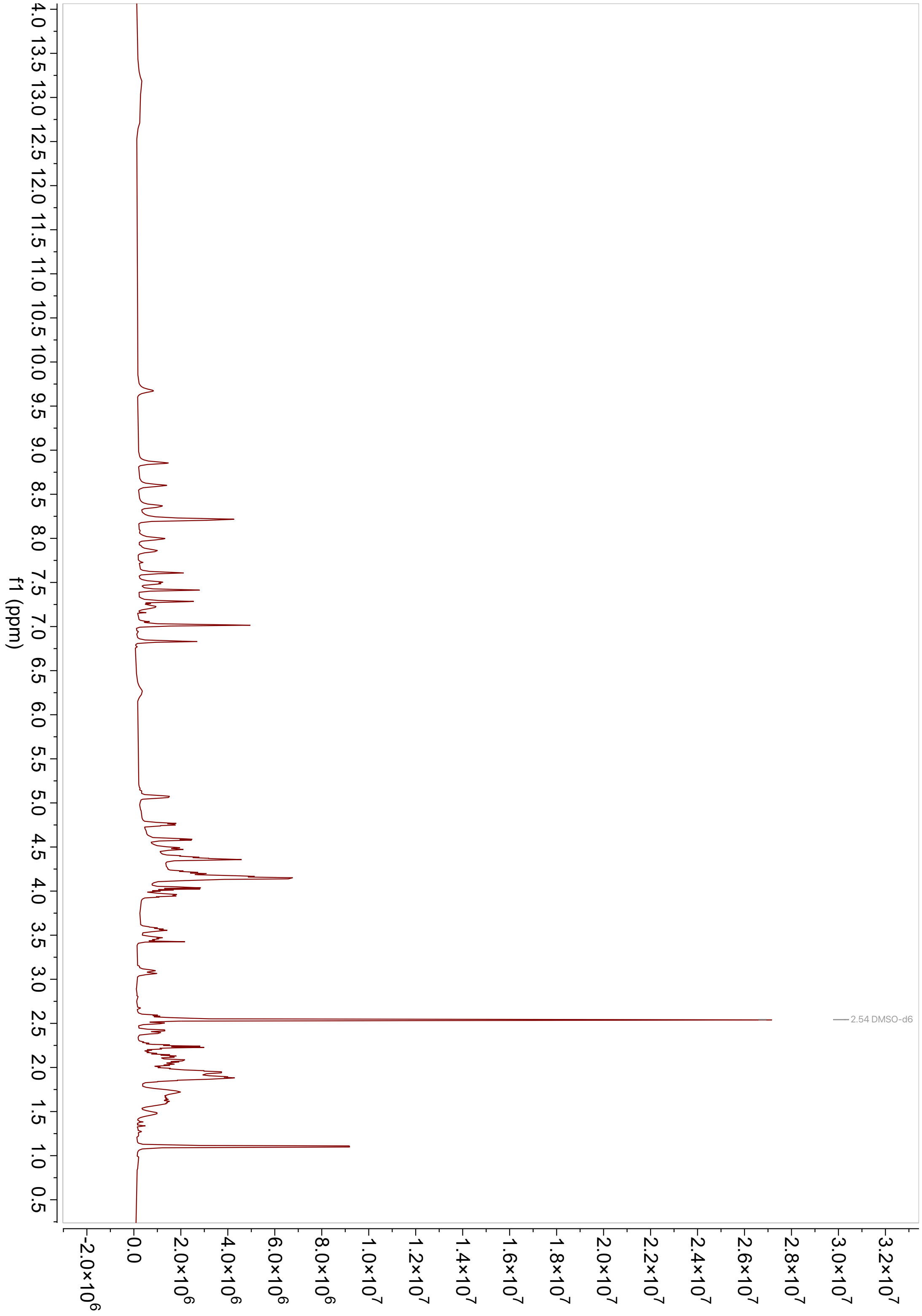

Figure S13. ^{1}H NMR spectrum of tistrellabactin B, in DMSO-d_6, collected on Bruker 500 MHz spectrometer.

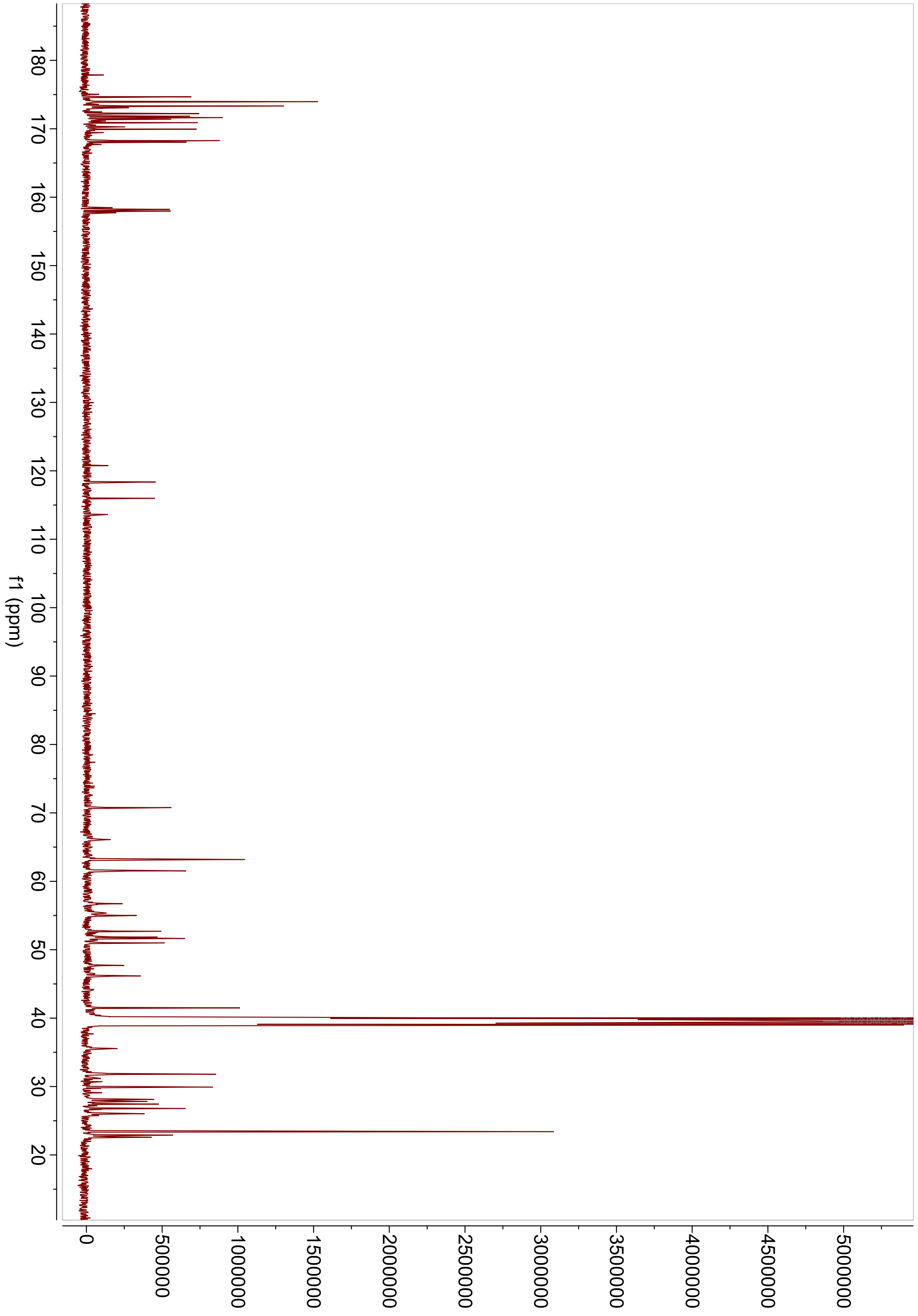

Figure S14. ^{13}C NMR spectrum of tistrellabactin B in DMSO-d_6, collected on Bruker 500 MHz spectrometer. Residual trifluoroacetic acid (used in HPLC purification) ^{13}C shifts are observed due to coordination to the positively charged siderophore.

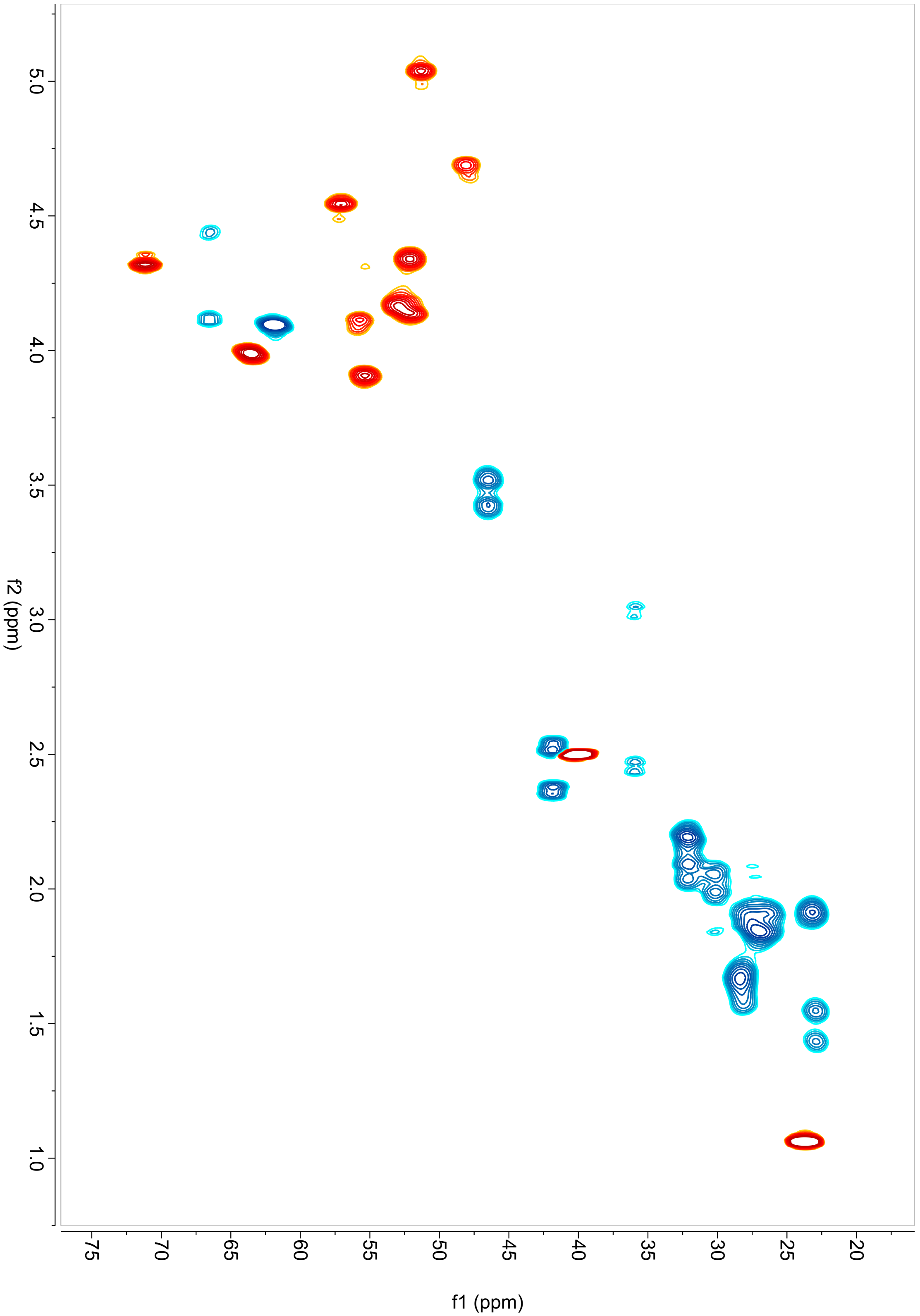

Figure S15. Multiplicity edited ^{1}H-^{13}C HSQC NMR spectrum of tistrellabactin B in DMSO-d_6, collected on Bruker 500 MHz spectrometer.

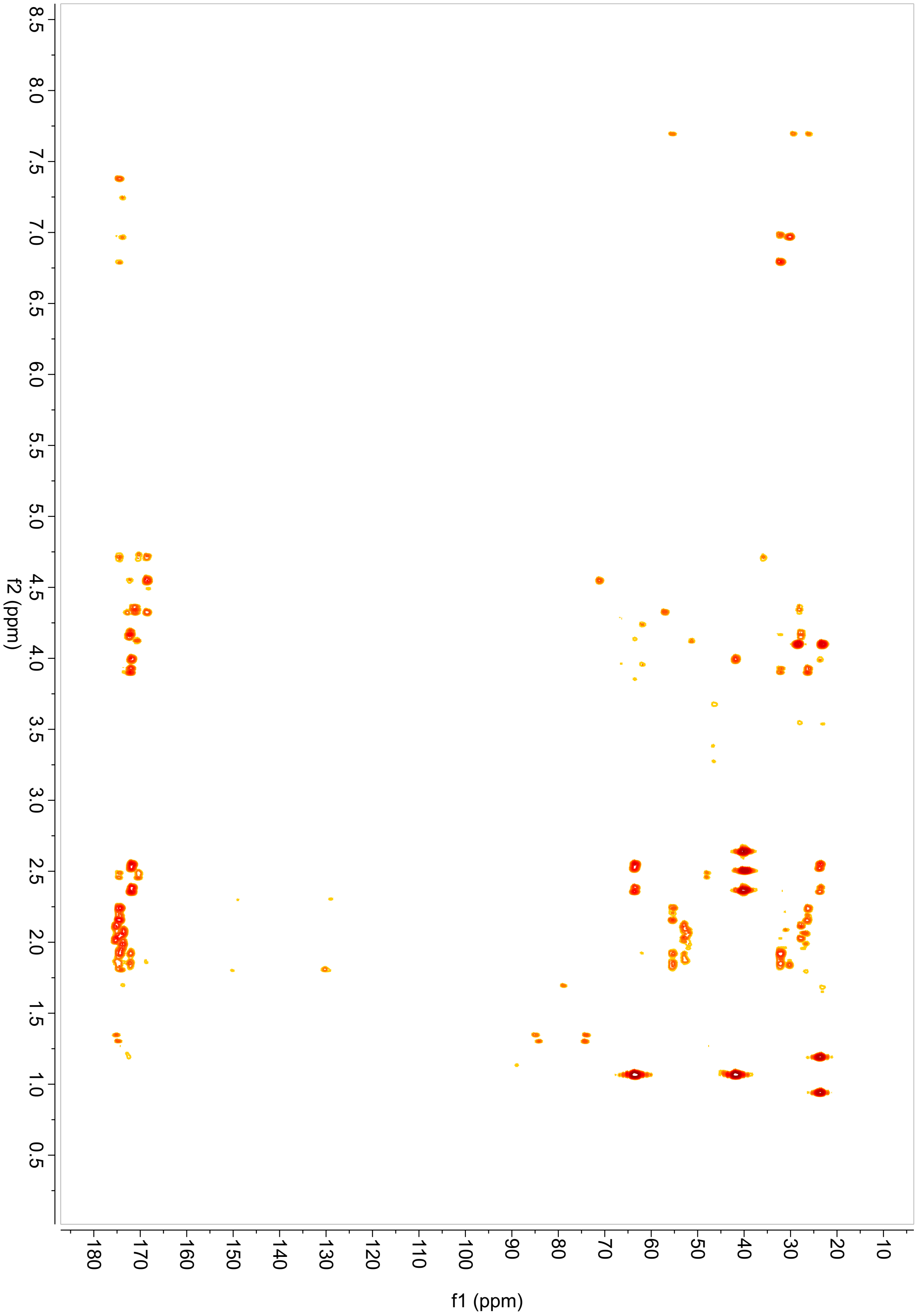

Figure S16. ^{1}H-^{13}C HMBC NMR spectrum of tistrellabactin B in DMSO-d_6, collected on Bruker 500 MHz spectrometer.

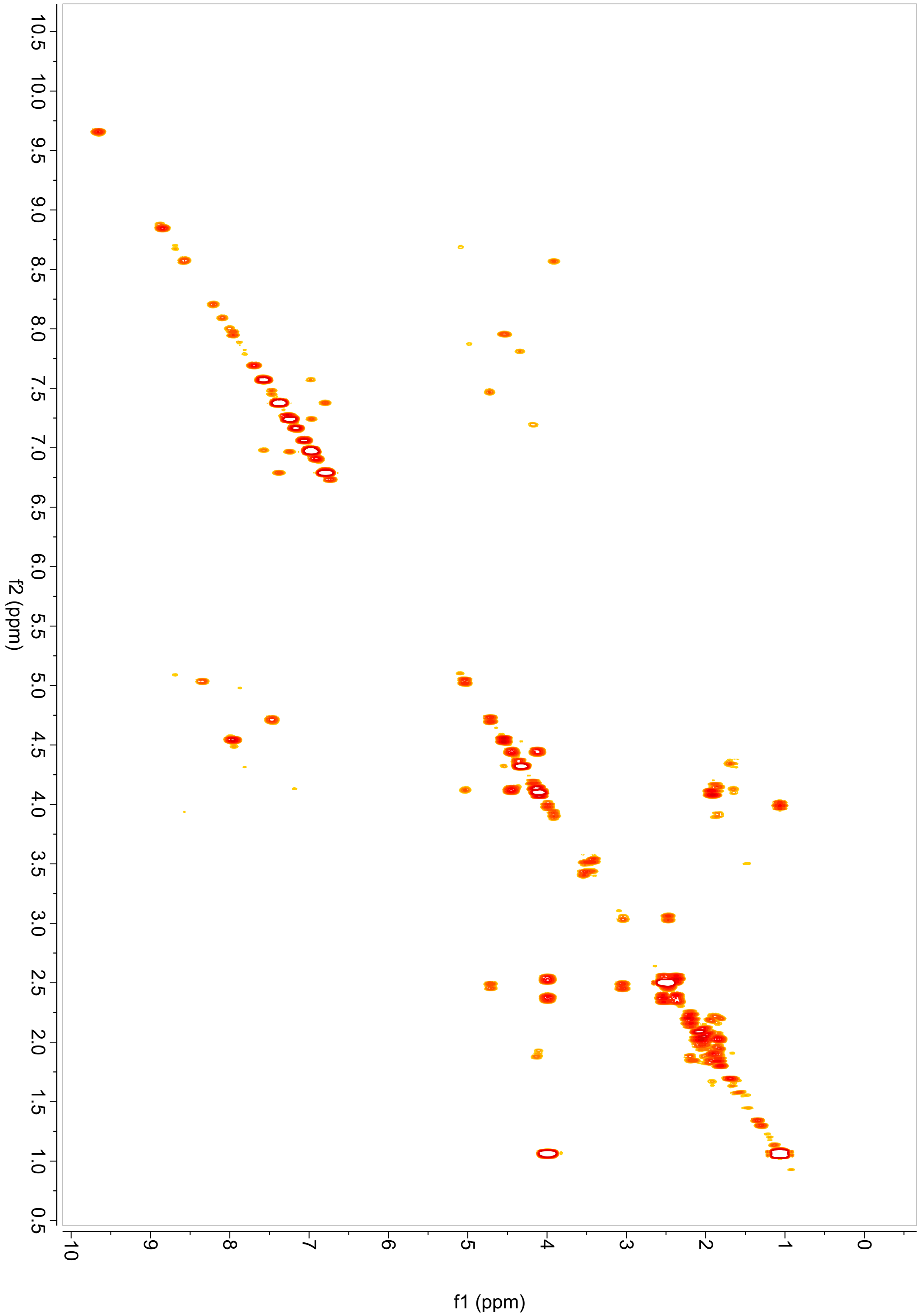

Figure S17. ^{1}H-^{1}H COSY NMR spectrum of tistrellabactin B in DMSO-d_6, collected on Bruker 500 MHz spectrometer.

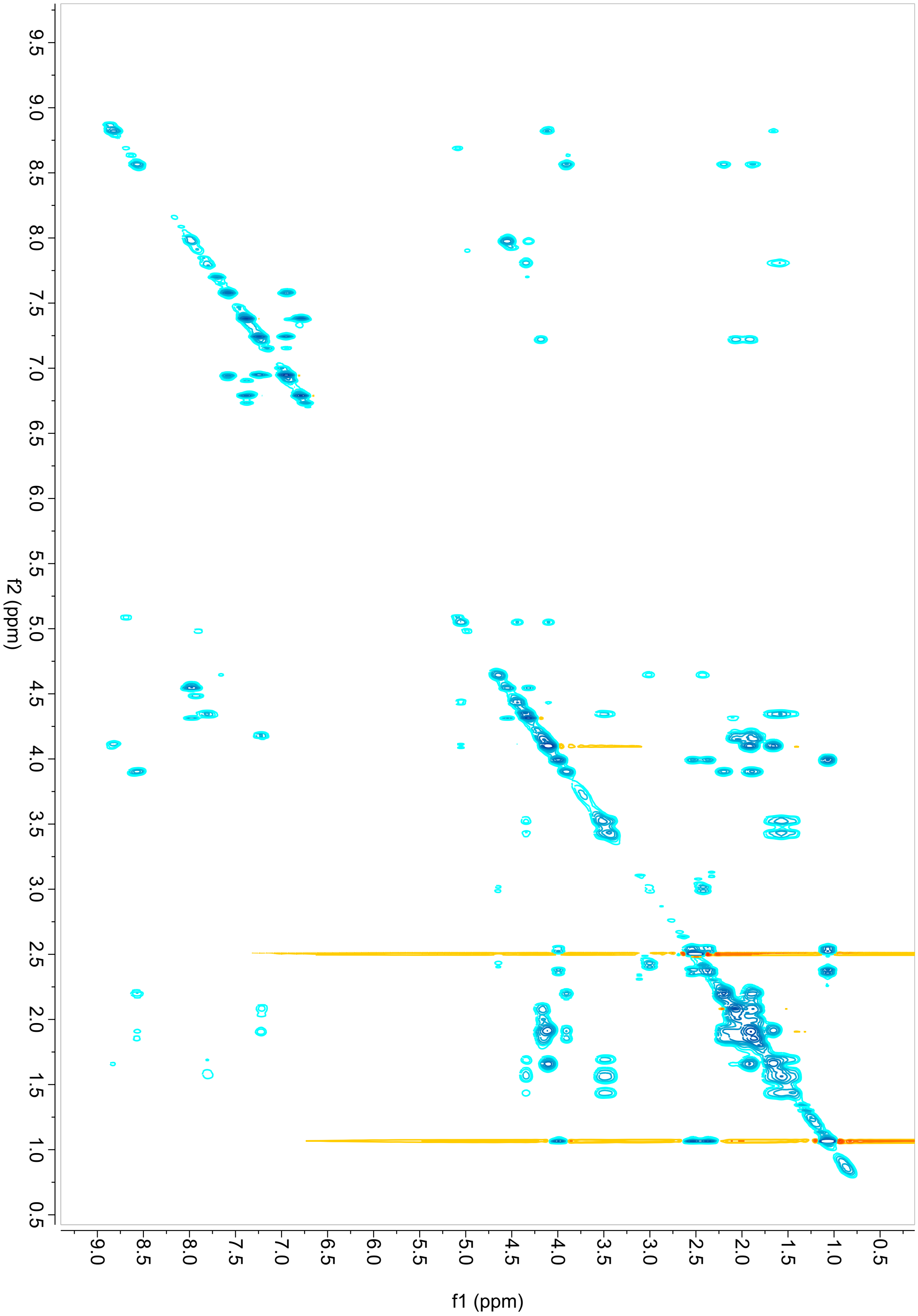

Figure S18. TOCSY NMR spectrum of tistrellabactin B in DMSO-d_6, collected on Bruker 500 MHz spectrometer.

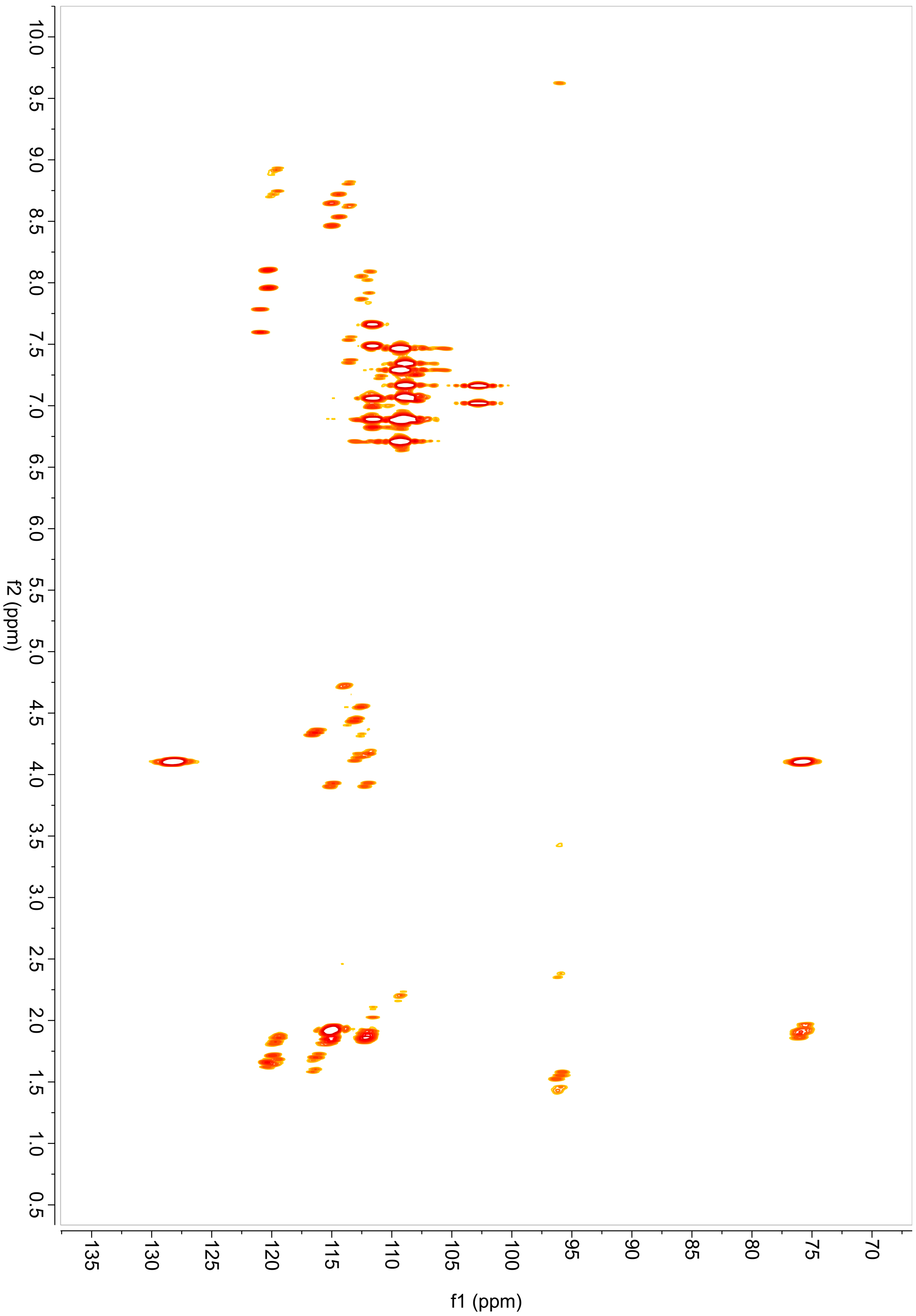

Figure S19. ^{1}H-^{15}N HMBC NMR spectrum of tistrellabactin B in DMSO-d_6, collected on Bruker 500 MHz spectrometer with ^{15}N spectral window set from 60-140 ppm.

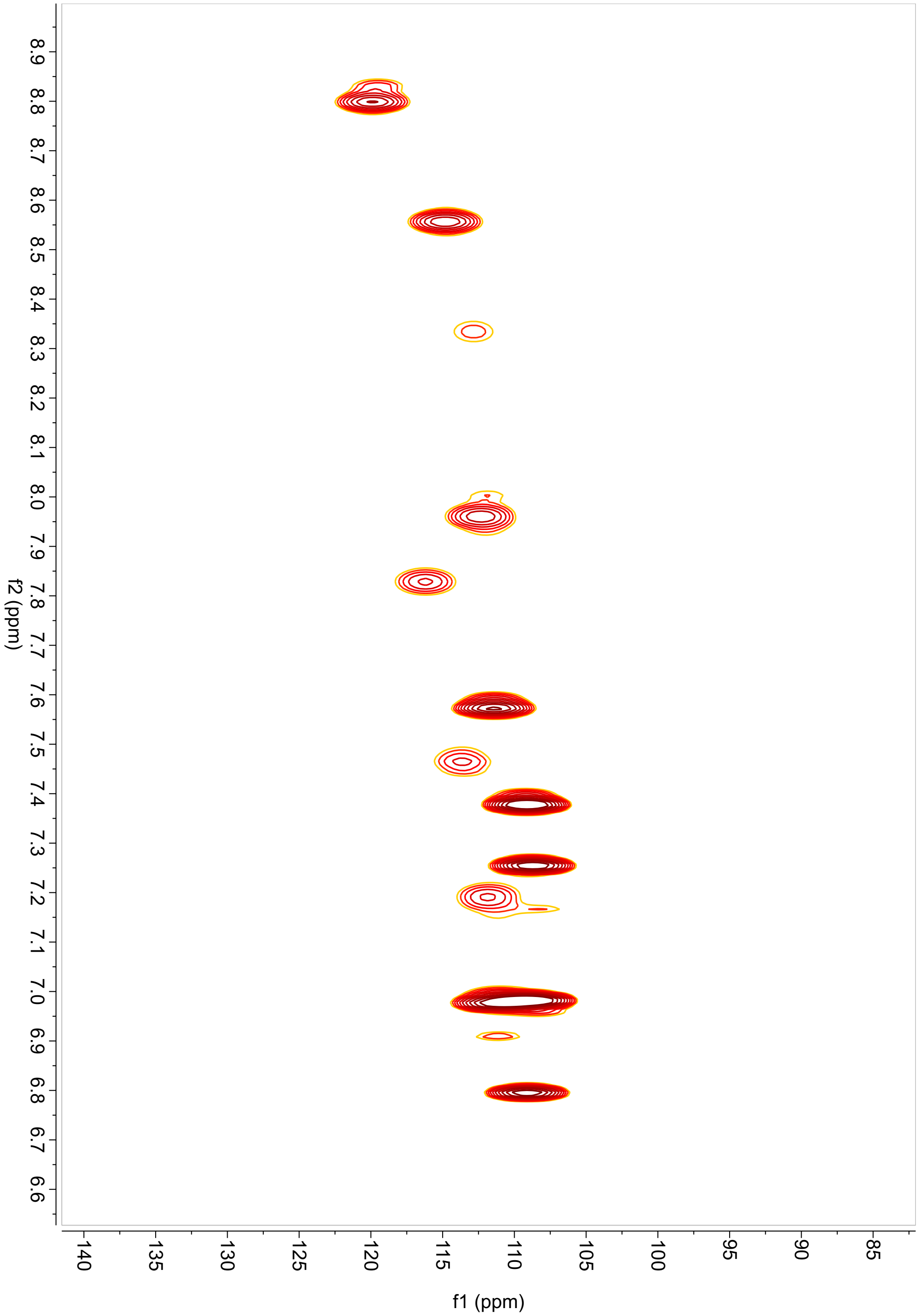

Figure S20. ^{1}H-^{15}N HSQC NMR spectrum of tistrellabactin B in DMSO-d_6, collected on Bruker 500 MHz spectrometer.

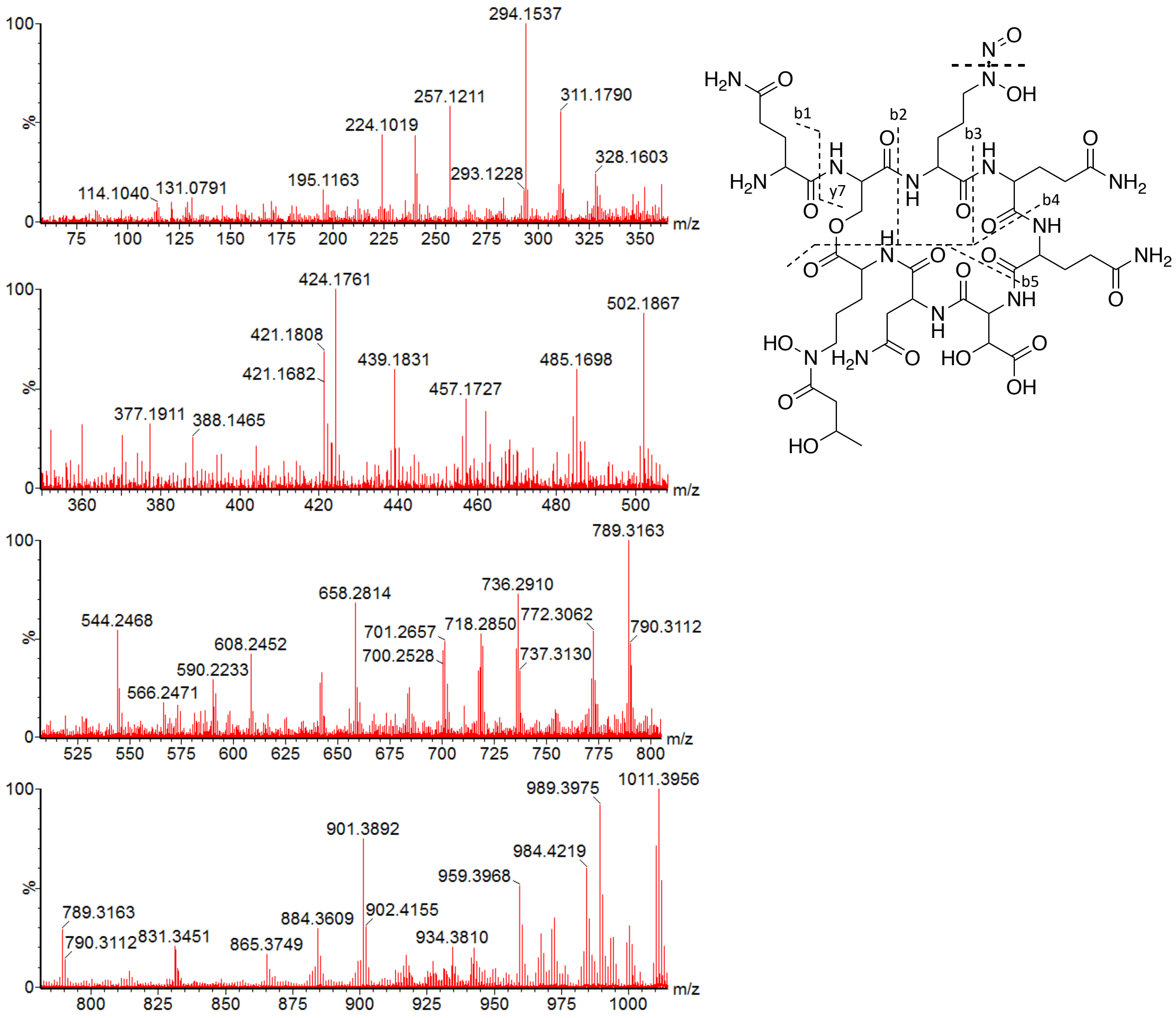

**b1**	*m/z* 129			**y7**	*m/z* 934	**y7 – NH_3**	*m/z* 917
**b2**	*m/z* 216			**y6**	*m/z* 848	**y6 – NH_3,**	*m/z* 831
						**y6 – NH_3 – NH_3**	*m/z* 814
**b3**	*m/z* 345	**b3 – NH_3,**	*m/z* 328	**y5**	*m/z* 718	**y5 – NH_3**	*m/z* 701
		**b3 – NH_3 – NH_3**	*m/z* 311				
**b4**	*m/z* 473	**b4 – NH_3 – NH_3**	*m/z* 439	**y4**	*m/z* 590	**y4 – NH_3**	*m/z* 573
**b5**	*m/z* 601	**b5 – NH_3 – H_2O**	*m/z* 566	**y3**	*m/z* 463		
**b6**	*m/z* 732			**y2**	*m/z* 332		
**b7**	*m/z* 846			**y1**	*m/z* 218		

Figure S21. MSMS fragmentation of parent ion *m/z* 1062 (tistrellabactin A – NO). Most fragments observed show minus NH_3 mass fragments from loss of side chain amines with higher ion counts than the accompanying b/y mass fragment. Ion masses listed in red were not observed.

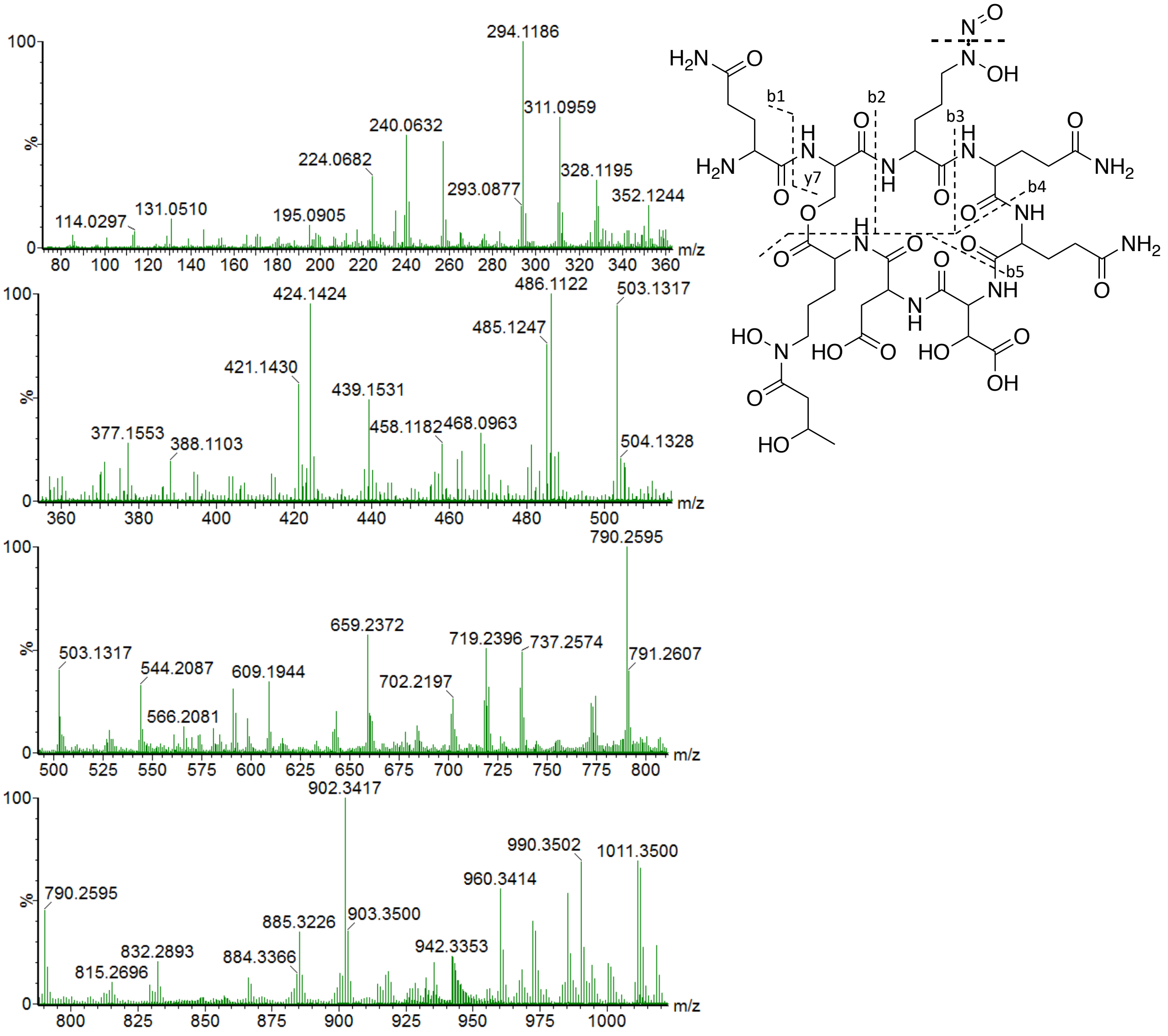

**b1**	*m/z* 129			**y7**	*m/z* 935	**y7 – NH_3**	*m/z* 918
**b2**	*m/z* 216			**y6**	*m/z* 849	**y6 – NH_3,**<br>**y6 – NH_3 – NH_3**	*m/z* 832<br>*m/z* 815
**b3**	*m/z* 345	**b3 – NH_3,**<br>**b3 – NH_3 – NH_3**	*m/z* 328<br>*m/z* 311	**y5**	*m/z* 719	**y5 – NH_3**	*m/z* 702
**b4**	*m/z* 473	**b4 – NH_3 – NH_3**	*m/z* 439	**y4**	*m/z* 591	**y4 – NH_3**	*m/z* 574
**b5**	*m/z* 601	**b5 – NH_3 – H_2O**	*m/z* 566	**y3**	*m/z* 464		
**b6**	*m/z* 732			**y2**	*m/z* 333		
**b7**	*m/z* 847			**y1**	*m/z* 218		

Figure S22. MSMS fragmentation of parent ion *m/z* 1063 (tistrellabactin B – NO). Most fragments observed show minus NH_3 mass fragments from loss of side chain amines with higher ion counts than the accompanying b/y mass fragment. Ion masses listed in red were not observed.

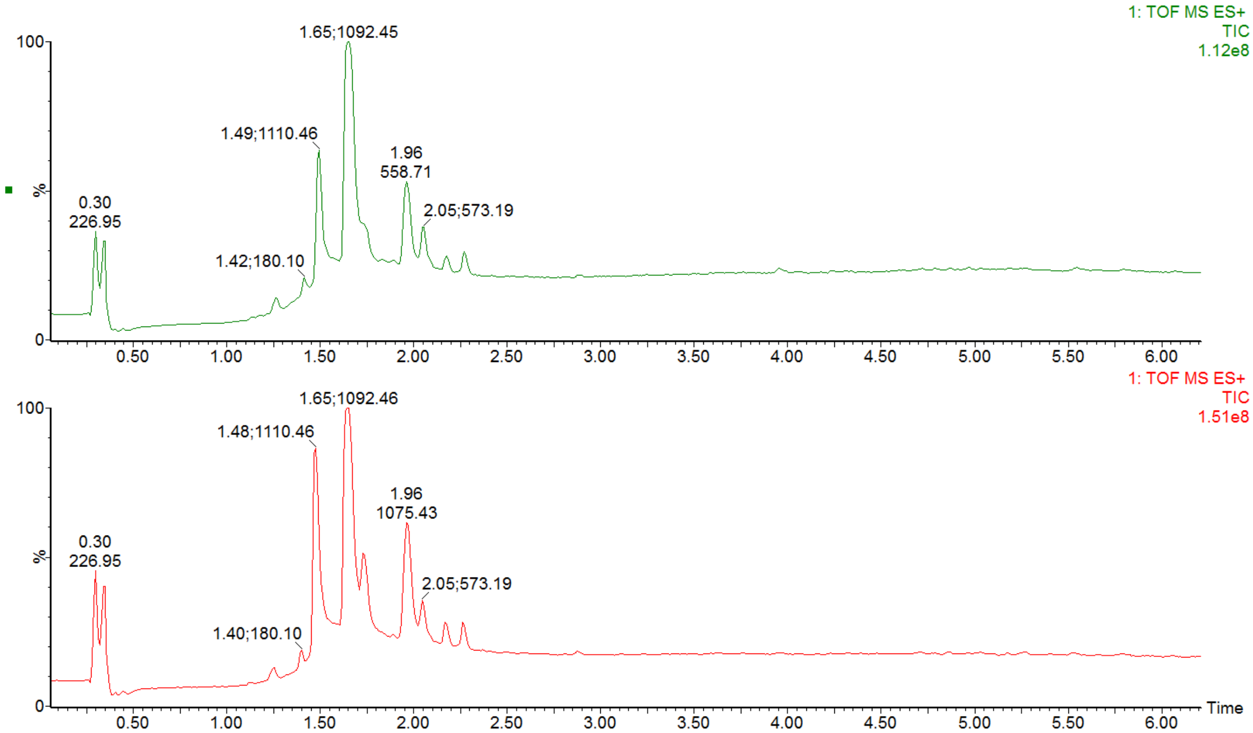

Figure S23. Tistrellabactin A incubated in Na_2HPO_4 buffer (pH 8) undergoes cleavage of the ester bond to yield the linear form (*m/z* 1110 $[M+H]^+$), with more forming with additional time as observed with the comparison of total ion chromatograms (TIC) between 28 hours (top) and 60 hours (bottom).

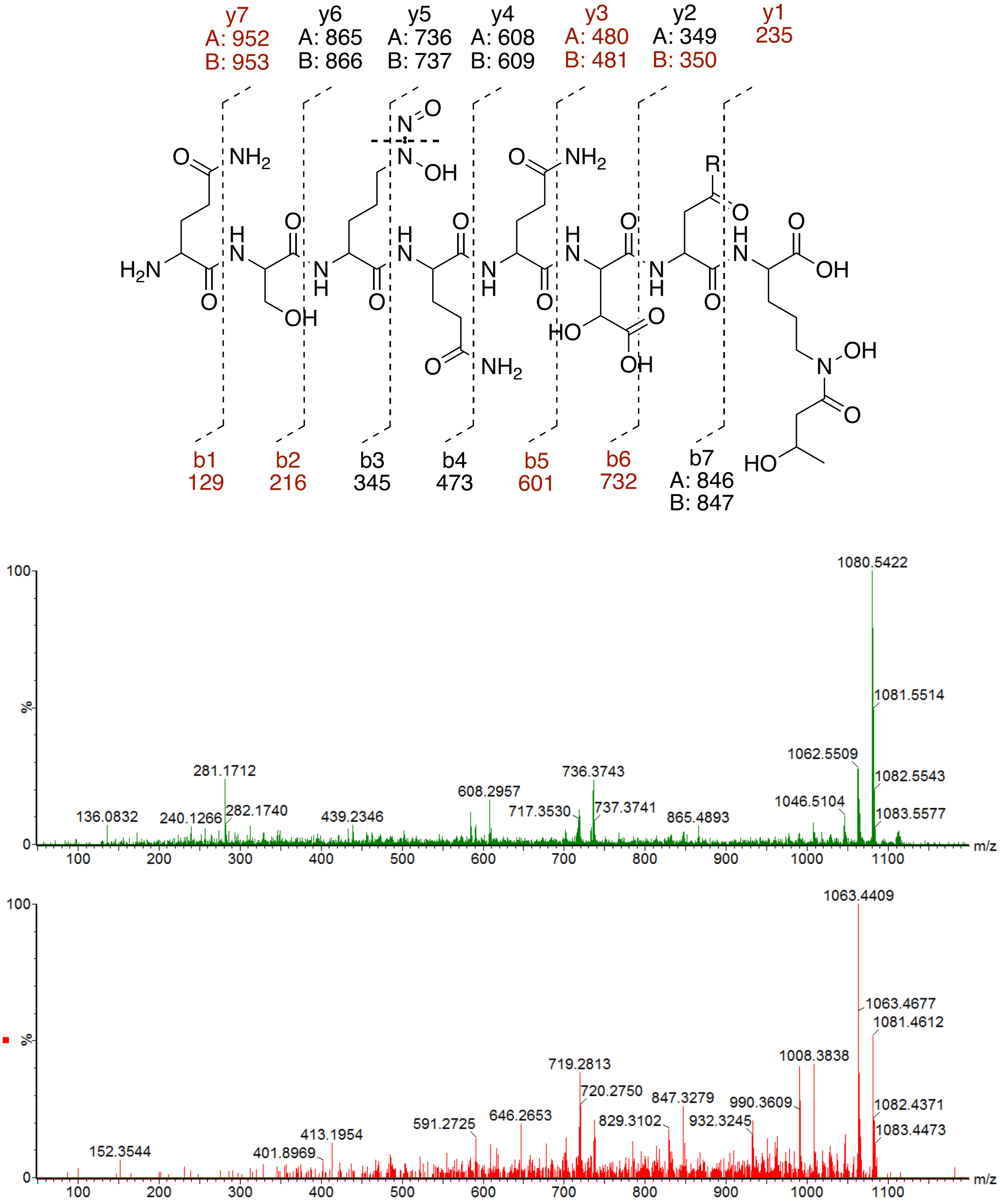

Figure S24. MSMS fragmentation of linear tistrellabactin A - NO (top, parent ion *m/z* 1080) and linear tistrellabactin B - NO (bottom, parent ion *m/z* 1081).

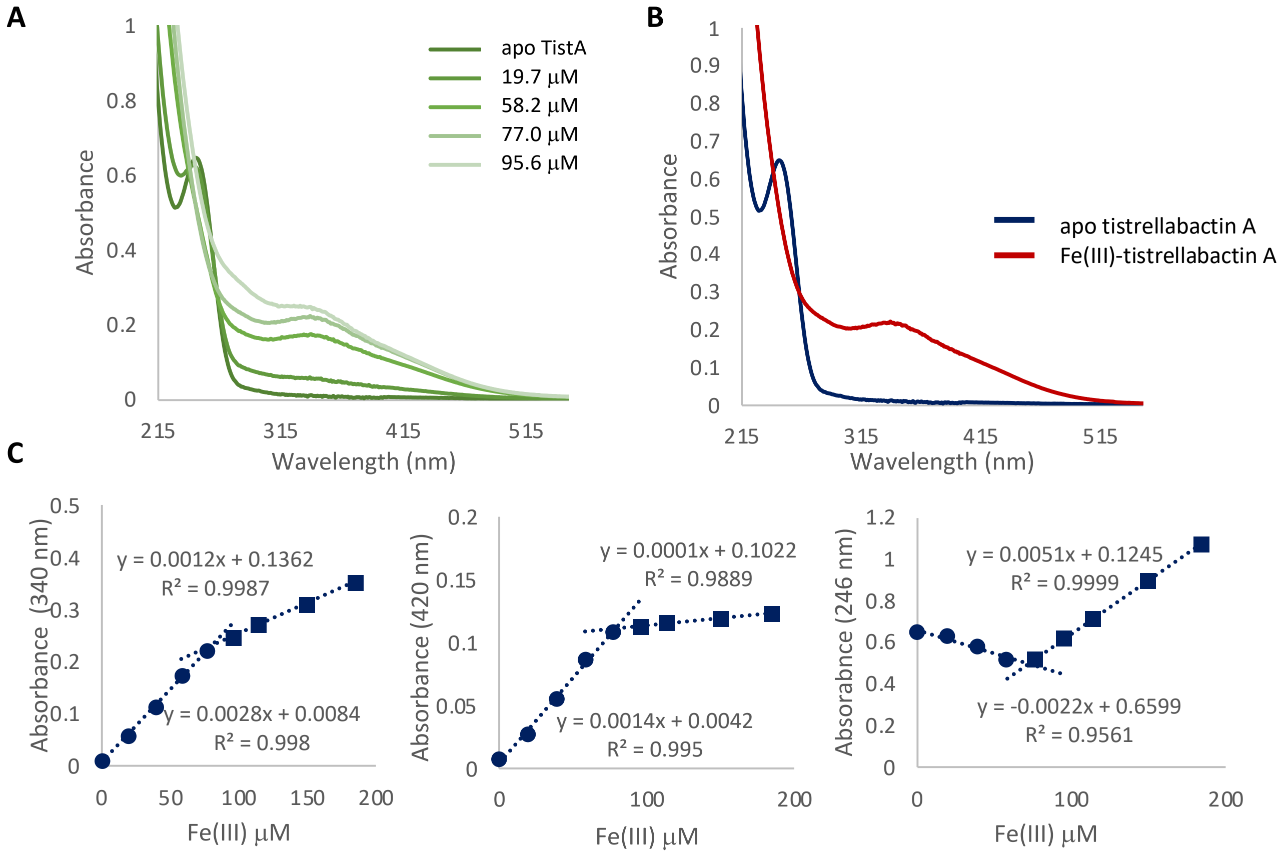

Figure S25. Fe(III) coordination to tistrellabactin A (TistA). (A) Titration of 85 μM apo-tistrellabactin A (citrate phosphate, pH 8) with standardized Fe(III) (2.58 mM ± 0.04). (B) UV-Vis spectra of apo-tistrellabactin A and Fe(III)-tistrellabactin A at the 1:1 Fe-siderophore breakpoint showing LMCT absorption bands at 420 nm (hydroxamate-Fe(III)) and 340 nm (α-hydroxycarboxylate-Fe(III)) and the disappearance of the C-diazeniumdiolate absorption band at 246 nm. (C) Titration of 85 μM apo-tistrellabactin A followed at 340 nm, 420 nm, and 246 nm as a function of standardized Fe(III) added.

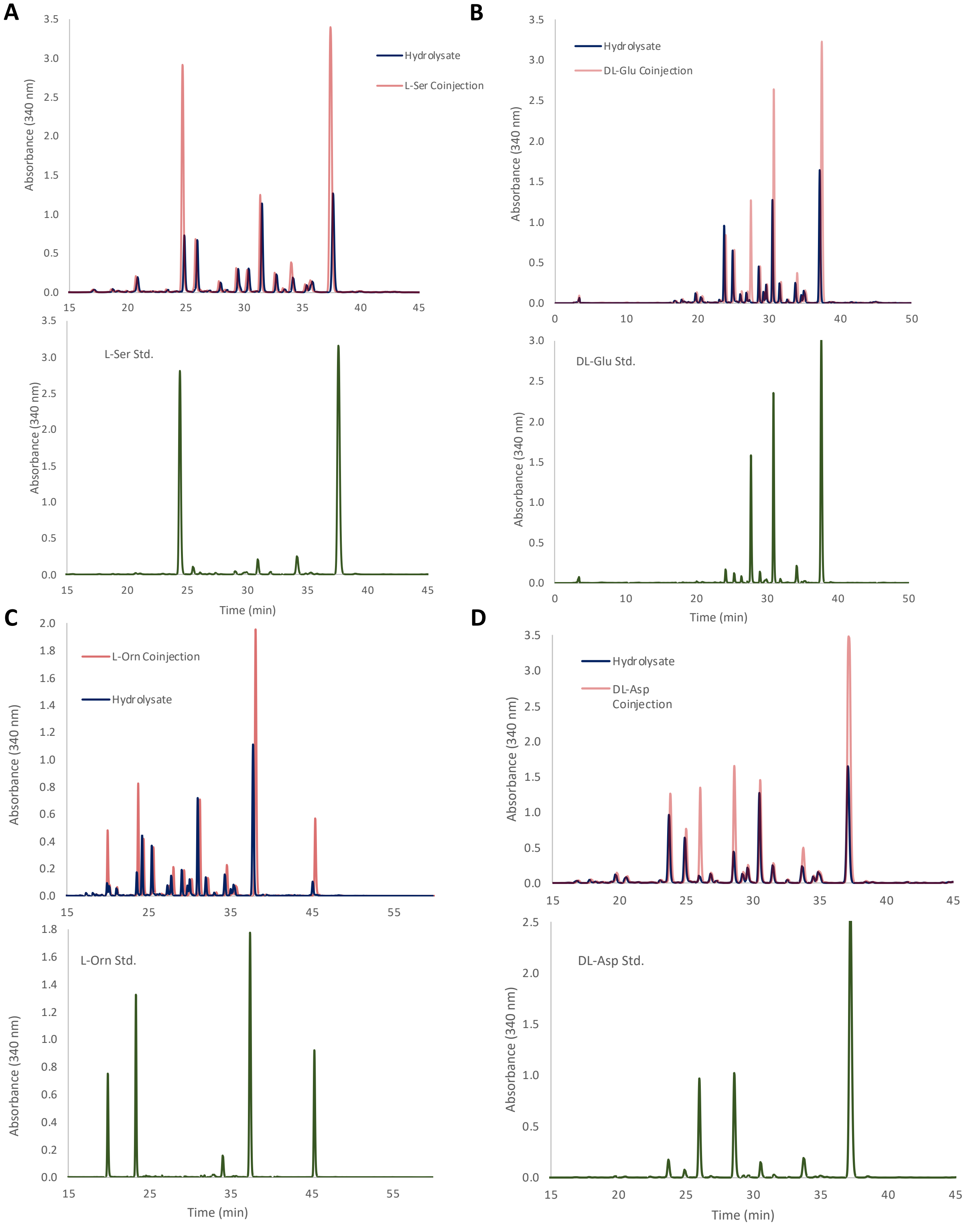

Figure S26. Marfey's amino acid analysis of tistrellabactin A by HPLC. (A) Tistrellabactin A FDAA-derivatized-hydrolysate coinjected with FDAA-L-Ser aligns with the FDAA-L-Ser standard, (B) FDAA-D-Glu standard (C) FDAA-L-Orn standard, (D) and FDAA-D-Asp standard. Peak at ~37 minutes present in all spectra is excess Marfey's chiral reagent (FDAA). FDAA-L-Glu and FDAA-L-Asp elute before the D configuration.[3]

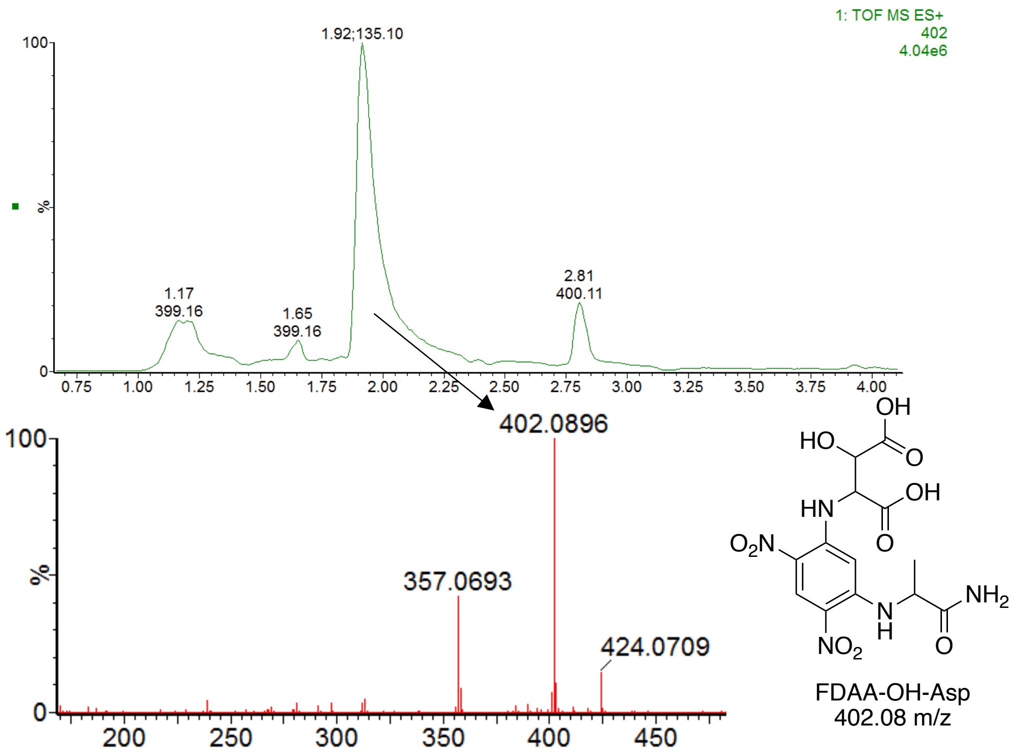

Figure S27. Marfey's amino acid analysis of tistrellabactin A FDAA-hydrolysate shows β-OH-Asp has L-*erythro* configuration. UPLC-MS extracted ion chromatogram (EIC, *m/z* 402) matches the retention time of FDAA-L-*erythro* β-OH-Asp standard as established in the literature under the same UPLC-MS conditions and same column (15-50% acetonitrile in 0.1% formic acid over 15 minutes).[1]

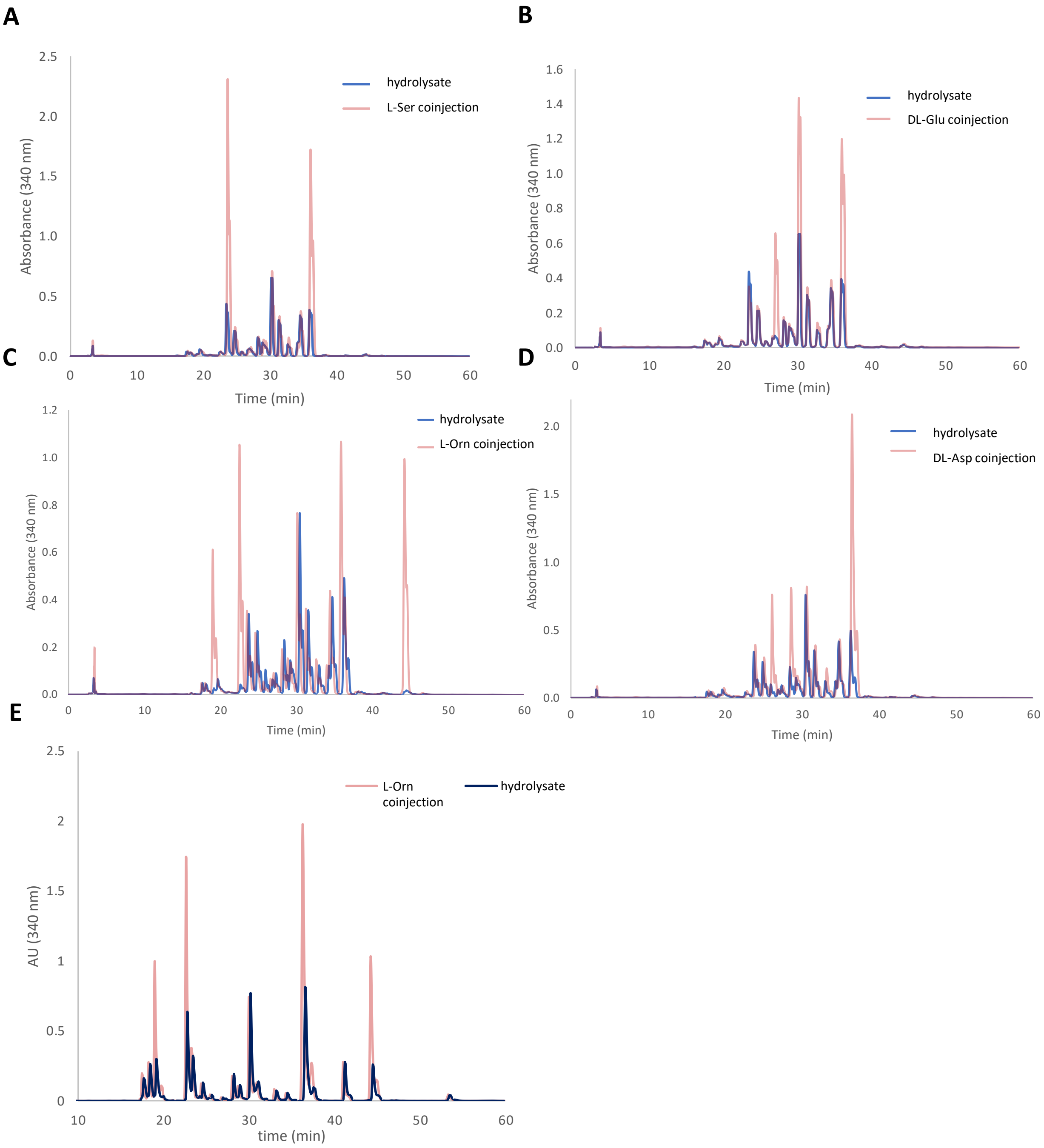

Figure S28. Marfey's amino acid analysis of tistrellabactin B by HPLC. (A) Tistrellabactin B HCl FDAA-derivatized-hydrolysate coinjected with FDAA-L-Ser aligns with the FDAA-L-Ser standard, (B) FDAA-D-Glu standard (C) FDAA-L-Orn standard, (D) and FDAA-D-Asp standard. Peak at ~37 minutes present in all spectra is excess Marfey's chiral reagent (FDAA). FDAA-L-Glu and FDAA-L-Asp elute before the D configuration.[3] (E) FDAA-tistrellabactin B hydrolyzed in 45% HI coinjected with FDAA-L-Orn.

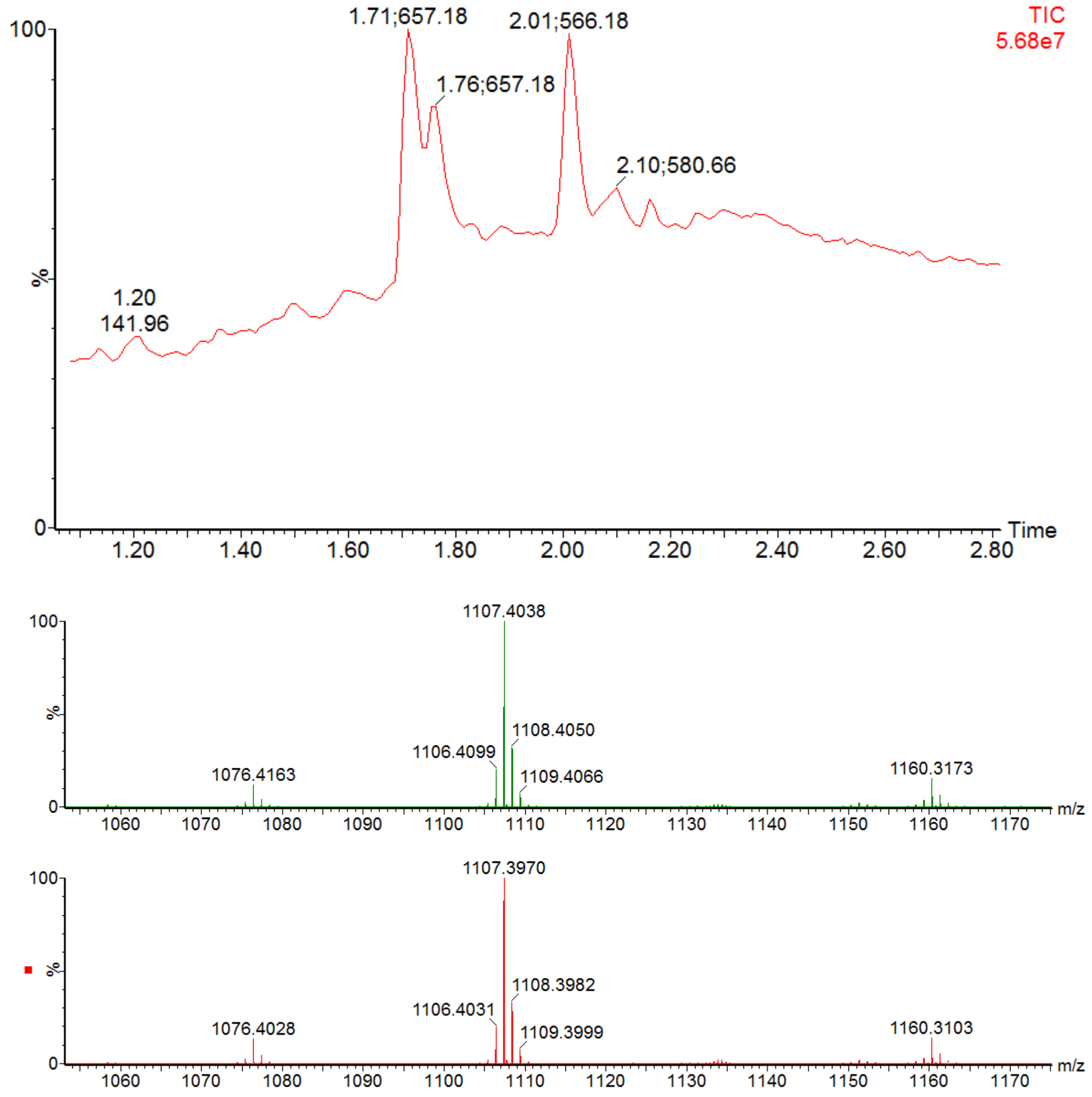

Figure S29. UPLC-MS analysis of *Tistrella mobilis* KA081020-065 grown with $^{15}NH_4Cl$ as the sole nitrogen source. (A) The total ion chromatogram of the extracted ^{15}N enriched *T. mobilis* supernatant shows two peaks eluting at 1.71 and 1.76 min both with the protonated molecule *m/z* 1107 $[M+H]^+$. (B) ^{15}N-tistrellabactin A (top, Rt 1.71 min) and ^{15}N-Tistrellabactin B (bottom, Rt 1.76 min) with corresponding ionization-induced mass losses of 31 (^{15}NO, *m/z* 1076.4). Isotopic labeling shows tistrellabactin A contains 15 nitrogens while tistrellabactin B contains 14 nitrogens.

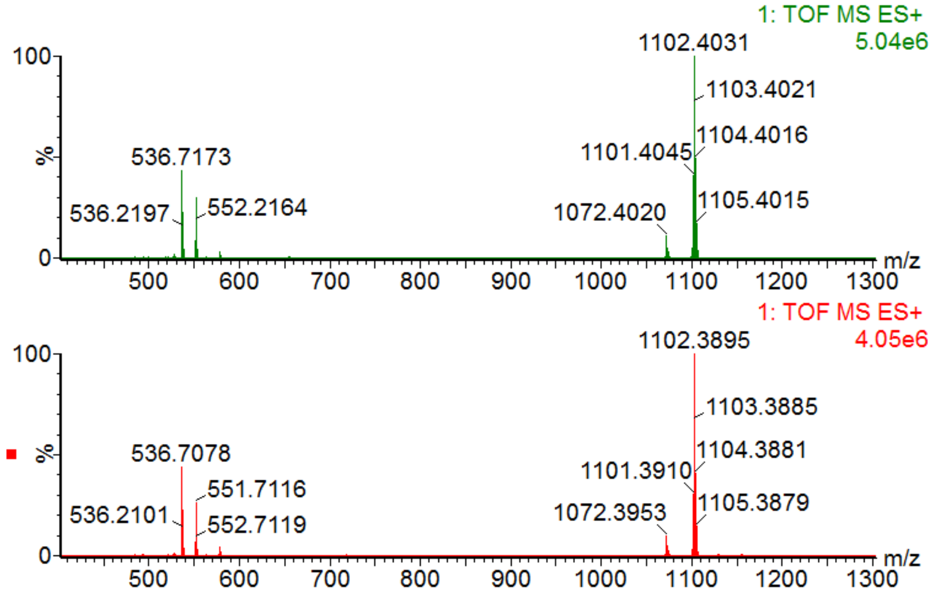

Figure S30. UPLC-MS analysis of *Tistrella mobilis* KA081020-065 grown with $^{15}NH_4Cl$ and ^{14}N-L-Arg as the nitrogen sources. The resulting purified ^{15}N-enriched tistrellabactin A (top, green) and ^{15}N-enriched tistrellabactin B (bottom, red) both have an isotopic protonated molecule of *m/z* 1102 $[M+H]^+$ with a mass loss of 30 indicating the distal nitrogen on the Gra is unlabeled and originating from L-Arg.

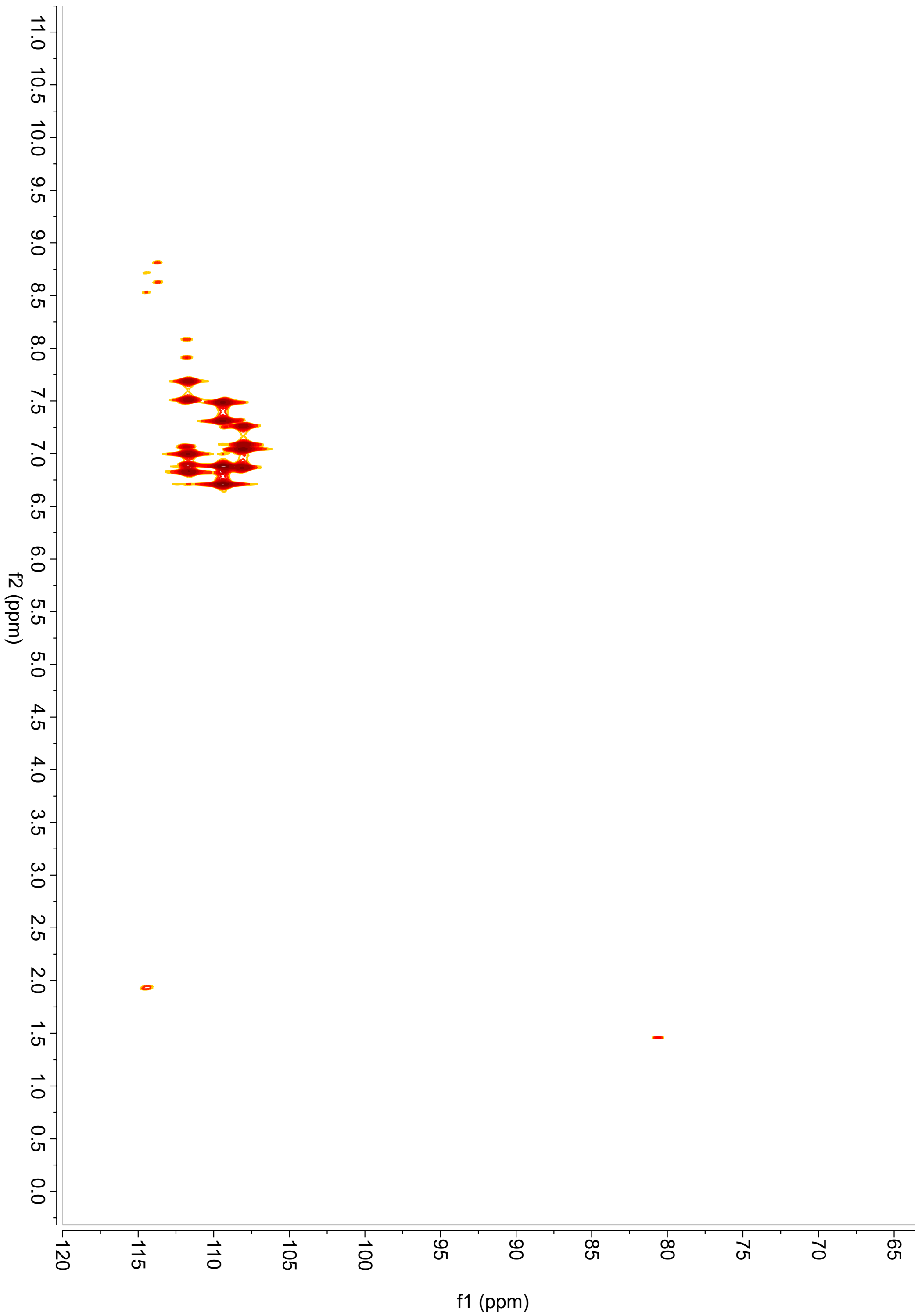

Figure S31. ^{1}H-^{15}N HMBC NMR spectrum of ^{15}N-enriched tistrellabactin A isolated from *T. mobilis* grown with $^{15}NHCl_4$ and ^{14}N-L-Arg as the only two nitrogen sources. In comparison to the fully ^{15}N-enriched-tistrellabactin A ^{1}H-^{15}N HMBC spectrum, signals are missing for N4, N5, N6, N14, and N15 (Table S4), showing the Gra and Orn residues are unenriched. Spectrum collected in DMSO-d_6 on Bruker 500 MHz spectrometer.

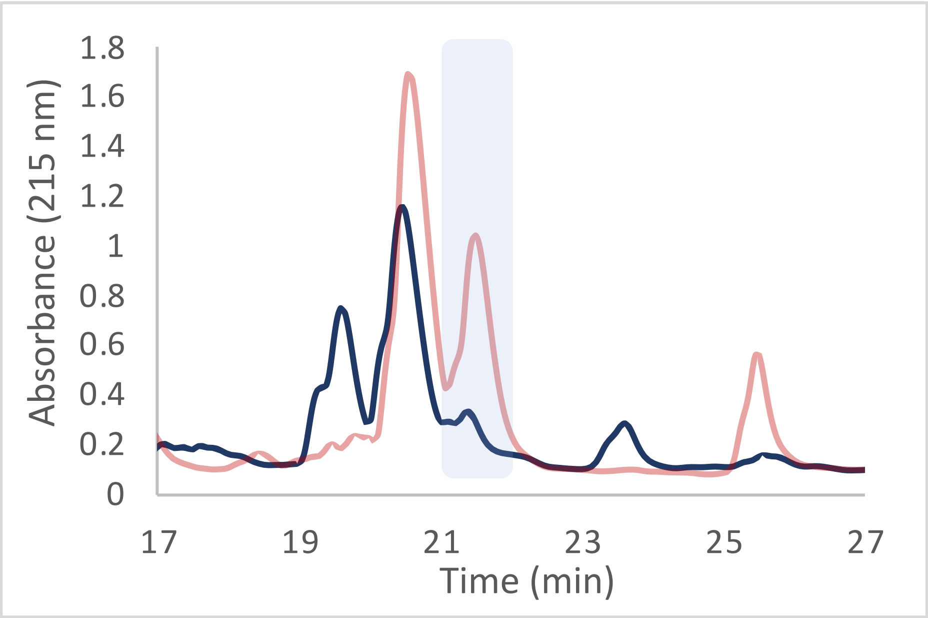

Figure S32. *T. mobilis* supplemented with 20 mM L-Asn (dark blue trace) shows increased favor for tistrellabactin A over tistrellabactin B, with the difference between normal growth conditions (red trace) and Asn supplemented growth highlighted in blue.

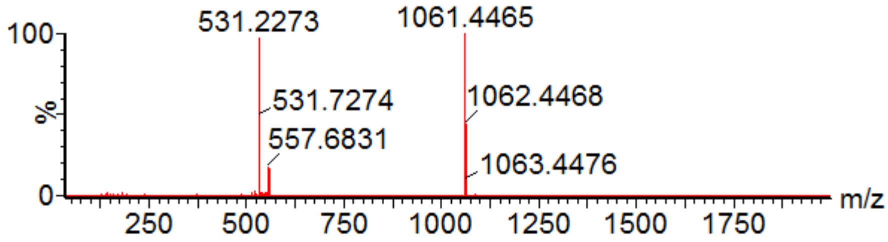

Figure S33. Mass spectrum of tistrellabactin A photoproduct (*m/z* 1061.4). Doubly charged protonated molecule of Fe(III) coordinated to the tistrellabactin A photoproduct is also present (*m/z* 557) demonstrating the retention of Fe(III) coordination upon photolysis.

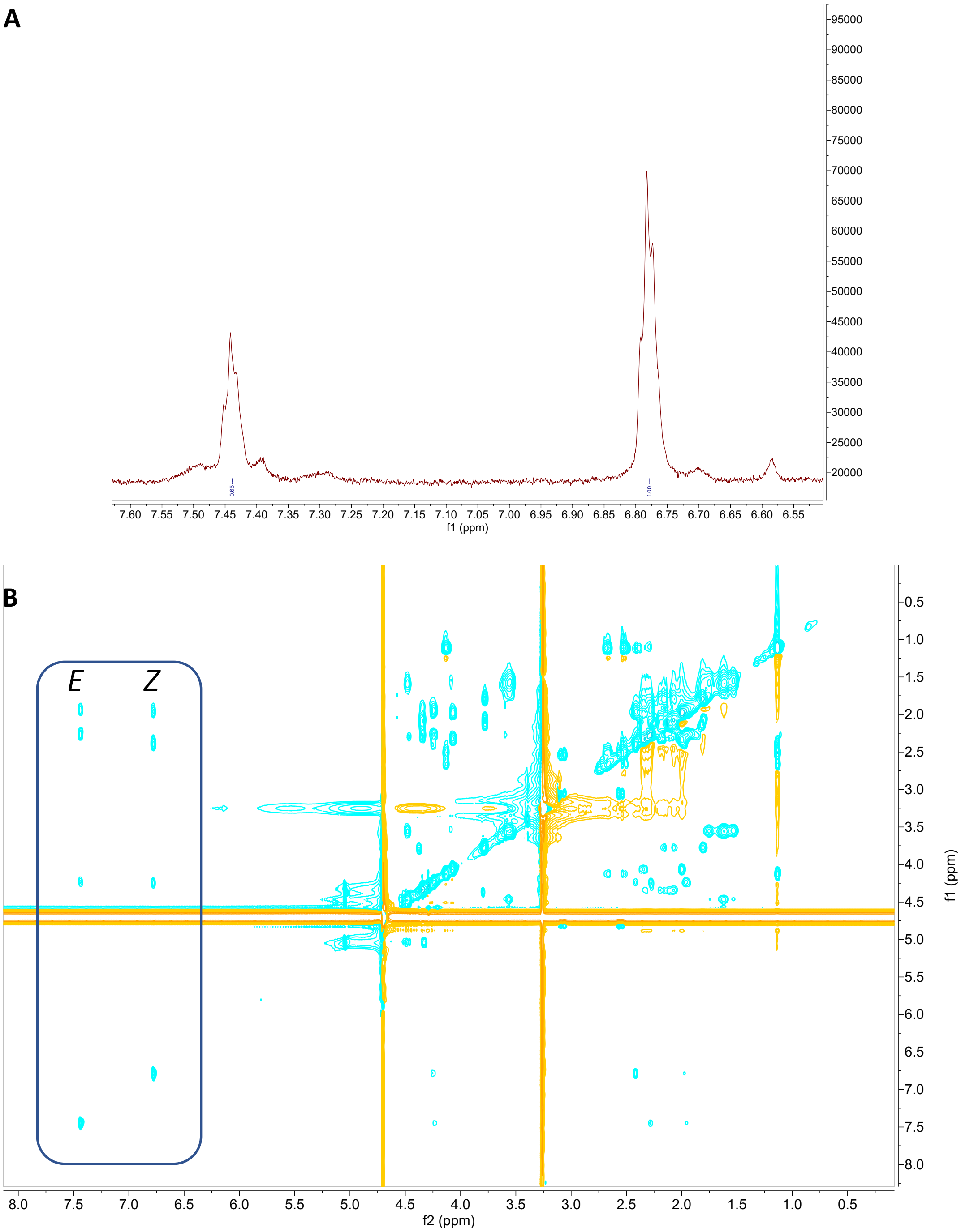

Figure S34. NMR analysis of apo-tistrellabactin A photoproduct. Spectra recorded in D_2O (99.9%) on Bruker 500 MHz spectrometer. (A) Upon irradiation of apo-tistrellabactin A with UV light (254 nm) two new ^{1}H resonances appear at 6.8 ppm (*Z*) and 7.5 ppm (*E*), consistent with the formation of *E*/*Z* oxime isomers, matching previously reported results for irradiated *C*-diazeniumdiolates.[2] (B) TOCSY spectrum of photolyzed apo-tistrellabactin A shows the Gra residue exists as a mixture of *E* and *Z* isomers as highlighted in the dark blue box.

A

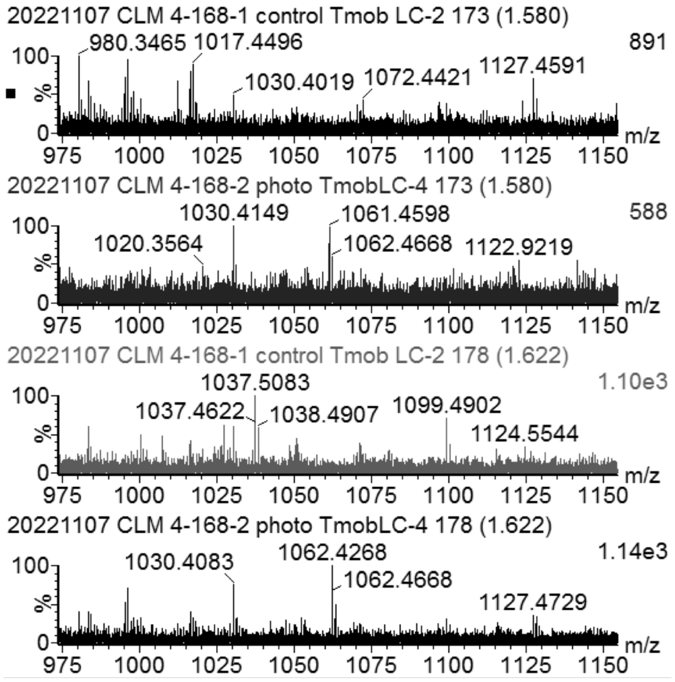

B

T.mobilis control (dark): 1.580 min.

T. mobilis SN photolyzed: 1.580 min.
tistrellabactin A photoproduct *m/z* 1061

T.mobilis control (dark): 1.622 min.

T. mobilis SN photolyzed: 1.622 min.
tistrellabactin B photoproduct *m/z* 1062

C

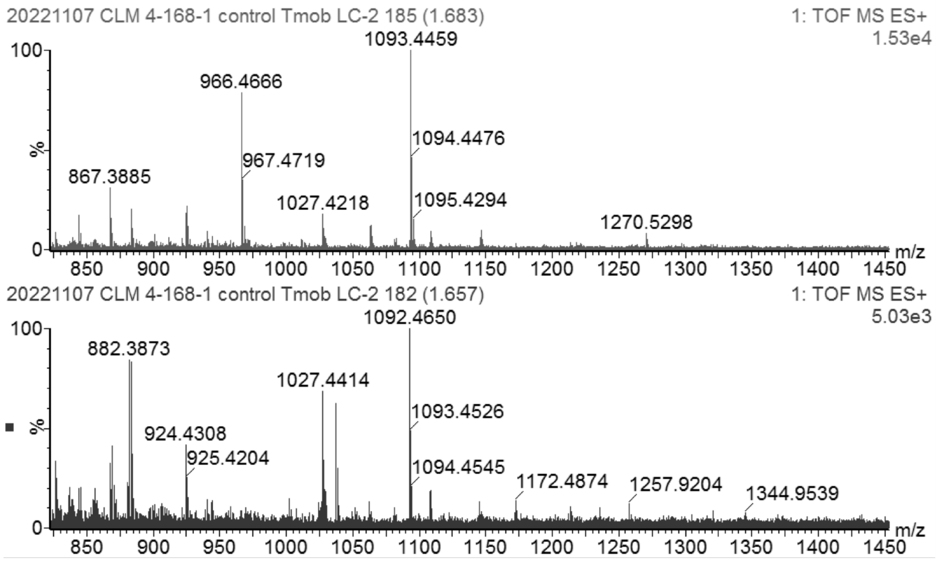

Figure S35. Mass spectra of aliquots removed from an actively growing culture of *T. mobilis*; one photolyzed with UV light and the other not irradiated. (A) Mass spectrum (retention time 1.58 min) of non-irradiated *T. mobilis* aliquot (top) does not have mass of apo-tistrellabactin A photoproduct, while the irradiated aliquot (bottom) does (*m/z* 1061.4). (B) Mass spectrum (retention time 1.62 min) of non-irradiated *T. mobilis* aliquot (top) does not have mass of apo-tistrellabactin B photoproduct, while the irradiated aliquot (bottom) does (*m/z* 1062.4). (C) Mass spectra of non-irradiated control aliquot of actively growing *T. mobilis* contains tistrellabactin A (*m/z* 1092.4) and tistrellabactin B (*m/z* 1093.4).

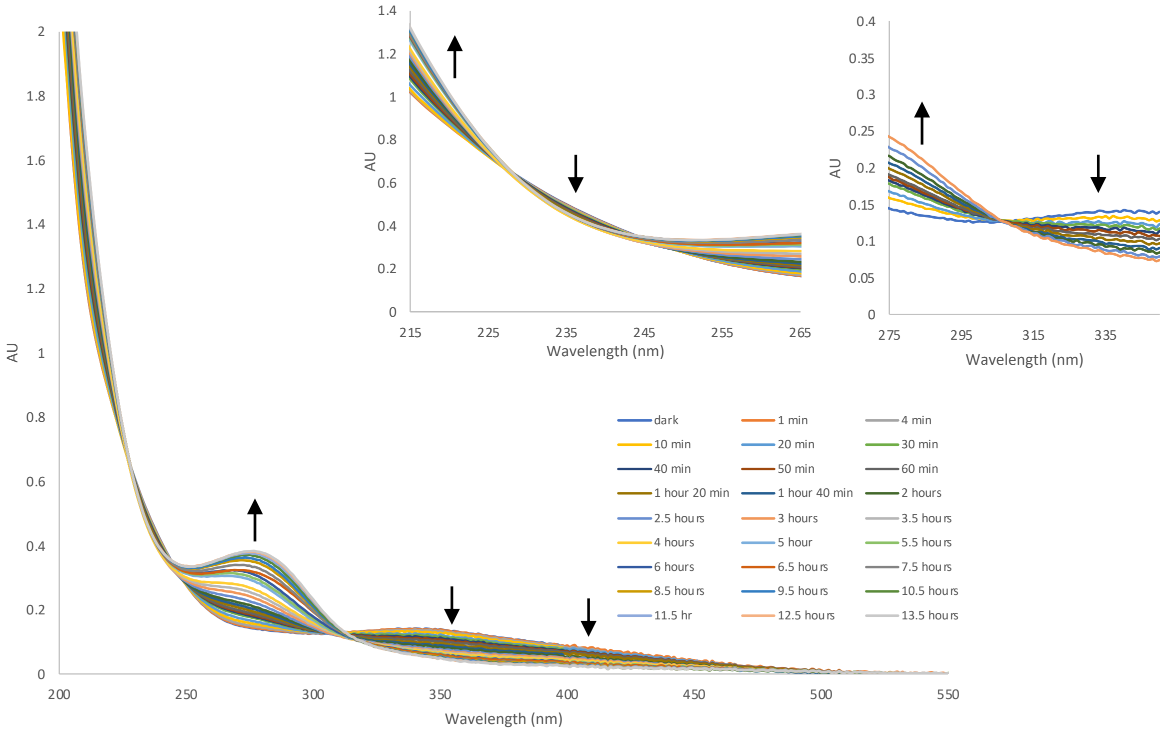

Figure S36. Photolysis of Fe(III)-tistrellabactin A (25 mM Na_2HPO_4, pH 8, Oriel Hg(Ar) pen lamp No. 6035).

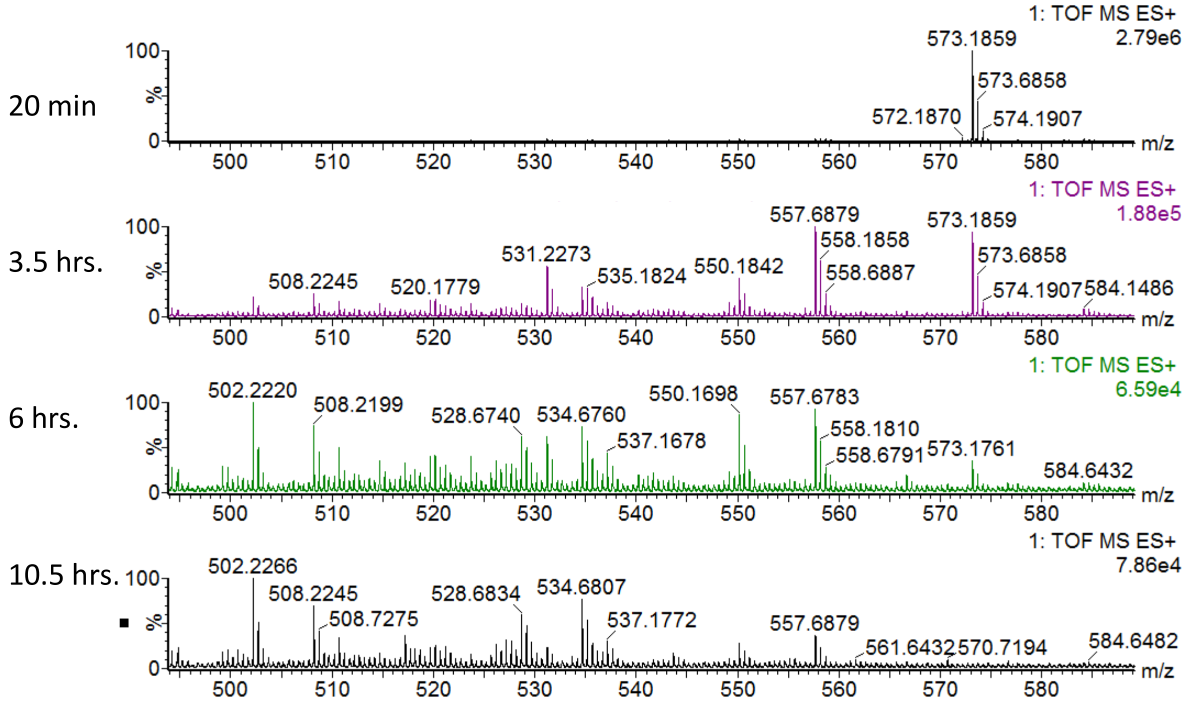

m/z 573 [(Fe(III) – TistA) + 2H]$^{2+}$
m/z 557 [(Fe-TistA$_{h\nu}$ – NO – H) + 2H]$^{2+}$
m/z 550 [(Fe-TistA$_{h\nu}$ – CO_2 – 2H) + 2H]$^{2+}$
m/z 531 [(TistA$_{h\nu}$ – NO – H) + 2H]$^{2+}$
m/z 508 [(TistA$_{h\nu}$ – NO – CO_2 – 3H) + 2H]$^{2+}$

Figure S37. Mass spectra of aliquots from Fe(III)-tistrellabactin A (TistA), photolyzed selectively at 254 nm with Oriel Hg(Ar) pen lamp No. 6035 as the UV light source. Aliquots were taken at 20 minutes, 3.5 hours, 6.5 hours, and 10.5 hours.

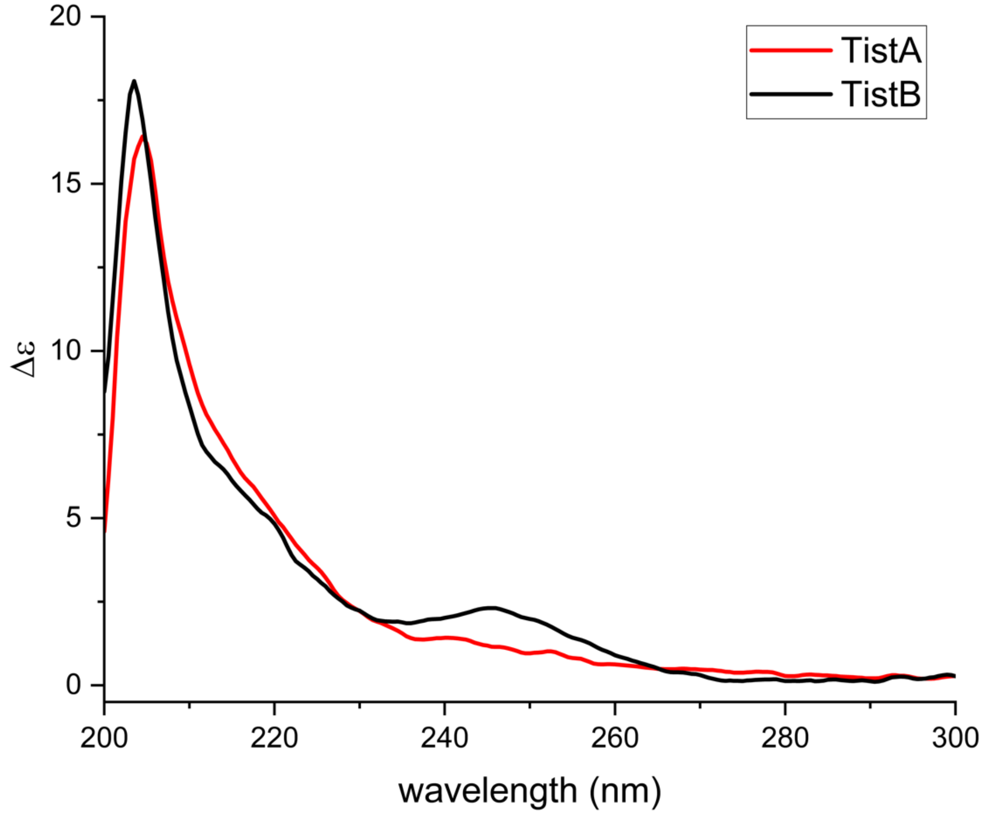

Figure S38. Circular dichroism spectrum of tistrellabactins A (TistA) and tistrellabactin B (TistB).

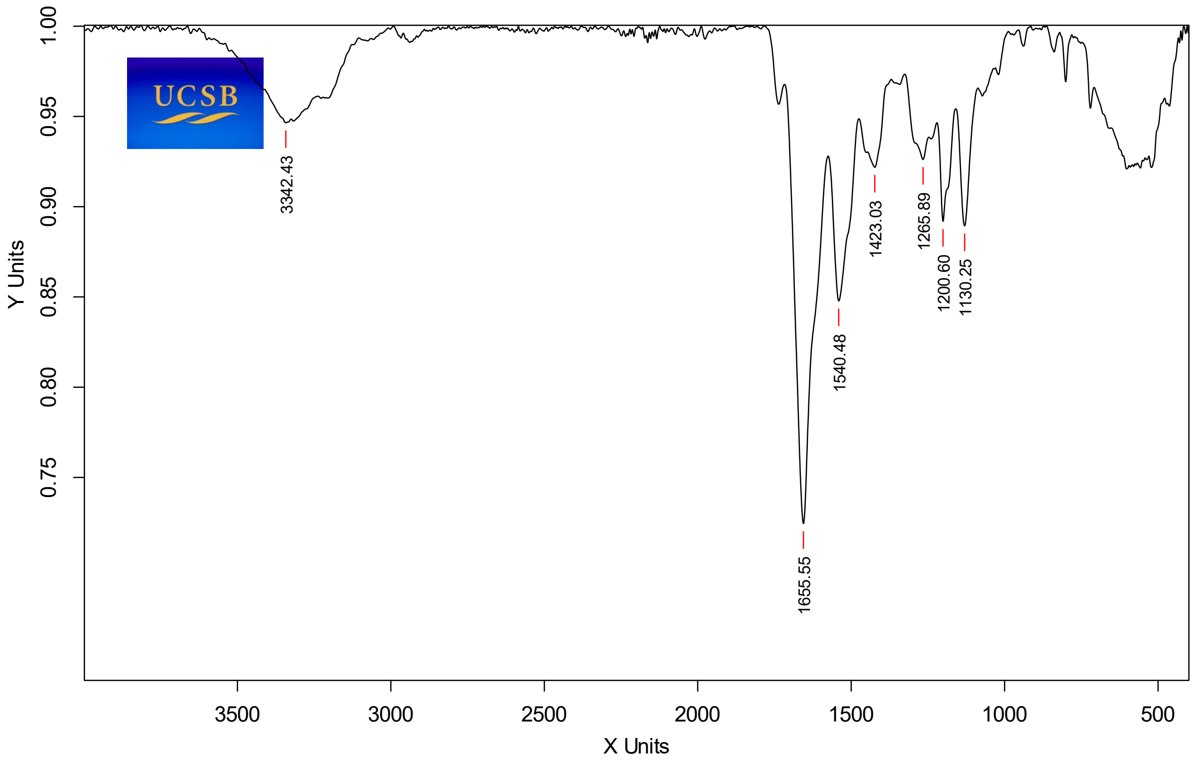

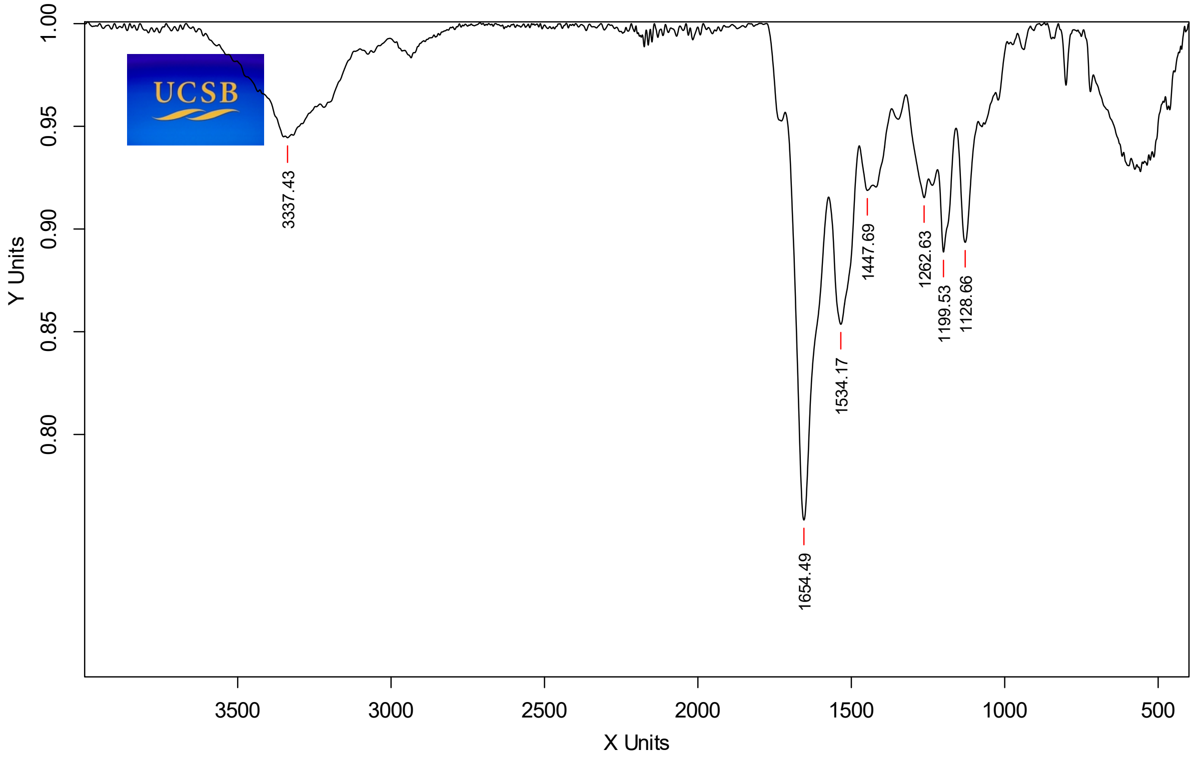

Figure S39. IR spectra of tistrellabactin A (top) and tistrellabactin B (bottom).

Table S1. Gene cluster annotation for the tistrellabactins isolated from *T. mobilis*[4]

	Protein ID	Size(AA)	Annotated product (antiSMASH)
MobA	WP_014753078.1	237	3-oxoacid CoA-transferase subunit A
MobB	WP_014753079.1	228	CoA transferase subunit B
MobC	WP_014753080.1	82	MbtH family NRPS accessory protein
MobD	None	319	TauD family dioxygenase
MobE	WP_014753082.1	477	non-ribosomal peptide synthase
MobF	WP_014753083.1	6470	non-ribosomal peptide synthase
MobG	WP_014753084.1	705	AMP-binding protein
MobH	WP_014753085.1	3895	non-ribosomal peptide synthetase
MobI	WP_014753086.1	344	acetyltransferase
MobJ	WP_014753087.1	444	SidA/IucD/PvdA family monooxygenase
MobK	WP_014753088.1	257	siderophore-iron reductase FhuF
MobL	WP_014753089.1	125	hypothetical protein
MobM	WP_081599059.1	662	acyl-homoserine lactone acylase
MobN	WP_014753091.1	810	TonB-dependent siderophore receptor
MobO	WP_014753092.1	265	Fe(3+)-hydroxamate ABC transporter permease FhuB

Table S2. Tistrellabactin A NMR chemical resonances and correlations

	Residue	Position	δ_H (m, *J* in Hz)	δ_C (m)	δ_N (ppm)	COSY	HMBC
N1	**Gln1**	NH_2	8.08 (d, 5.3)		99.7	1	1, 2, 5
1		Cα	4.18 (m)	51.05 (CH)		N1, 3	2, 3
2		Cβ	1.78 (m)	26.66 (CH_2)		1, 3	1, 3, 4, 5
3		Cγ	1.81, 1.97 (m)	29.74 (CH_2)		2	1, 2, 4, 5
4		<u>CO</u>NH_2		173.18			
N2		CO<u>NH_2</u>	6.94, 7.15 (s)		107.47	N2	3, 4
5		CO		168.28			
N3	**Ser**	NH	8.68 (d, 8.3)		113.13	6	5, 6, 8
6		Cα	5.08 (d, 8.0)	51.42 (CH)		N3, 7	5, 8
7		Cβ	4.13, 4.40 (m)	66.54 (CH_2)		6	6, 8, 41
8		CO		170.54			
N4	**Gra**	NH	8.87 (d, 3.2)		119.04	9	8, 9, 10, 13
9		Cα	4.07 (m)	55.87 (CH)		10, N4	10, 11, 13
10		Cβ	1.65 (m)	28.13 (CH_2)		9, 11	9, 11, 12, 13
11		Cγ	1.94 (m)	22.70 (CH_2)		10, 12	9, 10, 12, 13
12		Cδ	4.10 (m)	61.63 (CH_2)		11	10, 11
N5		NOH			315.27†		
N6		NO			367.69†		
13		CO		173.21			
N7	**Gln2**	NH	8.63 (d, 5.8)		113.94	14	13, 14, 15
14		Cα	3.88 (dt, 5.7, 9.4)	55.28 (CH)		N7, 15	13, 15, 16, 17, 18
15		Cβ	1.83, 1.91 (m)	25.88 (CH_2)		14, 16	14, 16, 17, 18
16		Cγ	2.20 (dt, 10.1, 7.6)	31.94 (CH_2)		15	14, 15
17		<u>CO</u>NH_2		174.00			
N8		CO<u>NH_2</u>	6.78, 7.39 (s)		108.84	N8	16, 17
18		CO		171.82			
N9	**Gln3**	NH	7.13 (m)		110.51	19	18, 19
19		Cα	4.15 (m)	52.78 (CH)		20, N9	18, 20, 21, 23
20		Cβ	1.86, 2.10 (m)	27.25 (CH_2)		19	19, 21, 22 23
21		Cγ	2.04, 2.12 (m)	32.00 (CH_2)		21	19, 20, 22
22		<u>CO</u>NH_2		175.05			
N10		CO<u>NH_2</u>	6.89, 7.58 (s)		111.07	N10	21, 22
23		CO		172.14			
N11	**OH-Asp**	NH	7.93 (d, 6.9)		111.47	24	23, 24, 25
24		Cα	4.48 (dd, 6.8, 1.8)	56.92 (CH)		N11, 25	23, 25, 28
25		Cβ	4.34 (m)	70.73 (CH)		24, 26	24, 28
26		Cβ-OH	6.38 (s)			25	
27		COOH	12.75 (s)				
28		CO		167.69			
N12	**Asn**	NH	7.45 (d, 10.1)		112.99	29	28, 29
29		Cα	4.62 (dt, 10.1, 3.9)	47.50 (CH)		N12, 30	28, 30, 31, 32
30		Cβ	2.32 (dd, 16.7, 4.3), 3.11 (d, 16.9)	34.85 (CH_2)		29	29, 31, 32
31		<u>C</u>ONH_2		173.31			
N13		CO<u>NH_2</u>	6.99, 8.00 (s)		111.33	N13	30, 31
32		CO		170.33			
N14	**OH-Orn**	NH	7.65 (d, 6.1)		114.48	33	33, 32
33		Cα	4.36 (m)	51.64 (CH)		N14, 34	32, 34, 35, 41
34		Cβ	1.58, 1.67 (m)	27.90 (CH_2)		33, 35	33, 36, 41
35		Cγ	1.41 (q, 7.6), 1.54 (m)	22.98 (CH_2)		34, 36	33, 34, 36
36		Cδ	3.38, 3.56 (dt, 13.7, 6.3)	46.10 (CH_2)		35	34, 35, 37
N15		NOH	9.63 (s)		175.59†		
37		CO		171.41			
38	**Hbu**	Cα	2.38, 2.54 (d, 6.3)	41.50 (CH_2)		39	37, 39, 40
39		Cβ	3.98 (sext, 6.3)	63.19 (CH)		38, 40	37, 38, 40
40		Cγ	1.06 (d, 6.2)	23.41 (CH_3)		39	37, 38, 39
41		CO		170.98			

†^{15}N measured indirectly. Chemical resonances shown account for wrapping around the spectral window (60 ppm wide).

Table S3. Tistrellabactin A TOCSY 2D NMR correlations

Assignment	TOCSY correlation
Glutamine 1	1, 2, 3, N1
Glutamine 1 NH_2	N2
Serine	6, 7, N3
Graminine	9, 10, 11, 12, N4
Glutamine 2	14, 15, 16, N7
Glutamine 2 NH_2	N8
Glutamine 3	19, 20, 21, N9
Glutamine 3 NH_2	N10
OH-Aspartic acid	24, 25, N11
Asparagine	29, 30, N12
Asparagine NH_2	N13
OH-Ornithine	33, 34, 35, 36, N14
3-hydroxybutyric acid	38, 39, 40

Table S4. Tistrellabactin A ^{1}H-^{15}N HMBC and HSQC data

	^{15}N (ppm)	^{1}H-^{15}N HMBC	^{1}H-^{15}N-HSQC	Assignment
N1	99.7	N1, 2, 3		Gln1 NH_2 (N-terminus)
N2	107.47	N2, 2, 3	N2	Gln1 NH_2 (side chain)
N3	113.13	N3, 6, 7	N3	Ser NH
N4	119.04	N4, 9, 10	N4	Gra NH
N5	75.27	N5, 11, 12		Gra NOH
N6	67.69	N6, 12		Gra NO
N7	113.94	N7, 14, 15	N7	Gln2 NH
N8	108.84	N8, 15, 16	N8	Gln2 NH_2
N9	110.51	N9, 14, 19, 20, 21	N9	Gln3 NH
N10	111.07	N10, 21	N10	Gln3 NH_2
N11	111.47	N11, 24, 25	N11	OH-Asp NH
N12	112.99	N12, 29, 30	N12	Asn NH
N13	111.33	N13, 30	N13	Asn NH_2
N14	114.48	N14, 33, 35	N14	Hbu-OH-Orn NH
N15	115.59	N15, 34, 36, 38, 40		Hbu-OH-Orn NOH

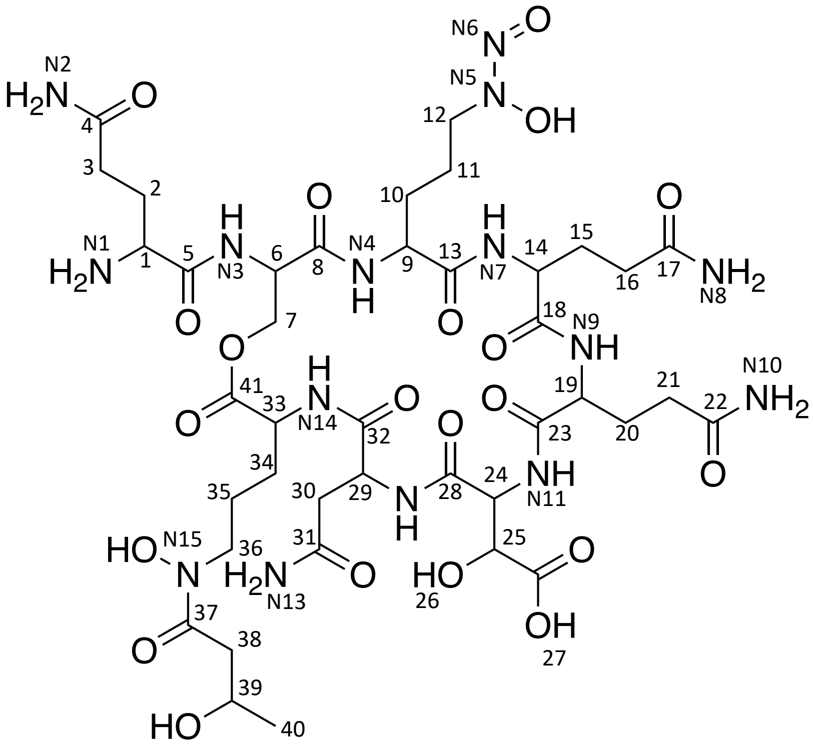

Tistrellabactin A

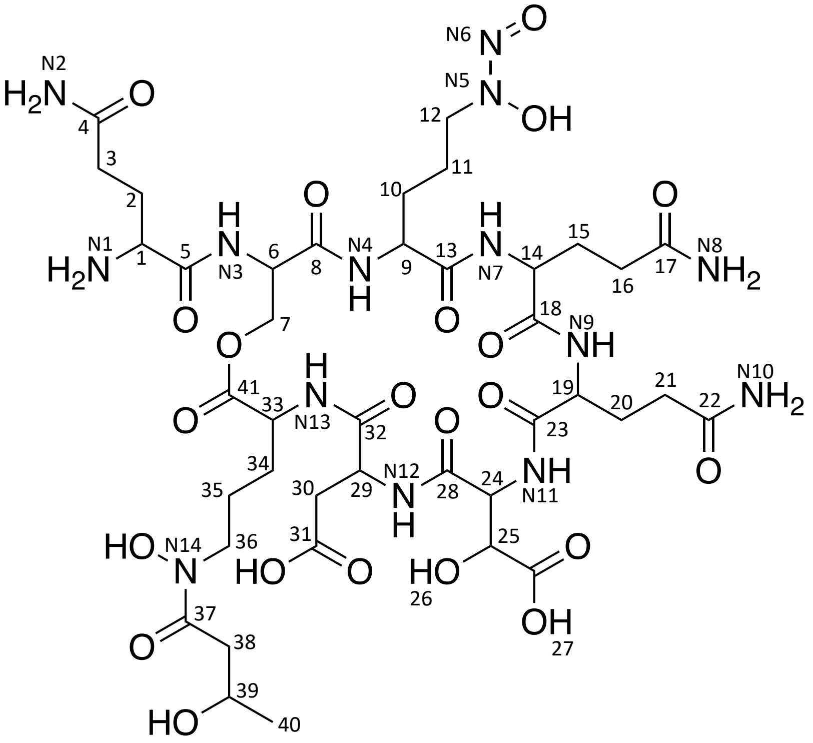

Tistrellabactin B

Table S5. Tistrellabactin B NMR ^{1}H, ^{13}C, ^{15}N chemical resonances

	Residue	Position	δ_H (m, *J* in Hz)	δ_C (m)	δ_N (ppm)
N1	**Gln1**	NH_2	8.18		
1		Cα	4.13	52.04 (CH)	
2		Cβ	1.83	27.41 (CH_2)	
3		Cγ	1.99, 2.05	29.91 (CH_2)	
4		<u>CO</u>NH_2		173.06	
N2		CO<u>NH_2</u>	6.97, 7.24		108.35
5		CO		168.24	
N3	**Ser**	NH	8.34		112.48
6		Cα	5.03	50.99 (CH)	
7		Cβ	4.12, 4.44	66.08 (CH_2)	
8		CO		170.28	
N4	**Gra**	NH	8.82		119.03
9		Cα	4.11	55.36 (CH)	
10		Cβ	1.66	28.11 (CH_2)	
11		Cγ	1.92	22.89 (CH_2)	
12		Cδ	4.09	61.52 (CH_2)	
N5		NOH			315.79†
N6		NO			368.24†
13		CO		173.34	
N7	**Gln2**	NH	8.56		114.53
14		Cα	3.91	55.00 (CH)	
15		Cβ	1.84, 1.90	26.02 (CH_2)	
16		Cγ	2.20	31.80 (CH_2)	
17		<u>CO</u>NH_2		173.96	
N8		CO<u>NH_2</u>	6.79, 7.37		108.77
18		CO		171.63	
N9	**Gln3**	NH	7.19		111.05
19		Cα	4.16	52.69 (CH)	
20		Cβ	1.89, 2.08	26.77 (CH_2)	
21		Cγ	2.03, 2.09	31.86 (CH_2)	
22		<u>CO</u>NH_2		174.69	
N10		CO<u>NH_2</u>	6.98, 7.57		111.10
23		CO		171.83	
N11	**OH-Asp**	NH	7.95		112.04
24		Cα	4.54	56.73 (CH)	
25		Cβ	4.31	70.76 (CH_2)	
26		Cβ-OH	6.21		
27		COOH	12.75		
28		CO		168.05	
N12	**Asp**	NH	7.46		112.96
29		Cα	4.71	47.71 (CH)	
30		Cβ	2.45, 3.02	35.57 (CH_2)	
31		COOH	12.90	172.20	
32		CO		169.94	
N13	**OH-Orn**	NH	7.83		115.88
33		Cα	4.35	51.84 (CH)	
34		Cβ	1.59, 1.67	27.81 (CH_2)	
35		Cγ	1.43, 1.54	22.58 (CH_2)	
36		Cδ	3.42, 3.53	46.13 (CH_2)	
N14		NOH	9.65		176.01†
37		CO		171.39	
38	**Hbu**	Cα	2.38, 2.52	41.49 (CH_2)	
39		Cβ	3.99	63.21 (CH)	
40		Cγ	1.07	23.39 (CH_3)	
41		CO		170.89	

†^{15}N measured indirectly. Chemical resonances shown account for wrapping around the spectral window (80 ppm wide).

References

(1) Reitz, Z. L.; Hardy, C. D.; Suk, J.; Bouvet, J.; Butler, A. Genomic analysis of siderophore beta-hydroxylases reveals divergent stereocontrol and expands the condensation domain family. *Proc Natl Acad Sci U S A* **2019**, *116* (40), 19805-19814. DOI: 10.1073/pnas.1903161116.
(2) Makris, C.; Carmichael, J. R.; Zhou, H.; Butler, A. C-Diazeniumdiolate Graminine in the Siderophore Gramibactin Is Photoreactive and Originates from Arginine. *ACS Chemical Biology* **2022**. DOI: 10.1021/acschembio.2c00593.
(3) Bhushan, R.; Bruckner, H. Marfey's reagent for chiral amino acid analysis: a review. *Amino Acids* **2004**, *27* (3-4), 231-247. DOI: 10.1007/s00726-004-0118-0.
(4) Blin, K.; Shaw, S.; Kloosterman, A. M.; Charlop-Powers, Z.; van Wezel, G. P.; Medema, M. H.; Weber, T. antiSMASH 6.0: improving cluster detection and comparison capabilities. *Nucleic Acids Res* **2021**, *49* (W1), W29-W35. DOI: 10.1093/nar/gkab335.
